# Prospective application of theoretical implementation frameworks to improve health care in hospitals — a systematic review

**DOI:** 10.1186/s12913-023-09609-y

**Published:** 2023-06-09

**Authors:** Rebecca Barnden, David A. Snowdon, Natasha A. Lannin, Elizabeth Lynch, Velandai Srikanth, Nadine E. Andrew

**Affiliations:** 1 Academic Unit, Peninsula Health, Frankston, VIC Australia; 2 Peninsula Clinical School, Central Clinical School, Faculty of Medicine, Nursing and Health Science, Monash University, Frankston, VIC Australia; 3 National Centre for Healthy Ageing, Melbourne, VIC Australia; 4 Department of Neuroscience, Faculty of Medicine, Nursing and Health Science, Monash University, Melbourne, VIC Australia; 5 Alfred Health, Melbourne, VIC Australia; 6 Caring Futures Institute, College of Nursing and Health Sciences, Flinders University, Adelaide, South Australia Australia

**Keywords:** Knowledge translation, Implementation frameworks, Implementation models, Implementation theories, Translation medical research

## Abstract

**Background:**

Health Service implementation projects are often guided by theoretical implementation frameworks. Little is known about the effectiveness of these frameworks to facilitate change in processes of care and patient outcomes within the inpatient setting. The aim of this review was to assess the effectiveness of the application of theoretical implementation frameworks in inpatient healthcare settings to change processes of care and associated patient outcomes.

**Method:**

We conducted a search in CINAHL, MEDLINE, EMBASE, PsycINFO, EMCARE and Cochrane Library databases from 1^st^ January 1995 to 15^th^ June 2021. Two reviewers independently applied inclusion and exclusion criteria to potentially eligible studies. Eligible studies: implemented evidence-based care into an in-patient setting using a theoretical implementation framework applied prospectively; used a prospective study design; presented process of care or patient outcomes; and were published in English. We extracted theoretical implementation frameworks and study design against the Workgroup for Intervention Development and Evaluation Research (WIDER) Checklist and implementation strategies mapped to the Cochrane Effective Practice and Organisation of Care (EPOC) taxonomy. We summarised all interventions using the Template for Intervention Description and Replication (TIDieR) checklist. We appraised study quality using the Item bank on risk of bias and precision of observational studies and the revised Cochrane risk of bias tool for cluster randomised trials. We extracted process of care and patient outcomes and described descriptively. We conducted meta-analysis for process of care and patient outcomes with reference to framework category.

**Results:**

Twenty-five studies met the inclusion criteria. Twenty-one used a pre-post (no comparison), two a pre-post with a comparison, and two a cluster randomised trial design. Eleven theoretical implementation frameworks were prospectively applied: six process models; five determinant frameworks; and one classic theory. Four studies used two theoretical implementation frameworks. No authors reported their justification for selecting a particular framework and implementation strategies were generally poorly described. No consensus was reached for a preferred framework or subset of frameworks based on meta-analysis results.

**Conclusions:**

Rather than the ongoing development of new implementation frameworks, a more consistent approach to framework selection and strengthening of existing approaches is recommended to further develop the implementation evidence base.

**Trial registration:**

CRD42019119429.

**Supplementary Information:**

The online version contains supplementary material available at 10.1186/s12913-023-09609-y.

## Background

The importance of implementing evidence-based practice in healthcare is widely accepted [1]. However, gaps between evidence and practice are consistently reported in the clinical and health services literature, even when a strong evidence base is established [2–4]. The study of implementing evidence into clinical practice is an emerging field that has grown rapidly over the past two decades [5].

Health service implementation projects include implementation strategies designed to change health professional behaviour and optimise delivery of the evidence-based intervention being targeted [6]. The success or otherwise of health service implementation projects can be measured through changes in process of care and patient outcomes. Changes in process of care are likely to be the result of the implementation strategies targeting health professional behavior change [7], whereas patient outcomes (health outcomes and satisfaction) are likely the result of a complex interaction between the process of care, the organizational structure where this care is provided, patient characteristics, and the nature of the clinical intervention itself [8].

Over 100 different implementation theories, models and frameworks have been described to guide implementation research [9]. Throughout this review these implementation theories, models and frameworks will be collectively described as *theoretical implementation frameworks*. Despite the large number of published theoretical implementation frameworks available, many with considerable overlap of constructs, there is limited information to guide framework selection [9]. As a result, selecting a framework to use in implementation research can be a challenging task [10, 11]. In a recent interview-based qualitative study, 24 international implementation researchers and practitioners identified barriers to theoretical implementation framework selection. Barriers included: inconsistent language, poor fit with the implementation context, a lack of appropriate measures for key constructs, and limited empirical evidence of effectiveness. [11]. It would be valuable to provide healthcare administrators, clinicians and researchers with guidance on how to select from among theoretical implementation frameworks. Such guidance should consider both the needs of the implementation research, the context in which implementation will occur, and the established effectiveness of the theoretical implementation framework or frameworks to create change in practice and patient outcomes [10].

A 2012 narrative review described sixty-one different theoretical implementation frameworks [12]. Frameworks in this review were organised and presented based on the flexibility of the framework constructs to be applied to a range of implementation activities and contexts *(defined by the authors as construct flexibility*), the framework’s focus on dissemination and/or implementation, and the level at which the framework is designed to operate (system, community, organisational, and/or individual level). These categories were presented as a starting point to help guide theoretical framework selection [12]. A follow-up paper used bibliometric citation analysis to establish the citation rate of each of these sixty-one theoretical frameworks, with average citations ranging from 0.7 to 103.3 per year and suggested that citation frequency could be used to guide theoretical implementation framework selection [13]. However, frequency of past use without considering important criteria such as study design and the effectiveness of theoretical implementation frameworks does not provide an evidence based approach to framework selection.

In 2015, a survey of 223 international implementation scientists was conducted to understand what theoretical implementation frameworks were being used and the criteria used for framework selection. Over 100 different theoretical frameworks were reported in this study [9], and the authors concluded that framework selection was “*often haphazard or driven by convenience or prior exposure*” [9]. To assist with framework selection Nilsen [14] proposed a taxonomy to categorise the large number of theoretical implementation frameworks into framework categories based on three broad overarching aims: (i) describing and/or guiding the process of implementing evidence into practice (*process models*); (ii) understanding and/or explaining what influences the outcomes of implementation projects (*determinant frameworks, classic theories, implementation theories*); and (iii) evaluating implementation (*evaluation frameworks*)”. *Process models* outline the steps to follow in an implementation project (e.g. the Knowledge-to-Action Model) [15]. *Determinant frameworks* include determinants thought to influence implementation success and include enablers and barriers that may impact implementation outcomes (e.g. the Theoretical Domains Framework) [16, 17]. C*lassic theories* refer to theories developed in fields such as psychology and sociology with the aim of describing or explaining how change occurs, rather than actually implementing the change (e.g. Theory of diffusion) [18]. *Implementation theories* have been developed or adapted to understand and explain aspects of implementation (e.g. COM-B) [19]. Evaluation frameworks are used post implementation to evaluate success (e.g. RE-AIM) [20]. Given the large number of theoretical implementation frameworks available, often with similar features and overlap of constructs, selecting a framework from one or more of these framework categories based on the specific aims of the implementation activity may provide a useful approach.

Theoretical implementation frameworks are designed to be used prospectively to guide the implementation process and/or to identify implementation enablers and barriers. The prospective use of theoretical implementation frameworks is likely to be particularly valuable in complex environments such as inpatient and hospital settings with diverse patient populations and multidisciplinary teams providing care across distinct units and program areas [21]. Additional guidance in relation to theoretical implementation framework selection in these complex settings would thus be valuable for clinicians and researchers and may enhance implementation success. Little is known about the effectiveness of different theoretical implementation frameworks from different framework categories when applied prospectively in health service implementation research. The aim of this review is to help guide the prospective selection of theoretical implementation frameworks by assessing the effectiveness of such frameworks in changing processes of care, and where available patient outcomes, in inpatient healthcare settings.

## Methods

### Protocol and registration

This systematic review has been reported with reference to the Preferred Reporting Items for Systematic Reviews and Meta-Analysis (PRISMA) guidelines for systematic reviews and was registered prospectively in the PROSPERO database (registration number: CRD42019119429, date of registration 12/06/2019). One minor deviation to our registered protocol was the use of the RTI Item bank [22] and the revised Cochrane risk of bias tool for cluster randomised trials [23] to assess risk of bias instead of the Downs and Black [24] tool. This change occurred as these tools were better suited to the study designs used in the implementation studies included in this review.

### Eligibility criteria

Studies which presented implementation research were eligible if they 1) involved the implementation of evidence-based care guided prospectively by an established theoretical implementation framework; 2) were conducted in an inpatient health services setting (hospital or inpatient rehabilitation); 3) used a prospective controlled study design including pre-post design, cluster randomised controlled trial, prospective cohort or randomised controlled trial, 4) presented process of care outcomes, and where available patient outcomes; 5) were written in English; and 6) the full text article was available.

Studies were ineligible if they met any of the following criteria: 1) publications prior to 1995, the year after which theoretical implementation frameworks began to appear in the literature [13]; 2) conference proceeding; 3) protocol registrations; and 4) non-peer reviewed sources.

For the purpose of this review we included any published theoretical implementation framework that considered two or more implementation stages, and/or inter-related constructs, proposed by the authors to contribute to the success or failure of implementing evidence into health service practice.

### Information sources

A search was completed in the CINAHL, MEDLINE, EMBASE, PsycINFO, EMCARE and Cochrane Library databases with dates restricted to 1^st^ January 1995 to 15^th^ June 2021.

### Search

The PICO (Population, Intervention, Comparison and Outcome) framework was used to define the question for this systematic review. The concepts of ‘population’ and ‘intervention’ were used to establish the search terms for this review. Outcome terms were not included as we did not wish to limit outcomes to specific types. ‘Population’ was searched using key words and synonyms relating to receiving healthcare in an inpatient setting or hospital. The theoretical implementation framework was considered to be the ‘intervention’. This concept was searched using terms commonly used in the implementation science literature and by using a number of commonly cited theoretical implementation frameworks and acronyms that have been designed for prospective use. The selection of intervention search terms was guided by earlier work by author (EL) and colleagues [10]. The full search strategy is provided in Supplemental Table 1.

### Study selection

Two reviewers (RB and DS) independently screened records by title and abstract using Covidence software [25]. Where eligibility could not be determined by title and abstract review the full text article was obtained to assess eligibility and was again assessed independently by two reviewers (RB and DS). Disagreements in eligibility of an article were resolved through discussion between the two reviewers. Where consensus could not be reached a third reviewer (NA) was consulted.

### Data collection process

A purposefully designed data collection tool based on the Workgroup for Intervention Development and Evaluation Research (WIDER) [26] checklist (Supplemental Table 2) was used to collect data on the theoretical implementation framework used in each study. This included describing the individual components of the theoretical implementation framework and categorising the framework as either a process model, determinant framework, classic theory, implementation theory, evaluation framework, or a combination [14]. For each implementation strategy used within each study the WIDER [26] checklist was used to assess reporting of the following: characteristics of those delivering the implementation strategy; characteristics of the recipients of the implementation strategy; the mode of delivery of the implementation strategy; and the intensity of the implementation strategy. The implementation strategies included in each study were also mapped to the Effective Practice and Organisation Care (EPOC) taxonomy (Supplemental Table 3) [27].

The intervention being implemented in each study was described using the items included in the template for intervention description and replication (TIDieR) checklist [28] (Supplemental Table 4) and included: a brief description of the intervention; materials; procedures; health professionals involved in delivering intervention; clinical setting and patient population; dose; and any tailoring of the intervention. In addition, the source of evidence supporting the intervention was recorded.

Quantitative process of care and patient outcomes (where reported) were recorded. Process of care outcomes refer to both the delivery of healthcare by healthcare staff (e.g. the proportion of staff who completed an administrative activity), and the receipt of healthcare by patients (e.g. the proportion of patients that received a recommended assessment or treatment) [8]. Patient outcomes could include clinical outcomes (e.g. pain intensity, hospital acquired infection), patient reported outcome measures (PROMs) such as quality of life, or patient reported experience measures (PREMs) such as satisfaction with care [8]. Contact with authors was attempted to obtain any missing process of care and patient outcome data. Where studies reported process and/or patient outcomes as a median (and interquartile range) these data were used to estimate the sample mean and standard deviation [29] so that data from these studies could be included in the meta-analysis. In studies where we were unable to extract all of the required data from the primary publication we hand searched the reference list of the primary publication to identify other related publications by the study authors (i.e. methods papers or protocol papers) and where available used these papers to extract available missing data.

### Risk of bias in individual studies

All included studies were critically appraised for methodological quality and risk of bias independently by two reviewers (RB and DS). Domains from the Item bank for assessment of risk of bias for observational studies of interventions or exposures [22] and the revised Cochrane risk of bias tool for cluster randomised trials [23] were assessed as high, low or unclear risk with justification given for judgement. Any disagreements between reviewers were resolved through discussion. Where consensus could not be reached a third reviewer (NA) was consulted.

### Synthesis of outcome results

#### Categorisation of studies

For the primary analysis, theoretical implementation frameworks were categorised based on the Nilsen taxonomy [14] as process models, determinant frameworks, classic theories, implementation theories, evaluation frameworks, or a combination to allow comparison of choice, use, and outcome by framework category. This approach allowed a comparison by framework category, rather than comparison between a large number of individual theoretical implementation frameworks. Further categorisation was completed based on study design: pre/post design (no comparison), pre/post design with a comparison, prospective cohort, or randomised or cluster randomised controlled trials. Primary and secondary process of care outcomes were also categorised into: screening and assessment; providing recommended care; or other.

#### Meta-analysis

Odds ratios (OR) for dichotomous outcomes and standardised mean differences (SMD) for continuous outcomes were calculated for both primary process of care and primary patient outcomes. For process of care outcomes an OR > 1 and SMD > 1 favours the post-implementation period. For patient outcomes an OR < 1 and SMD of < 1 favours the post-implementation period. Where sufficient data were available, meta-analysis was performed using Review Manager (Computer Program version 5.4) [30] within the pre-specified sub-groups mentioned above using the inverse variance method and random effects model. Studies with a proportion of 0% in the pre-implementation period were excluded from the meta-analysis.

Statistical heterogeneity was assessed using the I^2^ statistic. Where considerable heterogeneity was identified (i.e. I^2^ > 75%) additional sub group analyses were explored.

## Results

### Study selection

**Fig. 1:**
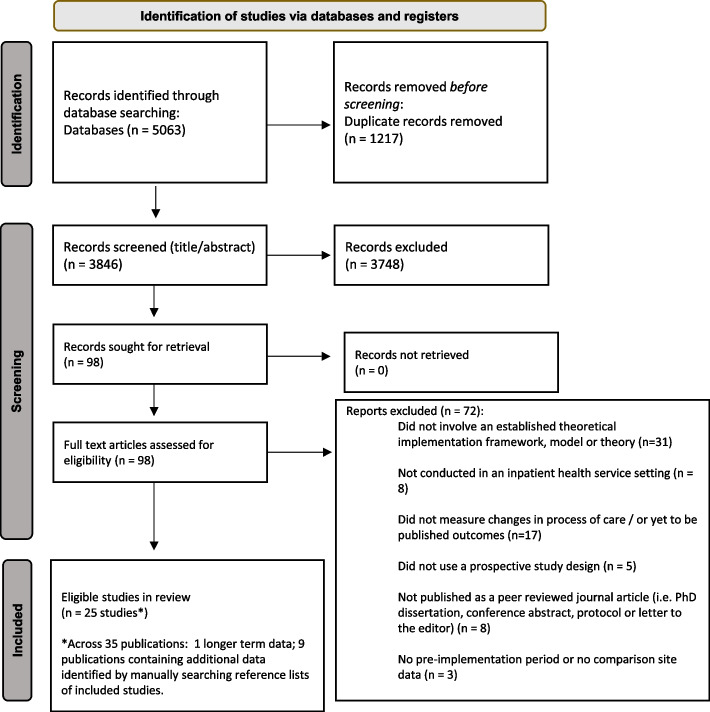
Article selection process

Our initial search yielded 5,063 records. Ninety-eight articles were retrieved for full text review following application of eligibility criteria to title and abstract of which 25 studies (across 35 publications) fulfilled the inclusion criteria (Fig. 1). Agreement between the two reviewers for screening full text articles was very good (k = 0.951, 95%CI 0.884–1.000). List of full text articles excluded, with reasons is included in Supplemental Table 5.

### Study characteristics

All 25 included studies (Supplemental Table 6) involved an implementation project that aimed to achieve compliance with a research or guideline-based intervention (Supplemental Table 7) across a range of inpatient clinical areas. Only four studies included a measure of implementation sustainability.

Eleven theoretical implementation frameworks were prospectively applied in the twenty-five included studies. These were categorised into the following:

(i) process models (*n* = 5) including:
  - Agile Implementation Model [31];
  - Grol and Wensing Model [32];
  - Grol’s 5-step Implementation Model [33];
  - JBI Evidence Implementation Framework [34]; and
  - Knowledge to Action (KTA) [15]
(ii) determinant frameworks (*n* = 5) including:
  - Active Implementation Frameworks of the National Implementation Research Network (NIRN) [35];
  - the Consolidated Framework for Implementation Research (CFIR) [36];
  - the Promoting Action on Research Implementation in Health Services (PARiHS) [37, 38] and a revised version i-PARiHS [39], and
  - Theoretical Domains Framework (TDF) [16, 17]; and
(iii) classic theories (*n* = 1):
  - Model of Diffusion of Innovations in Service Organisations) [40].

**Fig. 2:**
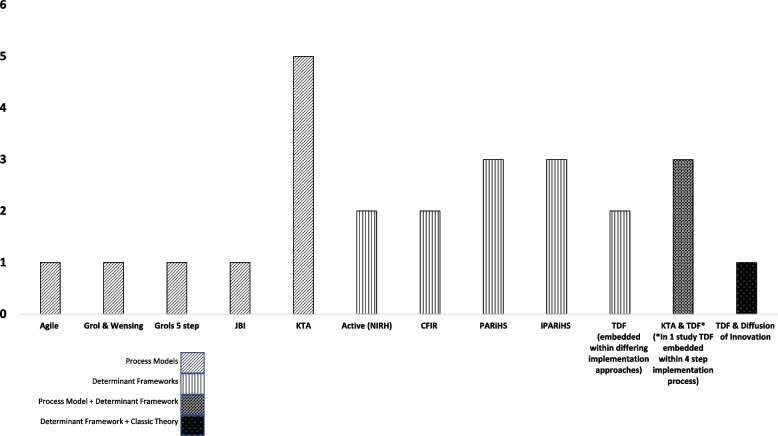
Frameworks applied prospectively in included studies

Process models were used across nine studies: five implemented guidelines aimed at minimising hospital acquired harms including central line infections, delirium, falls and malnutrition; one targeted best practice pain assessments; one aimed at increasing compliance with a surgical safety checklist; and one compliance with the provision of evidenced based stroke care (Supplemental Table 6). The KTA was the most commonly used process model (Fig. 2).

Determinant frameworks were used across 12 studies: six involving guideline implementation to minimise hospital acquired harms including malnutrition, pressure injury, falls, integrated risks of harm, and incorrect nasogastric tube placement; one implementing a perioperative surgical checklist; two focused on increasing compliance with best practice pain assessments; and three implementing best practice treatment provision relating to nutrition, non-pharmacological management of delirium, and the management of cancer related fatigue (Supplemental Table 6). The PARiHS and iPARiHS were the most common used theoretical implementation frameworks from the determinant framework category (Fig. 2).

Four studies reported the use of two theoretical frameworks in a complementary manner. One used a determinant framework (TDF) with a classic theory (Model of Diffusion of Innovations in Service Organisations) in a study aimed at increasing guideline compliance for managing mild traumatic brain injury. Another three studies used both a process model (KTA) with a determinant framework (TDF). Two of these studies focused on compliance with evidenced based nutrition care and another on using gait speed as part of physical therapy assessments (Supplemental Table 6).

The implementation studies targeted a range of health professionals including physicians [41], surgeons [42–44], anaesthesiologists [43, 44], medical staff [45–52], nurses [41–45, 47–62], nurse assistants [47, 48, 57, 61], dietitians [42, 48, 52, 61], physical therapists [47, 50, 59, 61, 63, 64], occupational therapists [50, 59, 61], psychologists [50], multidisciplinary teams [42, 47, 50, 52, 61, 65, 66], as well as support services team members including food service staff, [49] and biomedical and sterile processing technicians. [43] Studies targeting more than one professional group or multidisciplinary teams more often used a determinant framework or a combination of more than one framework (Supplemental Fig. 1).

**Fig. 3:**
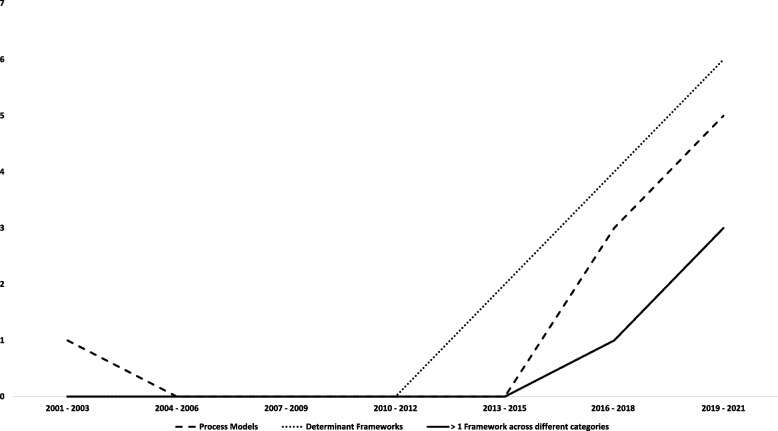
Publication year of included studies by framework type (1995—2021)

Of the 25 included studies only one study that used a process model was published prior to 2013 [60]. The remaining 24 studies were published within the past decade. All studies published between 2013 and 2017 used either a process model or determinant framework. Since 2018, studies have used either a process model or determinant framework, or a combination of more than one theoretical implementation framework from across different framework categories (Fig. 3).

The majority (84%) of studies used a pre-post study design with no comparison site, where the intervention unit(s) was a historical pre-implementation control. Of these, eight studies used a process model, ten a determinant framework and three a combination of framework categories (e.g. process model and determinant framework). Two studies used a pre-post study design with a concurrent comparison unit; both of these studies used a determinant framework. Two studies used a cluster randomised controlled trial study design: one used a process model; and the other a combination of theoretical implementation frameworks from across different framework categories.

### Risk of bias

Quality assessment was completed for all studies and organised by framework category (Supplemental Table 8). Risk of bias was generally high across all studies and framework categories. Quality was overall very similar across studies using a process model or determinant framework. However, the quality of the studies that used more than one theoretical implementation framework from different framework categories was overall better than those studies that used a theoretical implementation framework from only one framework category (Supplemental Fig. 2).

### Quality of implementation approach

All included studies stated the theoretical implementation framework used and most detailed using all components of the framework to guide their implementation study (Supplemental Table 9). No study detailed the rationale or criteria used for selecting a specific theoretical implementation framework over another. Fidelity to the implementation strategy was generally not reported, or when included was frequently limited to compliance with attending training or education sessions [47, 51, 53, 58, 61]. Only three studies reported overall compliance with the implementation approach [45, 48, 49].

All studies reported the implementation strategies used. Studies that used a process model mostly used: local opinion leads (89%); audit and feedback (67%); educational materials (56%); reminders (56%); and tailored interventions (56%). Studies that used a determinant framework mostly used: local opinion leads (92%); educational materials (75%); educational meetings (75%); reminders (67%); and tailored interventions (67%). Studies that used more than one framework in combination mostly used: tailored interventions (100%); and reminders (75%) (Supplemental Table 10). The level of detail regarding the method of delivery of individual implementation strategies varied. Overall reporting of the characteristics of those delivering and receiving the implementation strategy, the mode of delivery, and intensity of the implementation strategy was poor. The reporting was better in the studies that used theoretical implementation frameworks from two different framework categories (Supplemental Table 10).

### Results of individual studies organised by framework category

#### Process of care outcomes

Process of care outcomes were associated with one or more of the following themes: 1) completing screening and assessments as recommended; 2) providing recommended care; and 3) other. Supplemental Table 11 presents a summary of all primary and secondary process of care outcomes.

**Fig. 4:**
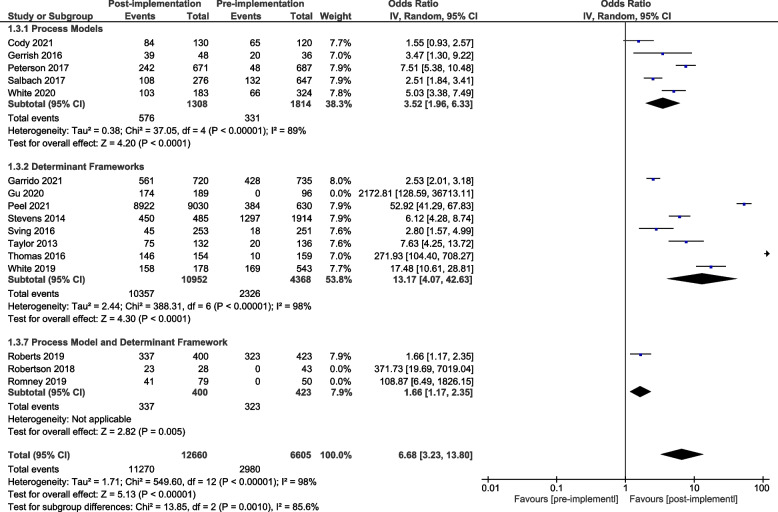
Process of care outcomes by framework category: pre-post study design

**Fig. 5:**
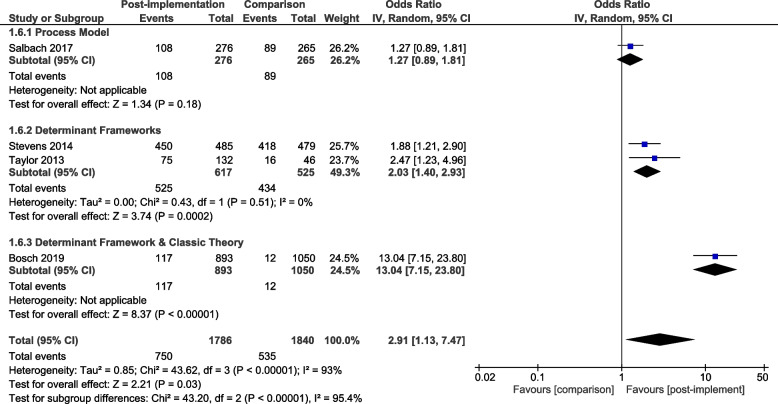
Process of care outcomes by framework category: control group comparison

A meta-analysis of all primary process of care outcomes, organised by framework category, is presented for the pre-implementation / post-implementation periods (Fig. 4) and for the post-implementation / comparison units (Fig. 5). Considerable heterogeneity was observed across all analyses (I^2^ 98%—93%) as were wide confidence intervals. To better understand the role of heterogeneity, we completed pre-specified sub-group analyses where sufficient data were available for: pre-post process of care outcomes relating to completing screening and assessments (supplemental Fig. 3) and providing recommended care (Supplemental Fig. 4). These additional subgroup analyses did not alter the results or have much impact on the heterogeneity. A meta-analysis was additionally completed for the pre-implementation / post-implementation periods for the three studies that reported sustainability data (Supplementary Fig. 5).

In the pre-post analysis using a single determinant framework resulted in enhanced compliance with recommended processes of care when compared to using a single process model or when combining two theoretical implementation frameworks from differing framework categories (Fig. 4). In the post implementation / comparison control analysis there was a signal that the use of a single determinant framework may result in enhanced compliance with recommended processes of care when compared to using a single process model and that the use of two theoretical implementation frameworks from across different framework categories may further enhance compliance (Fig. 5).

#### Patient outcomes

Fifteen of the studies included patient outcomes. The majority were adverse events and negative outcomes including: 1) hospital acquired infections; 2) pressure injuries; 3) falls; 4) delirium; 5) inadequate nutrition or prolonged time to commencing a full diet post-surgery; and 6) pain intensity. Outcome were reported as proportions (e.g. patients experiencing the outcome), or continuous measures (e.g. a continuous pain scale or time to an event). One study reported patient perceived ability to manage cancer-related fatigue [62]. Supplemental Table 12 presents a summary of all patient outcomes.

**Fig. 6:**
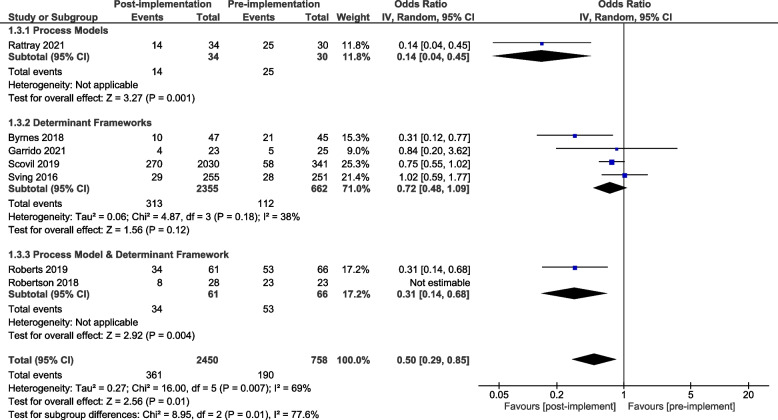
All patient outcomes (OR) by framework category – pre-post study design

**Fig. 7:**
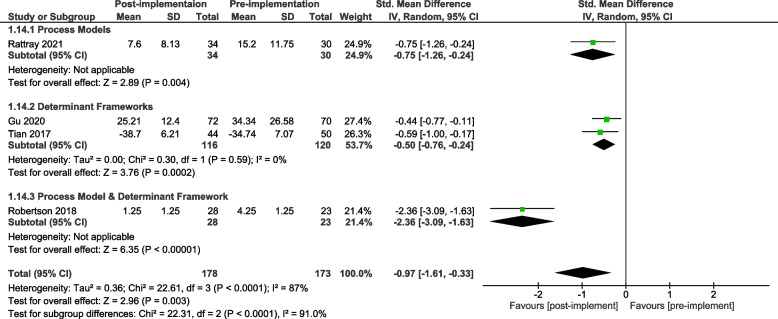
All patient outcomes (SMD) by framework category – pre-post study design

A meta-analysis of all primary patient outcomes, organised by framework category, is presented for the pre-implementation / post-implementation periods for both dichotomous (Fig. 6) and continuous (Fig. 7) outcomes. It should be noted that two studies [48, 51] included both dichotomous and continuous data and have been included in both of these figures.

## Discussion

This review provides preliminary evidence of the utility of using framework categories to guide selection of individual theoretical implementation frameworks to support implementation of evidence-based care in inpatient settings. Eleven different theoretical implementation frameworks were used in the studies included in this review. All were mapped to three framework categories: (i) process models, (ii) determinant frameworks and (iii) classic theories. The KTA was the most commonly used theoretical implementation framework from the process model category and the PARiHS and iPARiHS the most common theoretical implementation framework from the determinant framework category.

While findings of the review suggest the importance of these two frameworks, there was a lack of discussion in published work to date as to why one framework was selected over potential others. No authors provided a rational for their choice of theoretical implementation framework, or for selecting a framework from a particular category. Findings from the review suggest no correlations between framework category selection and study design or study size (single verses multisite). However, theoretical implementation frameworks from the determinant category were used more often in studies targeting multidisciplinary teams.

Despite the large number of frameworks there was considerable overlap in the theoretical constructs that underpinned many of these implementation approaches, particularly within framework categories. Although differing terminology was used across the theoretical implementation frameworks to define these constructs, there appears to be common themes that have evolved over time. It is likely that the limitations of early theoretical implementation frameworks have informed the iterative development of later frameworks, with concurrent work in this space leading to the development of similar, but varying frameworks. The common features and considerable overlap of constructs may have contributed to the variability in framework selection and thus supports theoretical implementation framework selection being guided by framework category.

A number of studies included in this review used recently developed process models and determinant frameworks, with the most recent first published in 2020. The ongoing creation of new frameworks, often with very similar constructs to those already freely available, is redundant without clear identification of the gap that a new framework is addressing. A more consistent approach to reporting the criteria used for framework selection, and where applicable clear justification for the need for a new implementation framework is urgently needed to further develop the evidence base to guide framework selection.

Studies included in this review used a number of implementation strategies. Studies that used a process model or determinant framework most frequently included the strategy of local opinion leads, whereas studies that used more than one framework from across framework categories most frequently included tailored implementation interventions. From the results of this review we are unable to make any firm recommendations regarding the use of a particular theoretical implementation framework or framework category to change process of care and patient outcomes.

However, findings suggest that when using a single theoretical implementation framework, the use of a determinant framework may result in enhanced compliance with recommended processes of care when compared to a process model. Further, there may be merit in combining more than one theoretical implementation framework from across framework categories to change process of care and associated patient outcomes when compared with using a theoretical implementation framework from a single framework category. Intuitively it would make sense to combine a process model to guide the overall implementation approach, together with a determinant framework that considers the context and population specific enablers and barriers. Unfortunately, there was insufficient data to draw a reliable conclusion from our systematic review.

The frameworks used most frequently in combination were the KTA with the TDF (3 studies) [49, 51, 67]. The KTA framework includes a step in the action cycle to “assess barriers”, and in one of these studies the determinant TDF was used for this specific step [51]. In the other two studies [49, 67] the TDF was used to assess enablers and barriers to inform the implementation approach, with the KTA framework used to guide the implementation process. A further study [45] used the determinant TDF and the classic theory the Model of Diffusion of Innovation in service organisations in a complementary manner to assess enablers and barriers and design the implementation approach. To understand the effectiveness and potential gains of applying two frameworks, further research is needed to evaluate the effects of combining two or more theoretical implementation frameworks from different framework categories.

Although all of the implementation studies included in this review used research or guideline based interventions, the overall quality of the study designs was poor, and most used a pre-post design introducing greater bias to interpreting the estimates of effectiveness [68]. Further, the majority of studies included in this review used a pre-implementation historical control, introducing the potential for imbalance in distribution of characteristics that may influence outcome alongside selection and temporal biases. There were greater differences observed post-implementation in these studies when compared to the few studies that included a comparison control site suggesting the potential impact of secular trends. Study design additionally limited interpretation of patient level outcomes with patient characteristics infrequently accounted for in the analysis. To better understand the effectiveness of implementing healthcare interventions, more robust implementation study designs are needed. The recent studies by Bosch [45] and Salbach [59] included in this review provide examples of such approaches. In situations where it is only feasible to do a pre-post design, such as single site pilot studies, the quality of the study could be strengthened by using approaches such as multiple baseline assessment, blinding outcome assessors to timepoint, using comparison control sites, case mix adjustment for patient characteristics, and more complex analysis approaches, such as interrupted time series.

### Limitations

For pragmatic reasons our search strategy was limited to papers with a theoretical implementation framework mentioned in the title or abstract. Implementation research studies that used a theoretical framework prospectively, but did not refer to its use in the title or abstract, were not captured in this review. Additionally, given the wide range of terminology used to describe the translation of evidence into practice and large number of theoretical frameworks available, it is possible that our search terms may not have covered all possible key word combinations used in the implementation literature and in the description of the numerous theoretical implementation frameworks.

All but one early study demonstrated improvements in process of care outcomes. It is possible that there may be a number of implementation research studies that did not find significant results or improvements in outcome that may not have been published, introducing a potential publication bias to this review. The perceived quality of implementation projects and study design makes this review particularly vulnerable to this type of bias. Additionally, the overall quality of studies included in this review was poor, limiting the strength of the findings of this review.

Ten studies failed to report sufficient data to perform odds ratio and /or standardised mean difference analysis. Contact with all authors was attempted to obtain this missing information, but responses (and additional data) were not forthcoming from five of these authors. These five studies could not be included in the meta-analysis because of missing data which we acknowledge could have introduced reporting bias. Reporting studies against guidelines such as TIDieR [28] and the Standards for Reporting Implementation (STaRI) statement [6] will reduce the likelihood of missing data in future systematic reviews on this topic.

## Conclusion

The continuing emergence of theoretical implementation frameworks suggests that those involved in implementation are increasingly looking to theoretical implementation frameworks to inform the design of implementation research studies. The use of so many different frameworks makes the comparison of implementation approaches, strategies and outcomes difficult. Grouping different implementation frameworks into framework categories provides preliminary evidence that framework selection by category may be effective. Additional studies using theoretical implementation frameworks from multiple framework categories may assist with establishing consensus on which implementation frameworks, or subset of frameworks, best supports successful implementation. Establishing such consensus, together with a more consistent and considered approach to reporting the criteria used for framework selection, would further develop the evidence base of implementation.

## Supplementary Information

**Additional file 1: Supplemental Table 1. **Search strategy used for all information sources. **Supplemental Table 2.** WIDER recommendations checklist. **Supplemental Table 3.** EPOC taxonomy - Implementation strategies for category: Implementation strategies targeted at healthcare workers. **Supplemental Table 4.** Template for Intervention Description and Replication (TIDieR) checklist. **Supplemental Table 5.** List of excluded studies along with reasons for exclusion. **Supplemental Table 6.** Summary table of included studies. **Supplemental Table 7.** TIDieR Table. **Supplemental Table 8.** Risk of bias across included studies. **Supplemental Table 9.** Adapted WIDER checklist with studies organised by framework category. **Supplemental Table 10.** Implementation strategies used within individual studies mapped to EPOC taxonomy and scored based on elements from the WIDER checklist. **Supplemental Table 11.** All process of care outcomes. **Supplemental Table 12.** All patient outcomes. **Supplemental Figure 1.** Studies targeting single verses multiply professional groups by framework category. **Supplemental Figure 2.** Summary plot risk of bias by framework category. **Supplemental Figure 3.** Screening and assessment process of care outcomes by framework category: pre-post study design. **Supplemental Figure 4.** Providing recommended care process of care outcomes by framework category: pre-post study design. **Supplemental Figure 5.** Process of care outcomes by framework category: sustainability comparison pre-post study design.

## Data Availability

All data generated or analysed during this study are included in this published article (and its supplementary information files).

## Abbreviations

CFIR: Consolidated Framework for Implementation Research
COM-B: Capability, Opportunity, Motivation, Behaviour Model
EPOC: Cochrane Effective Practice and Organisation of Care
JBI: Joanna Briggs Institute
KTA: Knowledge to Action
NIRN: National Implementation Research Network
PARiHS: Promoting Action on Research Implementation in Health Services
i-PARiHS: Integrated Promoting Action on Research Implementation in Health Services
OR: Odds ratios
PICO: Population, Intervention, Comparison and Outcome framework
PRISMA: Preferred Reporting Items for Systematic Reviews and Meta-Analysis
PREMs: Patient reported experience measures
PROMs: Patient reported outcome measures
RE-AIM: Reach, Effectiveness, Adoption, Implementation, and Maintenance
SMD: Standardised mean differences
TDF: Theoretical Domains Framework
TIDieR: Template for Intervention Description and Replication
WIDER: Workgroup for Intervention Development and Evaluation Research
