# Supplementary material for: Prospective application of theoretical implementation frameworks to improve health care in hospitals — a systematic review
Source: BMC Health Serv Res. 2023 Jun 9;23:607. doi: 10.1186/s12913-023-09609-y (PMC10257296; doi:10.1186/s12913-023-09609-y)

### ***Supplemental Table 1 - Search Strategy used for all information sources***

| Search Terms  (in: title and abstract) | MeSH | |
| --- | --- | --- |
|  | CIHAHL | MEDLINE, EMBASE PsychINFO, EMCARE and COCHRANE |
| Intervention Terms:  Implementation framework  Implementation model  Implementation theory  Knowledge translation  Translation medical research  Theoretical Domains Framework  TDF  Consolidated Framework for Implementation Research  Consolidated framework  CFIR  Promoting Action on Research Implementation in Health Services  PARIHS  Integrated Promoting Action on Research Implementation in Health Services  iPARIHS or i-PARIHS  Knowledge to action  Conceptual model of evidenced-based implementation in public service sectors  Conceptual model of implementation research  Interactive systems framework | Translations | Translation Research |
| Population Terms:  Inpatient*  Hospital*  Tertiary centre  Ward  Postoperative or post operative  Postsurgical or post surgical  NOT  General practice  General practitioner  Primary care | Hospital patient  Inpatients  Hospitals | Hospital patient  General hospital  Hospital |
| Design Terms  NOT  Case study  Case series  Review  Editorial  Narrative |  |  |

Limits:  English Language; Human; Year = 1995 – current; Full text

Key words and synonyms for each concept were combined using the ‘OR’ operator. The concepts of intervention and population were then combined using the ‘AND’ operator.

### ***Supplemental Table 2: WIDER(1) Recommendations Checklist***

| **Wider Recommendation** | **Supplementary Recommendations** | **Yes** | **No** |
| --- | --- | --- | --- |
| Detailed description of Intervention in published papers | 1. Characteristics of those delivering the interventions |  |  |
|  | 1. Characteristics of the recipients |  |  |
|  | 1. The setting |  |  |
|  | 1. The mode of delivery |  |  |
|  | 1. The intensity |  |  |
|  | 1. The duration |  |  |
|  | 1. Adherence/fidelity to delivery protocols |  |  |
|  | 1. Detailed description of the intervention content provided to each study group |  |  |
| Clarification of assumed change process and design principles | 1. The intervention development |  |  |
|  | 1. The change techniques used in the intervention |  |  |
|  | 1. The causal process targeted by these change techniques |  |  |
| Access to intervention manuals / protocols | Have protocols or manuals been submitted for publication to make these supplementary materials easily accessible (i.e. online) |  |  |
| Detailed description of active control conditions | 1. Characteristics of those delivering the control |  |  |
|  | 1. Characteristics of the recipients |  |  |
|  | 1. The setting |  |  |
|  | 1. The mode of delivery |  |  |
|  | 1. The intensity |  |  |
|  | 1. The duration |  |  |
|  | 1. Adherence / fidelity to delivery protocols |  |  |
|  | 1. Detailed description of the control content provided |  |  |

### ***Supplemental Table 3: EPOC(2) Taxonomy - Implementation Strategies for category: Implementation Strategies targeted at healthcare workers***

| **Sub-Category** | **Definition** |
| --- | --- |
| Audit and feedback | A summary of health workers’ performance over a specified period of time, given to them in a written, electronic or verbal format. The summary may include recommendations for clinical action. |
| Clinical incident reporting | System for reporting critical incidents |
| Monitoring the performance  of the delivery of healthcare | Monitoring of health services by individuals or healthcare organisations, for example by comparing with an external standard. |
| Communities of practice | Groups of people with a common interest who deepen their knowledge and expertise in this area by interacting on an ongoing basis |
| Continuous quality | An iterative process to review and improve care that includes involvement of healthcare teams, analysis of a process or system, a structured process improvement method or problem-solving approach, and use of data analysis to assess changes |
| Educational games | The use of games as an educational strategy to improve standards of care. |
| Educational materials | Distribution to individuals, or groups, of educational materials to support clinical care, i.e., any intervention in which knowledge is distributed. For example this may be facilitated by the internet, learning critical appraisal skills; skills for electronic retrieval of information, diagnostic formulation; question formulation |
| Educational meetings | Courses, workshops, conferences or other educational meetings |
| Educational outreach visits, or  academic detailing | Personal visits by a trained person to health workers in their own settings, to provide information with the aim of changing practice |
| Clinical Practice Guidelines | Clinical guidelines are systematically developed statements to assist healthcare providers and patients to decide on appropriate healthcare for specific clinical circumstances (US IOM) |
| Inter-professional education | Continuing education for health professionals that involves more than one profession in joint, interactive learning |
| Local consensus processes | Formal or informal local consensus processes, for example agreeing a clinical protocol to manage a patient group, adapting a guideline for a local health  system or promoting the implementation of guidelines |
| Local opinion leaders | The identification and use of identifiable local opinion leaders to promote good clinical practice |
| Managerial supervision | Routine supervision visits by health staff |
| Patient-mediated interventions | Any intervention aimed at changing the performance of healthcare professionals through interactions with patients, or information provided by or to patients |
| Public release of performance  data | Informing the public about healthcare providers by the release of performance data in written or electronic form |
| Reminders | Manual or computerised interventions that prompt health workers to perform an action during a consultation with a patient, for example computer decision support systems |
| Routine patient-reported  outcome measures | Routine administration and reporting of patient reported outcome measures to providers and/or patients |
| Tailored interventions | Interventions to change practice that are selected based on an assessment of barriers to change, for example through interviews or surveys |

***Supplemental Table 4: Template for Intervention Description and Replication (TIDieR)(3) checklist***

|  |  | Item |
| --- | --- | --- |
| **Brief Name** | 1 | Provide the name or a phrase that describes the intervention |
| **Why** | 2 | Describe any rationale, theory, or goal of the elements essential to the intervention |
| **What** | 3 | Materials: Describe any physical or informational materials used in the intervention, including those provided to participants or used in intervention delivery or in training of intervention providers. Provide information on where the materials can be accessed (such as online appendix, URL) |
|  | 4 | Procedures: Describe each of the procedures, activities, and/or processes used in the intervention, including any enabling or support activities |
| **Who Provided** | 5 | For each category of intervention provider (such as psychologist, nursing assistant), describe their expertise, background,  and any specific training given |
| **How** | 6 | Describe the modes of delivery (such as face to face or by some other mechanism, such as internet or telephone) of the intervention and whether it was provided individually or in a group |
| **Where** | 7 | Describe the type(s) of location(s) where the intervention occurred, including any necessary infrastructure or relevant features |
| **When and How Much** | 8 | Describe the number of times the intervention was delivered and over what period of time including the number of sessions, their schedule, and their duration, intensity, or dose |
| **Tailoring** | 9 | If the intervention was planned to be personalised, titrated or adapted, then describe what, why, when, and how |
| **Modifications** | 10 | If the intervention was modified during the course of the study, describe the changes (what, why, when, and how) |
| **How well** | 11 | Planned: If intervention adherence or fidelity was assessed, describe how and by whom, and if any strategies were used to maintain or improve fidelity, describe them |
|  | 12 | Actual: If intervention adherence or fidelity was assessed, describe the extent to which the intervention was delivered as planned |

***Supplemental Table 5: List of excluded studies along with reasons for exclusion***

Full-text articles excluded, with reasons (n=72)

- Did not involve an established theoretical implementation framework, model or theory (n=31)
- Not conducted in an inpatient health service setting (n = 8)
- Did not measure changes in process of care / or yet to be published outcomes (n=17)
- Did not use a prospective study design (n = 5)
- Not published as a peer reviewed journal article (i.e. PhD dissertation, conference abstract, protocol or letter to the editor) (n = 8)
- No pre-implementation period or no comparison site data (n = 3)

| **Study** | **Reason for Exclusion** |
| --- | --- |
| Adams G, Abusaid G, Lee B, Maynard C, Campbell P, Wagner G, et al. From theory to practice: Implementation of pre-hospital electrocardiogram transmission in st-elevation myocardial infarction - a multicenter experience. *Journal of Invasive Cardiology*. 2010;22:520-525. | No theoretical implementation framework |
| Aghlmand S, Akbari F, Lameei A, Mohammad K, Small R, & Arab M. Developing evidence-based maternity care in Iran: a quality improvement study. BMC Pregnancy & Childbirth 2008;8:20. doi:10.1186/1471-2393-8-20 | No theoretical implementation framework |
| Bakhshi F, Mitchell R, Nasrabadi A N, Varaei S, & Hajimaghsoudi M. Behavioural changes in medication safety: Consequent to an action research intervention. Journal of Nursing Management 2021;29(2):152-164. | No theoretical implementation framework |
| Banerjee-Guénette P, Bigford S, & Glegg S M N. Facilitating the Implementation of Virtual Reality-Based Therapies in Pediatric Rehabilitation. Physical & Occupational Therapy in Pediatrics 2020;40(2):201-216. | Not conducted in an inpatient health service setting |
| Barakat-Johnson M, Lai M, Wand T, Coyer F, & White K. Systemwide Practice Change Program to Combat Hospital-Acquired Pressure Injuries: Translating Knowledge into Practice. Journal of Nursing Care Quality. 2020;35(1):51-57. | Did not measure changes in process of care / or yet to be published outcomes |
| Barker AL, Kamar J, Tyndall TJ, White L, Hutchinson A, Klopfer N, et al. Implementation of pressure ulcer prevention best practice recommendations in acute care: An observational study. *International Wound Journal*. 2013;10:313-320. | No theoretical implementation framework |
| Basinger M A. 2014. The reduction of central line-associated bloodstream infections in Intensive Care Units through the implementation of the comprehensive unit-based safety program. PhD. University of Nevada, Las Vegas. | PhD dissertation |
| Boonyapat S, Wongchan P, & Luppana K. Development of the Change Implementation Strategies Model Regarding Evidence-Based Chronic Wound Pain Management. Pacific Rim Int J Nurs Res 2015; 19(4) 359-372. | No theoretical implementation framework |
| Borland M. 2016. Reducing the Use of Indwelling Urinary Catheters During Cesarean Deliveries. PhD. Walden University, Minneapolis. | PhD dissertation |
| Campbell M L, Dove-Medows E, Walch J, Sanna-Gouin K, & Colomba S. The impact of a multidisciplinary educational intervention to reduce PEG tube placement in patients with terminal-stage dementia: A translation of research into practice. Journal of Palliative Medicine 01 Sep 2011;14(9):1017-1021. | No theoretical implementation framework |
| Capasso V, Collins J, Griffith C, Lasala C A, Kilroy S, Martin A T, Pedro J, & Wood S L. Outcomes of a clinical nurse specialist-initiated wound care education program: using the promoting action on research implementation in health services framework. Clinical nurse specialist CNS, 2009;23(5):252-257. | No theoretical implementation framework |
| Carter J E, Pyati S, Kanach F A, Maxwell A M W, Belden C M, Shea C M, Van De Ven T, Thompson J, Hoenig H, & Raghunathan K. Implementation of perioperative music using the consolidated framework for implementation research. Anesthesia and Analgesia 2018;127(3):623-631. | Did not measure changes in process of care as new intervention implemented |
| Cecchi F, Diverio M, Arienti C, Corbella E, Marrazzo F, Speranza G, Del Zotto E, Poggianti G, Gigliotti F, Polcaro P, Zingoni M, Antonioli D, Avila L, Barilli M, Romano E, Pellegrini L L, Gambini M, Verdesca S, Bertolucci F, Mosca I, Gemignani P, Paperini A, Castagnoli C, Hochleitner I, Luisi M, Lucidi G, Hakiki B, Gabrielli M, Fruzzetti M, Bruzzi A, Bonotti E B, Pancani S, Galeri S, Macchi C, & Aprile I. Development and implementation of a stroke rehabilitation integrated care pathway in an italian no profit institution: An observational study. European Journal of Physical and Rehabilitation Medicine 2020;56(6):713-724. | No theoretical implementation framework |
| Chang E, Hancock K, Hickman L, Glasson J, & Davidson P. Outcomes of acutely ill older hospitalized patients following implementation of tailored models of care: a repeated measures (pre- and post-intervention) design. International journal of nursing studies 2007;44(7):1079-1092. | No theoretical implementation framework |
| Colet P, Aimagambetova G, & Kossybayeva K. Evidence-based inpatient postnatal care among women in a national hospital in Kazakhstan: a best practice implementation project. International Journal of Evidence-Based Healthcare 2020;18(3):318-326.  Note: Used JBI Paces an online audit tool and a single stage (GRIP) of the JBI implementation framework | No theoretical implementation framework |
| Duffy S A, Ronis D L, Ewing L A, Waltje A H, Hall S V, Thomas P L, Olree C M, Maguire K A, Friedman L, Klotz S, Jordan N, & Landstrom G L. Implementation of the Tobacco Tactics intervention versus usual care in Trinity Health community hospitals. Implementation science: IS 04 Nov 2016;11(1):147. | No theoretical implementation framework |
| Edward K L, Walker K, & Duff J. A multi-state, multi-site, multi-sector healthcare improvement model: Implementing evidence for practice. International Journal for Quality in Health Care, 2017;29(5):740-744. | Did not measure changes in process of care / or yet to be published outcomes |
| Eskicioglu C, Pearsall E, Victor J C, Aarts M A, Okrainec A, & McLeod R S. A multifaceted knowledge translation strategy can increase compliance with guideline recommendations for mechanical bowel preparation. Journal of gastrointestinal surgery: official journal of the Society for Surgery of the Alimentary Tract, 2015;19(1):39-45. | No theoretical implementation framework |
| Finley G, Forgeron P, & Arnaout M. Action research: Developing a pediatric cancer pain program in Jordan. Journal of Pain and Symptom Management, 2008;35(4):447-454. DOI: [10.1016/j.jpainsymman.2007.05.006](https://dx.doi.org/10.1016/j.jpainsymman.2007.05.006) | No theoretical implementation framework |
| Fischler I, Riahi S, Stuckey M I, *et al.* Implementation of a clinical practice guideline for schizophrenia in a specialist mental health center: an observational study. *BMC Health Serv Res* **16,**372 (2016). <https://doi.org/10.1186/s12913-016-1618-9>. | No theoretical implementation framework |
| Gesell S B, Bushnell C D, Jones S B, Coleman S W, Levy S M, Xenakis J G, Lutz B J, Bettger J P, Freburger J, Halladay J R, Johnson A M, Kucharska-Newton A M, Mettam L H, Pastva A M, Psioda M A, Radman M D, Rosamond W D, Sissine M E, Halls J, & Duncan P W. Implementation of a billable transitional care model for stroke patients: the COMPASS study. BMC Health Services Research 2019;19(1):1-14. | Did not use a prospective study design (Evaluation using a theoretical framework) |
| Hansen P, Hammel J, Magasi S, Moore J, & Heinemann A. Innovative Knowledge Translation Strategies Used to Promote the Use of the COPM in Inpatient Stroke Rehabilitation. American Journal of Occupational Therapy 2016;70:1-1 | Conference abstract |
| Janssen P A, Holt V L, & Sugg N K. Introducing domestic violence assessment in a postpartum clinical setting. Maternal & Child Health Journal 2002;6(3):195-203. | No theoretical implementation framework |
| Jones C M, Stewart C, & Roszell S S. Beyond best practice: Implementing a unit-based CLABSI project. Journal of nursing care quality. 2015;30(1):24-30. | Did not measure changes in process of care outcomes |
| Kaiser S V, Jennings B, Rodean J, Cabana M D, Garber M D, Ralston S L, Fassl B, Quinonez R, Mendoza J C, McCulloch C E, & Parikh K. Pathways for Improving Inpatient Pediatric Asthma Care (PIPA): A Multicentre, National Study. Pediatrics 2020;145(6):1-11. | No theoretical implementation framework |
| Keller H, Koechl J M, Laur, Celia C, Helen C, Lori D J A, Gramlich L, Ray S, Valaitis R, Yang Y, & Bell J. More-2-Eat implementation demonstrates that screening, assessment and treatment of malnourished patients can be spread and sustained in acute care; a multi-site, pretest post-test time series study. Clinical Nutrition 2021;40(4):2100-2108. | No theoretical implementation framework |
| Kind AJ, Brenny-Fitzpatrick M, Leahy-Gross K, Mirr J, Chapman E, Frey B, et al. Harnessing protocolized adaptation in dissemination: Successful implementation and sustainment of the veterans affairs coordinated-transitional care program in a non-veterans affairs hospital. *Journal of the American Geriatrics Society*. 2016;64:409-416. | Not conducted in an inpatient health service setting |
| Kingsley R A. A Healthcare Improvement Initiative to Increase Multidisciplinary Pain Management Referrals for Youth with Sickle Cell Disease. Pain Management Nursing 2020;21(5):403-409. | Not conducted in an inpatient health service setting |
| Kingsnorth S, Orava T, Parker K, & Milo-Manson G. From knowledge translation theory to practice: Developing an evidence to care hub in a pediatric rehabilitation setting. Disability and Rehabilitation: An International, Multidisciplinary Journal 2020;42(6):869-879. | Did not use a prospective study design |
| Kläusler-Troxler M, Petry H, Lanter R, Naef R. Implementing family systems nursing through a participatory, circular knowledge-to-action research approach in women's health. International Practice Development Journal 2019;9(2):1-15. | Did not measure changes in process of care / or yet to be published outcomes |
| Kurrle S, Bateman C, Cumming A, Pang G, Patterson S, & Temple A. Implementation of a model of care for hospitalised older persons with cognitive impairment (the Confused Hospitalised Older Persons program) in six New South Wales hospitals. Australasian Journal on Ageing 2019;38(2):98-106.  *Note: Stated NSW agency for clinical innovation centre for healthcare redesign accelerated methodology (AIM) framework used. However, steps described do not match framework steps. Doesn’t appear that study actually used this framework*. | No theoretical implementation framework. |
| Larsen K, Hvass K E, Hansen T B, Thomsen P B, Søballe K, Larsen K, Hvass K E, Hansen T B, Thomsen P B, & Søballe K. Effectiveness of accelerated perioperative care and rehabilitation intervention compared to current intervention after hip and knee arthroplasty. A before-after trial of 247 patients with a 3-month follow-up. BMC Musculoskeletal Disorders 2008;9:59-59. | Did not measure changes in process of care outcomes |
| Lewis L. Development and evaluation of a web-based educational toolkit on the knowledge, attitudes, and practice of psychiatric prescribers regarding long-acting injectable antipsychotics. Dissertation Abstracts International: Section B: The Sciences and Engineering 2021;82(6-B). | Dissertation |
| Lin F, Marshall A P, Gillespie B, Li Y, O'Callaghan F, Morrissey S, Whitelock K, Morley N, & Chaboyer W. Evaluating the Implementation of a Multi-Component Intervention to Prevent Surgical Site Infection and Promote Evidence-Based Practice. Worldviews on Evidence Based Nursing. 2020;17(3):193-201. | No theoretical implementation framework |
| Linkewich E, Avery L, Rios J, & McEwen S E. Minimal Clinically Important Differences in Functional Independence After a Knowledge Translation Intervention in Stroke Rehabilitation. Archives of Physical Medicine and Rehabilitation 2020;101(4):587-591. | Did not measure changes in process of care / or yet to be published outcomes |
| Long-Tounsel R, Wilson J, Adams C, & Reising D L. Urban and suburban hospital system implementation of multipoint access targeted temperature management in postcardiac arrest patients. Therapeutic Hypothermia and Temperature Management 01 Mar 2014;4(1):43-50 | Did not measure changes in process of care outcomes |
| Lyon M, Sturgis L, Lottenberg R, Gibson M E, Eck J, Kutlar A, & Gibson R W. Outcomes of an Emergency Department Observation Unit-Based Pathway for the Treatment of Uncomplicated Vaso-occlusive Events in Sickle Cell Disease. Annals of Emergency Medicine September 2020;76(3 Supplement):S12-S20. | No theoretical implementation framework |
| Magee M F, Baker K M, Bardsley J K, Wesley D, & Smith K M. Diabetes to Go-Inpatient: Pragmatic Lessons Learned from Implementation of Technology-Enabled Diabetes Survival Skills Education Within Nursing Unit Workflow in an Urban, Tertiary Care Hospital. Joint Commission Journal on Quality & Patient Safety 2021;47(2):107-119. | Did not measure changes in process of care / or yet to be published outcomes |
| McCarty CA, Woehrle TA, Waring SC, Taran AM, Kitch LA. Implementation of the medfrat to promote quality care and decrease falls in community hospital emergency rooms. *JEN: Journal of Emergency Nursing*. 2018;44:280-284. | Did not measure changes in process of care / or yet to be published outcomes |
| McEwen S E, Donald M, Jutzi K, Allen K, Avery L, Dawson D R, Egan M, Dittmann K, Hunt A, Hutter J, Quant S, Rios J, & Linkewich E. Implementing a function-based cognitive strategy intervention within inter-professional stroke rehabilitation teams: Changes in provider knowledge, self-efficacy and practice. PLoS ONE 2019;14(3):e0212988. | No theoretical implementation framework |
| McLeod R S, Aarts M A, Chung F, Eskicioglu C, Forbes S S, Conn L G, et al. Development of an enhanced recovery after surgery guideline and implementation strategy based on the knowledge-to-action cycle. *Annals of Surgery*. 2015;262:1016-1025. | Did not use a prospective study design |
| Melnyk B M, Fineout‐Overholt E, Giggleman M, & Choy K. A test of the arcc© model improves implementation of evidence-based practice, healthcare culture, and patient outcomes. *Worldviews on Evidence-Based Nursing*. 2017;14:5-9. | No theoretical implementation framework |
| Moick S, Hiesmayr M, Mouhieddine M, Kiss N, Bauer P, Sulz I, Singer P, & Simon J. Reducing the knowledge to action gap in hospital nutrition care – Developing and implementing nutrition Day 2.0. Clinical Nutrition 2021;40(3):936-945. | Yet to publish outcomes |
| Mudge A M, McRae P, & Cruickshank M. Eat walk engage: an interdisciplinary collaborative model to improve care of hospitalized elders. American journal of medical quality: the official journal of the American College of Medical Quality;30(1):5-13. | No pre-implementation period data |
| O'Leary C E, Collins A, Henman M C, & King F. Introduction of a dose-banding system for parenteral chemotherapy on a haematology–oncology day ward. Journal of Oncology Pharmacy Practice: 2019;25(2):351-361. | Did not measure changes in process of care / or yet to be published outcomes |
| Opusunju E. 2017. Quality Improvement Through Evidence-Based Education: Advancing Obesity Awareness & Clinical Management Strategies for People Living with Mental Disorders. PhD. Walden University, Minneapolis. | PhD dissertation |
| Pathania S, Slater L Z, Vose C, & Navarra A M. Music Therapy and Pain Management in Patients with End-Stage Liver Disease: An Evidence-Based Practice Quality Improvement Project. Pain Management Nursing 2019;20(1):10-16. | No theoretical implementation framework |
| Rajamani A, Fernandez K, Carpen H, Liyanage U, Wang J Z, Hampton J, Oloffs A, Noel M, & Sharma A. Improving advance care planning in high‐risk hospitalised patients: a knowledge translation pilot study. Internal Medicine Journal 2021;51(4):623-624. | Letter to the editor |
| Romney W. A knowledge broker facilitated intervention to improve the use of outcome measures by physical therapists. Dissertation Abstracts International: Section B: The Sciences and Engineering 2019;80(7-B(E). | Dissertation |
| Romney W, Wormley M, Veneri D, Oberlander A, Grevelding P, Rice J, & Moore J. Knowledge translation intervention increased the use of outcome measures by physical therapists in inpatient rehabilitation. Physiotherapy Theory and Practice. 2021;DOI: 10.1080/09593985.2021.1898065 | No pre-implementation period data |
| Reese R L, Clement S A, Syeda S, Hawley C E, Gosian J S, Cai S, Jensen L L, Kind A J H, & Driver J A. Coordinated‐Transitional Care for Veterans with Heart Failure and Chronic Lung Disease. Journal of the American Geriatrics Society 2019;67(7):1502-1507. | Not conducted in an inpatient health service setting |
| Rycroft-Malone J, Seers K, Crichton N, Chandler J, Hawkes C. A, Allen C, Bullock I, & Strunin L. A pragmatic cluster randomised trial evaluating three implementation interventions. Implementation science: IS 2012;7():80. | Did not measure changes in process of care outcomes |
| Skarbo T, & Balmbra S M. Establishment of a multifamily therapy (MFT) service for young adults with a severe eating disorder-experience from 11 MFT groups, and from designing and implementing the model. Journal of Eating Disorders 2020;8:9 | No theoretical implementation framework |
| Spence K, & Henderson-Smart D. Closing the evidence-practice gap for newborn pain using clinical networks. *Journal of Paediatrics and Child Health*. 2011;47:92-98. | No theoretical implementation framework |
| Sprague A E, Oppenheimer L, McCabe L, Graham ID, & Davies BL. Knowledge to action: Implementing a guideline for second stage labor. *MCN The American Journal of Maternal/Child Nursing*. 2008;33:179-186. | No theoretical implementation framework |
| Stacey D, Vandemheen KL, Hennessey R, Gooyers T, Gaudet E, Mallick R, et al. Implementation of a cystic fibrosis lung transplant referral patient decision aid in routine clinical practice: An observational study. *Implementation Science. 10 (1) (no pagination), 2015*. 2015;Article Number:17. Date of Publication: February 07. | Not conducted in an inpatient health service setting |
| Steib A, Mertes P M, Marret E, Albaladejo P, & Fusciardi J. Compliance with guidelines for the perioperative management of vitamin k antagonists. *Thrombosis Research*. 2014;133:1056-1060. | Did not use a prospective study design |
| Stevens B J, Yamada J, Promislow S, Barwick M, Pinard M, Cihr Team in Children's P. Pain assessment and management after a knowledge translation booster intervention. *Pediatrics*. 2016;138.  *(Note: This paper was identified through manual searching reference list of an included study as paper with longer term sustainability data. Data from this study was extracted and included in final review).* | No theoretical implementation framework |
| Tangri N, Garg A X, Ferguson T W, Dixon S, Rigatto C, Allu S, Chau E, Komenda P, Naimark D, Nesrallah G E, Soroka S D, Beaulieu M, Alam A, Kim S J, Sood M M, & Manns B. Effects of a Knowledge-Translation Intervention on Early Dialysis Initiation: a Cluster Randomized Trial. Journal of the American Society of Nephrology (JASN) 2021;32. doi.org/10.1681/ASN.2020091254 | Not conducted in an inpatient health service setting |
| Tayyib N, & Coyer F. Translating Pressure Ulcer Prevention into Intensive Care Nursing Practice. Journal of nursing care quality 2017;32(1):6-14. | No pre-implementation period or no comparison site data |
| Thomas S & Mackintosh S. Use of the theoretical domains framework to develop an intervention to improve physical therapist management of the risk of falls after discharge. *Physical therapy*. 2014;94:1660-1675.  (note: This paper was identified through manual searching reference list of an included study as a development paper. Data from this study was extracted and included in final review). | Yet to be published outcomes |
| Ündar A, Wang S, Palanzo D A, Wise R, Woitas K, Baer L D, Kunselman A R, Clark J B & Myers J L. Impact of Translational Research on Optimization of Neonatal Cardiopulmonary Bypass Circuits and Techniques—The Penn State Health Approach. Artificial Organs. 2017;41: 218-223. <https://doi.org/10.1111/aor.12906> | Did not use a prospective study design |
| Van S P, Yao A L, Tang T, Kott M, Noles A, Dabai N, Coslick A, Rojhani S, Sprankle L A, & Hoyer E H. Implementing an Opioid Risk Reduction Program in the Acute Comprehensive Inpatient Rehabilitation Setting. Archives of Physical Medicine & Rehabilitation 2019;100(8):1391-1399. | No theoretical implementation framework |
| Walker M, Gay L, Raynaldo G, Von Marensdorff H, Bates J T, Friedland J A, Park J H, Kehl E, Sowers B, Bhavani S, Lan C, Bozkurt B, Stewart D E, & Horstman M J. Impact of a Resident-Centered Interprofessional Quality Improvement Intervention on Acute Care Length of Stay. Journal for Healthcare Quality: Promoting Excellence in Healthcare 2019;41(4):212-219. | No theoretical implementation framework |
| Wiechula R, Kitson A, Marcoionni D, Page T, Zeitz K, & Silverston H. (2009). Improving the fundamentals of care for older people in the acute hospital setting: Facilitating practice improvement using a Knowledge Translation Toolkit. International Journal of Evidence-Based Healthcare. | No theoretical implementation framework |
| Wilkinson S A, Hughes E, Moir J, Jobber C, & Ackerie A. Process of knowledge translation within routine clinical care: Implementing best practice in weight management. Nutrition and Dietetics 2018;75(4):363-371 | Not conducted in an inpatient health service setting |
| Williams J B, McConnell G, Allender J E, Woltz P, Kane K, Smith P K, Engelman D T, Bradford W T. One-year results from the first US-based enhanced recovery after cardiac surgery (ERAS Cardiac) program. Journal of Thoracic and Cardiovascular Surgery. 2019;(): | Did not measure changes in process of care outcomes |
| Xu Y, Li S, Zhao P, & Zhao J. Using the knowledge-to-action framework with joint arthroplasty patients to improve the quality of care transition: a quasi-experimental study. Journal of Orthopaedic Surgery & Research. 2020;15(1):1-5. | Did not measure changes in process of care / or yet to be published outcomes |
| Yasini M, Duclos C, Venot A, Lepage E, & Lamy J-B. A guideline-derived model to facilitate the implementation of test-ordering rules within a hospital information system. *Studies in Health Technology & Informatics*. 2013;192:719-723. | No theoretical implementation framework |
| Yadav K, Stahmer A, Mistry R D, May L, & Carpenter C R. An Implementation Science Approach to Antibiotic Stewardship in Emergency Departments and Urgent Care Centers. Academic Emergency Medicine 2020;27(1):31-42. | Did not measure changes in process of care / or yet to be published outcomes |
| Yorke A M, Trojanowski S, Fritz N E, Ludwa A, & Schroeder M. Standardizing Outcome Assessment in Parkinson Disease: A Knowledge Translation Project. Journal of Neurologic Physical Therapy 2021;45(1):21-27. | Not conducted in an inpatient health service setting |
| Zhou S, Dong X, Liu F, Zhang Y, Yue D, Zhou Q, Jin Y, Zheng Z J. A stepped wedge cluster randomized control trial to evaluate the implementation and effectiveness of optimized initiatives in improving quality of care for ST segment elevation myocardial infarction in response to the COVID-19 outbreak. Implementation Science 2021;16(1):38. | Protocol |

**Supplemental Table 6: Summary table of included studies**

| **Study /**  **Year** | **Study Design** | **Implementation Framework** | **Setting** | **Target Health Professionals**  **(n)** | **Target Behaviour** | **Primary Process of Care Outcome**  Classified as: Screening and assessment (Ax); Providing recommended care (Rx); or Other Process of care (Other) | **Secondary Process of Care Outcome**  Where available from category other than primary process of care (Ax, Rx or other) | **Target Patient Group**  **(n)** | **Primary Patient Outcome** | **Study Periods /**  **Data collection points** | **Sustainability data**  **(Y/N)** |
| --- | --- | --- | --- | --- | --- | --- | --- | --- | --- | --- | --- |
| **Process Models** | | | | | | | | | | | |
| Azar  et al.  2019(4) | Pre/post  (no comparison) | Agile implementation model | Multisite: 2 adult tertiary care hospitals in Indianapolis, USA | Nurses  (n=1000)  *Physicians*  *(n=>300)* | Adherence to evidenced based guidelines / bundle checklist to prevent hospital acquired central line-associated bloodstream infections (CLABSIs) | Rx - Compliance with nursing maintenance bundle checklist | N/A | In-patients with central line catheters (n=N/R) | Rate of central line associated bloodstream infections per 1000 central line days | 1.Pre-implementation  January 2015 - February 2015  Implementation  March 2016 –  June 2016  2. Post-implementation  July 2016 –  October 2017 | N |
| Cody et al. 2021(5) | Pre/post  (no comparison) | JBI evidence implementation framework | Single site: Hospital wide acute 360 bed tertiary hospital in NSW | Nurses (n = 12) | Adherence with best practice delirium recommendations to improve the quality of care provided to patients at risk of or with delirium | Rx - At risk patients offered interventions | Ax - Compliance with screening for delirium using a validated tool | Patients admitted to participating wards that were known to represent patient groups at high risk of delirium. (n=294) | Rate of hospital acquired delirium | 1.Pre-implementation  April 2019 –  June 2019  Implementation  Following the baseline audit – N/R  2.Post-implementation  October 2019 –  Nov 2019 | N |
| Gerrish et al. 2016(6) | Pre/post  (no comparison) | KTA | Single site: Three medical wards in a large hospital in England | Nutrition Champions (n = 6), RNs (n = 89), Senior Ward Nurse (n = 3), clinical nurse managers (n = 2) | Compliance with evidence-based nutrition risk screening and nutritional care for patients identified at risk of malnutrition | Ax - Assessment of patients at risk of malnutrition within 24 hours of admission | N/A | Hospitalised patients at risk of malnutrition  (n=84) | N/R | 1.Pre-implementation  N/R  Implementation  N/R  2.Post-implementation  N/R  Total duration  2011 - 2012 | N |
| Peterson et al. 2017(7) | Pre/post  (no comparison) | KTA | Multisite: 44 in-patient wards at the three hospitals in Östergötland County, Sweden. | Nurses and nurse assistants (n=approximately 2000) | Compliance with pain assessments in hospitalised patients | Ax - Pain assessments using a numerical rating scale | Rx - Patients with pain in last 24 hours who received rescue medication and who were asked if the medication alleviated the pain appropriately | Inpatients across 44 wards at 3 hospitals  (N = 2002) | N/R | 1.Pre-implementation  November 2012  Implementation  Approx.  December 2012 –  April 2013  2.6-months post-implementation  Approx. May 2013  3.12-months post-implementation  Approx. November 2013  Total duration  2012 - 2013 | Y  12-months post-implementation |
| Rattray et al. 2021(8)  &  Rattray et al. 2021(9) | Pre/post  (no comparison) | KTA | Single site: 1 gastrointestinal ward at a large tertiary teaching hospital in Queensland.  *? Gold Coast university hospital*  *HREC – Gold Coast Health* | Doctors, nurses, nursing assistants, dietitians  (n=N/R) | Improving post-operative nutrition care practices and intake after colorectal surgery | Other - Time (in hours) to first prescribed diet | Rx - Time (in hours) to first diet delivery | Adults undergoing elective colorectal and/or small bowl surgical procedure  (n=64) | Nutrition (any type) intake ≤ 6 hours  (Proportions) | 1.Pre-implementation  June 2017 –  August 2017  Implementation  February 2018 –  June 2018  2.Post-implementation  August 2018 - October 2018 | N |
| Reynolds t al.  2019(10) | Pre/post  (no comparison) | Grol and Wensing Model of Implementation | Single site: Neuro ICU in a large tertiary care centre, south eastern USA | Neurological ICU nursing staff  (n=85) | Adherence to evidenced-based central line catheter protocol | Rx - Compliance with daily bathing of central line catheter with chlorhexidine gluconate | N/A | Neurological ICU patients with a central catheter  (n=N/R) | Rate of hospital acquired CLABSIs per 1000 catheter days | Pre-implementation  August 2016 – October 2016  Implementation November 2016 – December 2017  Post-implementation  January 2017 -  August 2017  1.Weekly documentation audits  August 2016 -  August 2017 | N |
| Salbach et al. 2017(11)  &  Munce et al. 2017(12) | Cluster-randomized trial | KTA | Multisite: 20 rehabilitation units in Canada | Nurses, Occupational Therapists and Physiotherapists  (n=N/R*)  *Reported mean number at each site not totals | Compliance with stroke rehabilitation guidelines in inpatient stroke rehabilitation | Rx -Adherence with guidelines regarding treating sit to stand | N/A | Adult stroke patients receiving in-patient rehabilitation (n=312) | N/R | 1.Pre-implementation  N/R  Implementation  N/R  (16-month period)  2.Post-implementation  N/R  Total duration  2007 - 2009 | N |
| Semin-Goossens et al.  2003(13) | Pre/post  (no comparison) | Grol’s 5-step implementation model | Single site: 2 wards in a large, academic teaching hospital in Amsterdam, Netherlands | Nurses working on neurology or internal medicine ward  (n=67) | Adherence with evidenced based falls prevention nursing guideline | Other -  Compliance with completing falls incident report form  (Internal medicine ward) | N/A | Inpatients on a neurology or internal medicine ward  (n=2,670) | Rate of falls (internal medicine ward) per 1000 bed days | 1.Pre-implementation  Oct –Nov 1999*  Implementation  Dec 1999 – Dec 2000  2.Post implementation  Jan – July 2001 | N |
| White et al. 2020(14) | Pre/post  (no comparison) | KTA | Multisite: 25 surgical hospitals across Cameroon | All Surgical staff  Surgeons, anaesthesia providers, nurses and other (biomedical technicians, sterile processing technicians, medial or nursing students)  (n=425 total) | Compliance with the WHO surgical safety checklist | Rx - Compliance with using a surgical safety checklist | Ax - Adherence to risk assessment for difficult intubation | N/R  (n=N/R) | N/R | 1.Pre-implementation  July 2016–July 2017  Implementation  August 2017–June 2018  2.4 months post training  March 2018–June 2018 | N |
| **Determinant Frameworks** | | | | | | | | | | | |
| Byrnes  et al.  2018(15) | Pre/post with concurrent comparison ward | Integrated Promoting Action on Research Implementation in Health Services  (iPARIHS) | Single site: 2 general surgical wards at a metropolitan tertiary hospital in Australia | Multidisciplinary team (including dietitians, surgeons and nurses)  (N/R) | Adherence to evidence-based postoperative diet guidelines | Rx - Upgrade to full ward diet  (or appropriate texture modified diet) day of surgery of post-op day 1 | N/A | General surgical patients ≤65 years age  (n=155) | Proportion of patients that receive early nutrition | 1.Pre-implementation  September 2015 – December 2016  Implementation  April 2017 – N/R  2.Post-implementation  December 2017 – March 2018 | N |
| Garrido et al. 2021(16) | Pre/post  (no comparison) | CFIR | Single site: 2 units  Intermediary care unit & Internal medicine unit that care for older patients with acute pathology without mechanical ventilation in Chile | Medial, nursing, physical therapists, and nurse assistants  (n = 149) | Adherence with non-pharmacological delirium prevention program | Rx - Global adherence with 12 indicators of non-pharmacological delirium prevention | N/A | Elderly patients >65 years at risk of delirium (n=72) | Incidence of delirium measured with validated tool | 1.Pre-implementation  N/R  2.Post-Training  N/R  3.6 months post implementation  N/R  Overall study duration  March 2017 –  October 2018 | Y  6 months post implementation |
| Gu et al. 2020(17) | Pre/post  (no comparison) | iPARiHS | Single site: Cardiac centre at Children’s Hospital of Fudan University, Shanghai, China | Nurses (n=100) | Compliance with evidence-based nutrition risk screening and assessment guidelines | Ax - Nutritional assessment among infants with moderate risk and above | Rx - Summary and intervention plan developed after nutritional assessment | Infants with congenital heart disease (n=142) | Time to initiation of enteral nutrition | 1.Pre-implementation  September 2016  Implementation  October 2016 –  January 2017  2.Post-implementation  February 2017 –  March 2017 | N |
| Peel et al. 2021(18) | Pre/post  (no comparison) | iPARiHS | Single site: 200 bed acute care hospital in Brisbane. (all of acute care) | Nurses  (n=124) | Compliance with administering an integrated tool to screen for functional and psychological risks of harm in acute care | Ax - Proportion of screening items complete in routine screening documentation | N/A | All acute care admissions patients aged > 18 years.  (n=690) | N/R | 1.Pre-implementation  N/R  Implementation  N/R  2.Post-implementation  N/R  Total duration  2017 – 2018 | N |
| Savoie et al. 2019(19) | Pre/post  (no comparison) | Spinal Cord Injury Knowledge Mobilisation Network (SCI KMN) Implementation approach based on the Active Implementation Frameworks of the National Implementation Research Network (NIRN) | Single Site: Neuro-rehabilitation unit in Canada | Nursing, OT, Physiatry, Physiotherapy and Psychology  (n=N/R) | Compliance with documentation of evidence-based pain assessment and management in SCI | Ax - Assessment of pain on admission using ISCIPBDS 2.0 | Rx - Development of an Inter-professional pain treatment plan | Inpatients with SCI > 15 years age, admitted anticipated period >14 days  (n=70) | N/R | 1.Pre-implementation  N/R  2.Post-implementation  N/R  Total duration  2015 – N/R | N |
| Scovil  et al.  2019(20) | Pre/post  (no comparison) | “Active Implementation Frameworks” of the National Implementation Research Network (NIRN) | Multisite: 6 Spinal cord injury (SCI) rehabilitation centres across Canada | Multidisciplinary teams working in SCI inpatient rehabilitation  (N/R) | Adherence with clinical practice guidelines for pressure injury prevention in spinal cord injury (SCI) rehabilitation | Ax - Completion rates of inter-professional pressure injury risk factor determination | Rx - Delivery of educational materials | SCI rehabilitation inpatients  (n=2,371) | Pressure injury incidence (any stage) | 1.Pre-implementation  2012 – 2013  Implementation  Slightly different time points for each site between 2012-2013  2.Post implementation  2014 - 2015 | N |
| Stevens  et al.  2014(21)  &  Stevens et al. 2016(22) | Pre/post with concurrent comparison ward | Promoting Action on Research Implementation in Health Services (PARiHS) | Multisite: 32 units (10 critical care, 8 surgical and 14 medical units) from 8 paediatric hospitals in Canada | Paediatric clinicians  (N/R) | Adherence with evidenced based pain assessment and management in hospitalized children | Ax - Pain was assessed with any pain assessment | Rx - Children per unit who underwent any painful procedure with any pain management strategy | Hospitalised children  (n=2,564) | Pain intensity - mild, moderate or severe | 1.Pre-implementation  N/R  2.Post implementation  N/R  3. 6 months post implementation (pain intensity)  N/R  4. 24 months post-implementation  N/R  5. 36 months post-implementation  N/R  6. 48 months post-implementation  N/R  Total duration  2008 - 2013 | Y  24 months,  36 months &  48 months  Post-implementation |
| Sving  et al.  2016(23)  &  Sving et al. 2020(24) | Clustered pre/post (no comparison) | PARiHS | Single site: Five units (three surgical and two medical units) in a 344-bed general hospital in Sweden | Registered nurses  (n=169+N/R data collection point 3)  Assistant nurses  (n=39+N/R data collection point 3)  Multidisciplinary team (physiotherapists, Occupational Therapists and dietitians)  (N/R) | Adherence with evidenced-based pressure ulcer prevention | Rx - Patients for whom prevention measures were implemented | Ax - Skin assessments documented within 24 hours of admission | Inpatients on surgical or medical units  (n=763) | Prevalence of categories pressure Ulcers 1–4 all patients | 1.Pre-implementation  N/R  2.6-8 months post implementation  N/R  3.36-42 months post implementation  N/R  Total duration  Jan 2012 - June 2013 | Y |
| Taylor et al. 2013(25)  &  Taylor et al. 2014(26) | Pre/post  (no comparison) | TDF embedded within a stepped implementation approach developed by study authors and defined as TDF Implementation (TDFI) approach. | Multisite: Three UK hospitals | Multidisciplinary clinicians involved in the target behaviour (ie doctors, nurses and dieticians)  (n = 227) | Adherence with guidelines relating to checking correct placement of nasogastric feeding tubes | Re-Ax - Use of pH as first line check of nasogastric tube placement | N/A | Patients who had a nasogastric tube inserted during an inpatient admission (n=244) | N/R | 1.Pre-implementation  Jan – Sept 2011  Implementation  Approx. Sept 2011  2.Post implementation  Oct 2011 –  June 2012 | N |
| Thomas et al. 2014(27)  &  Thomas et al. 2016(28) | Pre/post  (no comparison) | TDF  (embedded within a 4-step systematic method for developing an implementation intervention(29)) | Single site: Flinders Medical Centre (FMC), a 588-bed acute teaching hospital located in South Australia. | Physiotherapists  (n=N/R) | Improve physical therapists’ adherence to key guideline recommendations for managing risk of falls on discharge from one hospital | Ax - Patients identified as being at risk of falls prior to discharge | Other - Documentation of clinical handover at discharge | Admitted patients identified as being a falls risk on hospital discharge  (n=313) | N/R | 1.Pre-implementation  March 2012 -February 2013  Implementation  N/R  2.Post implementation  N/R  (6 weeks - 12 months following implementation) | N |
| Tian  et al.  2017(30) | Pre/post  (no comparison) | PARIHS | Single site: Departments of medical oncology and radiotherapy in an adult university-affiliated hospital in Suzhou, China | Nurses  (n=39) | Adherence with Clinical Practice Guideline: Nursing Care of Cancer-Related Fatigue in Adults with Cancer | Rx - Compliance with the evidence-based practice (EBP) using a "nursing quality checklist of inpatient Cancer Related Fatigue management" | N/A | Adult inpatients in departments of medical oncology or radiotherapy  (n=204) | Cancer Related Fatigue self-management ability relating to “knowledge” | 1.Pre-implementation  May - July 2015  Implementation  July - September 2015  2.Post implementation  N/R | N |
| White et al. 2019(31) | Pre/post  (no comparison) | CFIR | Multisite: 36 hospitals that represented the majority of government surgical hospitals in Benin | Surgical Team including surgeons, anaesthetists, nurses, and any other perioperative staff  (n=total 821 across data points) | Compliance with perioperative surgical safety checklist | Rx - Compliance with using a surgical safety checklist | Ax - Adherence to risk assessment for difficult intubation | Emergency and elective surgery patients  (n=N/R) | N/R | 1.Pre-implementation  January - August 2016  Implementation &  2.4 months post training  September 2016 - May 2017  3.12-18 months post training  April – May 2018 | N |
| **Combination > 1 framework types** | | | | | | | | | | | |
| Bosch et al. 2014(32)  &  Tavender et al. 2015(33)  &  Bosch et al. 2019(34) | Cluster randomised trial | Theoretical Domains Framework (TDF)  & Model of Diffusion of Innovations in Service Organisations  used in a complementary manner | Multisite: Australian EDs  14 intervention & 17 control | Nursing & Medical  (n= N/R) | Adherence with evidence-based guidelines for managing mild traumatic brain injury in the ED | Ax - Appropriate post-traumatic amnesia screening using a valid tool | Rx –  Provision of written information at discharge | Patients who present to ED with mild head injuries  (n=1943) | Anxiety measured with  Hospital Anxiety and depression (HADS) scale | 1. Pre-implementation  Nov 2010 –  Oct 2013  Implementation  Mid 2014  1.Post implementation  2-month period after the last intervention component  (ranging between October 2014 and February 2015) | N |
| Roberts et al. 2019(35)  &  Roberts et al. 2019(36) | Pre/post  (no comparison) | TDF  (embedded within a 4-step systematic method for developing an implementation intervention(29)) &  Knowledge to action (KTA) | Single unit: Acute medical unit public hospital in Southeast Queensland. | Nurses, doctors, food service staff  (n=N/R) | Improving nutritional care, delivery and intakes among hospitalised patients. | Rx - Patient ready to receive their meal tray when meal tray delivered | N/A | Adults >18 years admitted to a general medical ward.  (n=207) | Adequate energy intake | 1.Pre-implementation  February 2015 –  March 2015  Implementation  N/R  2.Post-implementation  March 2016 –  April 2016 | N |
| Robertson et al.  2018(37) | Pre/post  (no comparison) | Knowledge to action (KTA)  & TDF (embedded within KTA cycle) | Single unit: Colorectal ward in a large tertiary metropolitan teaching hospital in Brisbane, Australia | Colorectal consultants, surgical trainees, junior medical doctors and nursing staff  (N/R) | Adherence with  early oral feeding post-colorectal surgery guideline | Rx - Proportion prescribed a full diet on operative notes | N/A | Adult patients who underwent an elective bowel resection or stoma reversal  (n = 130) | Proportion who received a full diet on post op. day 0 | 1&2. Pre-implementation  June – July 2016 &  Feb – March 2017  3&4. Implementation  June-July 2017 &  October–Nov 2017  5. Post-implementation  Jan – Feb 2018 | N |
| Romney et al 2019(38)  &  Romney et al. 2020(39) | Pre/post  (no comparison) | TDF &  KTA | Single unit: Independently operated inpatient subacute unit in Connecticut, USA | Physical Therapists  (n=11) | Compliance with using a gait speed outcome measure as part of routine initial and discharge assessment | Ax – Assessment of gait speed (4MWT) at Initial examination | N/A | Adults with mixed diagnosis receiving inpatient rehabilitation  (n=162) | N/R | 2 (5 in total in study – data to report from 2)  1.Pre-implementaion  N/R  (0-4 weeks intervention design)  Implementation  (Week 4-8 Implementation period)  2.8-months post implementation  N/R  (Month 4 -9 follow up period) | N |

N/R: Not reported; ICU: Intensive care unit; USA: United States of America; *Data from earlier 1993 project used for pre-implementation data relating to completion of falls incident reports N/R: Not reported, USA: United States of America.

***Supplemental Table 7: TIDieR Table***

| **Study /**  **Year** | **Brief Name** | **Why** | | **What materials** | | **What procedures** | | **Who provided** | | **How provided** | | **Where (setting)** | | **When & how much (dose)** | | **Tailoring (Y/N)** | | **Source of evidence / references** | |
| --- | --- | --- | --- | --- | --- | --- | --- | --- | --- | --- | --- | --- | --- | --- | --- | --- | --- | --- | --- |
| Azar  et al.  2019(4) | Evidence-based bundle to manage central lines | Improve infection control procedures to reduce central line associated blood steam infections (CLABSI) | | Guideline  Maintenance bundle checklist | | Guidelines specified indications for central line: (i) placement; (ii) removal; (iii) duration; and (iv) the type of line to be used  Maintenance bundle checklist included: (i) specifications for changing tubing (regular intravenous tubing; total parenteral nutrition; propofol); (ii) changing dressing (transparent dressing with or without chlorhexidine gluconate; transparent dressing with gauze); and (iii) ensuring that the dressing is clean, dry, and occlusive | | Nurses  Physicians (including first-year residents and fellows who were expected to insert central lines) | | Face to face at point of care delivery | | Inpatient setting at two adult tertiary care hospitals in Indianapolis, United States of America | | Maintenance bundle checklist included frequency: changing tubing (regular intravenous tubing: every 96 hours; total parenteral nutrition: every 24 hours; propofol: every 12 hours); changing dressing (transparent dressing with or without chlorhexidine gluconate: every 7 days; transparent dressing with gauze: every 48 hours) | | Yes  Guideline and bundle checklist were adapted to the local context by a group of local opinion leaders | | National (United States Centre for Disease control and prevention) guidelines^1^ and expert guidance document supported by a number of national (United States) professional associations^2^ | |
| References | 1. O’Grady NP, Alexander M, Burns LA, Dellinger EP, Garland J, Heard SO, et al. Guidelines for the prevention of intravascular catheter-related infections. Am J Infect Control 2011;39(4 Suppl 1):1-34. 2. Marschall J, Mermel LA, Fakih M, Hadaway L, Kallen A, O'Grady NP, et al. Strategies to prevent central line-associated bloodstream infections in acute care hospitals: 2014 update. Infect Control Hosp Epidemiol 2014;35:753-71. | | | | | | | | | | | | | | | | | | |
| Bosch et al. 2019 | Evidenced based management of mild traumatic brain injury (mTBI) in the Emergency Department (ED) | Provide evidenced based management of mTBI in an emergency department context including the uptake of three key clinical practice recommendations | | Clinical Practice Guideline  Electronic & paper version of the validated amnesia screening tool (A-WPTAS)  Relevant materials (e.g.  CT-head rules for when a scan is indicated)  Consumer information booklet in English, Greek, Italian, Arabic, Chinese & Vietnamese to provide written information at discharge | | Screening for amnesia using a validated tool until perfect score achieved  Appropriate CT scanning (as per guideline)  Provision of written information for patients at discharge  Documentation of management of mTBI in the ED | | Nurses  Medical staff | | Face to face at point of care in ED | | 31 24-hour Emergency departments across Australia | | Screening at least four hourly, until a perfect amnesia screening score achieved | | Yes  Intervention tailored to local processes | | Published literature review conducted by study team^1^ | |
| Reference | 1. Tavender EJ, Bosch M, Green S, O'Connor D, Pitt V, Phillips K, Bragge P, Gruen RL. Quality and consistency of guidelines for the management of mild traumatic brain injury in the emergency department. Acad Emerg Med. 2011 Aug;18(8):880-9. doi: 10.1111/j.1553-2712.2011.01134.x. PMID: 21843224. | | | | | | | | | | | | | | | | | | |
| Byrnes et al. 2018 | Adherence with early postoperative diet upgrade guidelines (consistent with Enhanced Recovery After Surgery, ERAS) in older surgical patients | To enhance recovery of older patients after surgery | | Guideline | | Limited details of exactly what was in guideline or specifics of what processes involved  Prescribing diet upgrades    Delivery of diet upgrades    Availability of food in hospital context | | Dietitians  Nurses  Surgical staff (junior and senior consultants) | | N/R  Unsure if diet upgrades electronic or paper based  Provision of food to an inpatient | | Across two general surgical wards at a metropolitan tertiary teaching hospital in Australia. | | Once  Yes/no diet upgrades delivered day of surgery or post-operative day one | | Yes  Diet texture could be modified if clinically indicated or initiation of enteral or parenteral nutrition as indicated | | *Review papers^1,2^* | |
| References | 1. Ljungqvist O, Scott M, Fearon KC. Enhanced recovery after surgery: a review. JAMA Surg 2017; 152: 292–8. 2. Gianotti L, Beretta S, Luperto M, Bernasconi D, Valsecchi MG, Braga M. Enhanced recovery strategies in colorectal surgery: is the compliance with the whole program required to achieve the target? Int J Colorectal Dis 2014; 29: 329–41. | | | | | | | | | | | | | | | | | | |
| Cody et al.  2021 | Best practice recommendations relating to screening, assessment, prevention and management of hospitalised patients at risk of, or with, delirium | To ensure that patients at risk of delirium are identified early and receive preventative strategies, and that those with delirium receive optimal treatment to address their condition | | Locally developed and tested screening and assessment tool | | Delirium screening & assessment (if indicated following screening)  Screening for falls and pressure injuries  Carers provided with information and strategies to manage delirium  Interventions to prevent and manage delirium offered  Non-pharmacological management (first line management) for distressed patients with delirium | | Nurses  Occupational Therapists  Junior medical staff | | Face to face at point of care during inpatient hospital care | | Hospital wide at 360-bed metropolitan tertiary hospital in New South Wales (NSW), Australia | | During entire Inpatient admission | | Yes  Sunflower tool to individualise person centred care  Cause of distress investigated  Develop an individualised care plan | | The Australian Commission on Safety and Quality in Health Care (ACSQHC) Delirium Clinical Care Standard^1^ and recommendations from a number of delirium clinical guidelines and review papers.^2-8^ | |
| References | 1. Australian Commission on Safety and Quality in Health Care. Delirium clinical care standard. ACSQHC. Sydney. 2016 2. National Institute for Health and Care Excellence. Delirium: prevention, diagnosis and management – clinical guideline [CG103]. 2019. Available from: <https://www.nice.org.uk/guidance/cg103> 3. Devlin J, Skrobik Y, Gelinas C, Nedham D, Slooter A, Pandharipande P, et al. Clinical practice guidelines for the prevention and management of pain, agitation/sedation, delirium, immobility, and sleep disruption in adult patients in the ICU. Crit Care Med. 2018; 46(9): e825-e873. 4. Bush SH, Lawlor PG, Ryan K, Centeno C, Lucchesi M, Kanji S, et al. Delirium in adult cancer patients: ESMO Clinical Practice Guidelines. Ann Oncol. 2018; 29(Suppl 4): iv143-iv165. 5. American Geriatrics Society Expert Panel on Postoperative Delirium in Older Adults. American Geriatrics Society abstracted clinical practice guideline for postoperative delirium in older adults. J Am Geriatr Soc. 2015; 63(1): 142-50. 6. Marin T. Evidence Summary. Postoperative Delirium (Older People): Prevention and Management. The Joanna Briggs Institute EBP Database, JBI@Ovid. 2019; JBI3727. 7. Slade S. Evidence Summary. Delirium in Critical Care Settings: Screening and Assessment. The Joanna Briggs Institute EBP Database, JBI@Ovid. 2020; JBI5419. 8. Marin T. Evidence Summary. Postoperative Delirium (Older People): Prevention and Management. The Joanna Briggs Institute EBP Database, JBI@Ovid. 2019; JBI3727. | | | | | | | | | | | | | | | | | | |
| Garrido et al. 2021 | Non-pharmacological delirium prevention program | Provide evidenced based care in relation to preventing delirium using a  nonpharmacological multicomponent intervention | | Calendar & Clock in patients’ room  Delirium brochure  Early mobilisation protocol  Protocol non-pharmacological interventions to prevent delirium (nPPD)  Staff training package (not available online or in article) | | Care provided across the following domains (additional detail provided in paper):  Orientation  Environmental management  Early mobilisation  Correction of sensory impairments  Sleep protocols  Hydration correct Family participation  Drug reduction | | Multidisciplinary teams | | Face to face at point of care during inpatient hospital care. | | Two medical inpatient wards in Chile | | Duration of admission to intervention units | | Yes  Interventions were broad categories of care provision that could be tailored | | NICE guidelines^1^ and national (US) professional association clinical guidelines.^2^ | |
|  | 1. Young J, Murthy L, Westby M, Akunne A, O'Mahony R, Group GD. Diagnosis, prevention, and management of delirium: summary of NICE guidance. BMJ. 2010;341:c3704. 2. The American Geriatrics Society Expert Panel on Postoperative Delirium in Older Adults. American Geriatrics Society abstracted clinical practice guideline for postoperative delirium in older adults. J Am Geriatr Soc, 2015;63(1):142‐150. | | | | | | | | | | | | | | | | | | |
| Gerrish et al. 2016 | Best practice malnutrition screening and nutrition care provision for hospitalised patients | Provide evidenced based care in relation to  malnutrition screening and recommendations for achieving evidenced based nutrition to hospital patients | | Malnutrition Universal Screening Tool (MUST)  Nutrition screening workbook (one ward only)  Patient specific communication sheet at bedside | | Screening using MUST tool  MUST assessment on admission  Improved mealtime experience  for patients  Enhanced communication  relating to patients’ nutritional  needs include risk assessment,  food preferences, dietary  requirements, specialist equipment, and  physical assistance  Improved presentation of food and portion size tailored to  patient preference | | Nurses  Dietitians  Catering staff | | Face to face at point of care during inpatient hospital care | | Three medical wards in an inpatient medical ward at a Hospital in England | | Screening once in the first 24 hours of admission  Mealtimes for duration of inpatient admission on medical ward | | No - Screening tool standardised  Yes - Mealtime experience could be personalised | | National (UK) professional association recommendations_1,2_ | |
| References | 1. British Association of Parental and Enteral Nutrition (2003) The ‘MUST’ Explanatory Booklet: A Guide to the Malnutrition Universal Screening Tool (MUST) for Adults. BAPEN, Redditch. 2. British Association of Parental and Enteral Nutrition (2012) Combatting Malnutrition: Recommendations for Action. BAPEN, Redditch. | | | | | | | | | | | | | | | | | | |
| Gu et al. 2020 | Completing evidenced based nutrition risk screening and assessment for infants with congenital heart disease (CHD) | | Provide evidence-based care in relation to screening for malnutrition in infants and children with CHD and providing  timely nutritional support based on the nutritional status and needs of children | | Clinical guideline  Screening tool | | Nutrition screening  Nutrition assessment  Providing  timely nutritional support based on the nutritional status and needs of children | | Nurses | | Face to face at point of care during inpatient hospital care | | Inpatient infant cardiac units in China | | At admission and then throughout the inpatient admission | | No | | National (US) professional association clinical guidelines^1,2^ |
| References | 1. Mehta NM, Compher C; A.S.P.E.N. Board of Directors: American Society of parenteral and enteral nutrition (A.S.P.E.N.) Clinical Guidelines: Nutrition support of the critically ill child. JPEN J Parenter Enteral Nutr 2009; 33:260–276. 2. Mehta NM, Skillman HE, Irving SY, et al: Guidelines for the provision and assessment of nutrition support therapy in the pediatric critically ill patient: Society of Critical Care Medicine and American Society for Parenteral and Enteral Nutrition. Pediatr Crit Care Med 2017; 18:675–715. | | | | | | | | | | | | | | | | | | |
| Peel et al 2021 | Use of the interRAI Acute Care (AC), a nurse-administered standardized assessment of functional and psychosocial domains for patients admitted to acute care | | To identify cognitive, behavioural, mental, physical, social and other circumstances early in an inpatient admission to assist in care planning | | Electronic training module  InterRAI Acute Care assessment manual  Hard and electronic copies of a toolkit | | Electronic screening tool (separate to electronic medical record) | | Nurses | | Face to face screening and assessment at point of care during inpatient hospital care | | Hospital setting in Brisbane, Australia across a number of wards | | Once at admission  Once at discharge | | No | | Unknown |
| References | No references provided for the source of evidence for this intervention | | | | | | | | | | | | | | | | | | |
| Peterson et al 2017 | Completion and documentation of systematic pain assessment in an inpatient context | | Provide evidenced based care in relation to pain assessments in inpatient hospital setting | | Numeric rating scale consisting of a visual or imagined 11-point scale with the endpoints 0 (no pain) and 10 (worst pain imaginable)  Documentation in electronic medical record | | Completing and documenting pain assessments in medical records using a Numeric Rating Scale (NRS) | | Nurses  Nursing assistants | | Face to face assessment at point of care during inpatient hospital care | | 44 in-patient wards across three hospitals in Sweden | | Pain assessment using a numeric rating scale should be performed at least once per work shift (i.e., three times per 24 hours), as well as before and after administering ‘rescue’ medication for transient pain | | Yes  The new pain assessment routines were adapted to the local context | | Descriptive study exploring consumer preferences^1^ |
| References | 1. Yazici Sayin Y, Akyolcu N. Comparison of pain scale preferences and pain intensity according to pain scales among Turkish Patients: a descriptive study. Pain Manag Nurs, 2014;15:156–64. | | | | | | | | | | | | | | | | | | |
| Rattray et al. 2021 | Reintroduction of liquid and solid foods within 24 hours after colorectal surgery in noncritically ill adult patients | | Provide evidenced based care in relation to postoperative nutrition care practices and intake in colorectal patients | | Oral nutrition supplement (ONS)  Free fluids  High-energy, high-protein (HEHP) diet  Electronic foodservice system  Nutrition education handout | | Prescribing and delivering free fluids and solid foods  Patients being able to order meals | | Doctors (Interns, residents and registrars)  Nurses | | Diet prescription  Nursing staff entered patients’ dietary prescriptions into the electronic foodservice system and patients ordered their main meals via a bedside patient entertainment system screen (> 3 h in advance of meal delivery)  Food delivery direct to inpatients in hospital | | Gastrointestinal ward at  a large tertiary teaching hospital in Queensland, Australia | | One oral nutrition supplement (ONS) on postoperative day (POD) 0;  Free fluids prescribed on POD0;  High-energy, high-protein (HEHP) diet prescribed on POD1;  ONS prescribed three times a day (TDS) from POD1 until discharge;  Education on meal ordering on or after POD1;  Nutrition-related messages delivered by surgeons during ward rounds on  POD1 and thereafter; and  a nutrition education handout given before surgery | | Yes  Early oral feeding pathway was flexible and individualised, encouraging healthcare professionals to exercise their clinical judgement and refer nutritionally at-risk patients to a dietitian | | International evidenced based guidelines^1,2^ and national (US) professional association consensus statement^3^ |
| References | 1. Nygren J, Thacker J, Carli F, Fearon KCH, Norderval S, Lobo DN, et al. Guidelines for Perioperative Care in Elective Rectal/Pelvic Surgery: Enhanced Recovery After Surgery (ERAS) Society Recommendations. World J Surg 2013;37(2):285–305. 2. Gustafsson UO, Scott MJ, Schwenk W, Demartines N, Roulin D, Francis N, et al. Guidelines for perioperative care in elective colonic surgery: Enhanced Recovery After Surgery (ERAS) Society recommendations. World J Surg 2013;37(2):259–84. 3. Wischmeyer P, Carli F, Evans D, Guilbert S, Kozar R, Pryor A, et al. American Society for Enhanced Recovery and Perioperative Quality Initiative Joint Consensus Statement on Nutrition Screening and Therapy Within a Surgical Enhanced Recovery Pathway. Anesth Analg 2018;126(6):1883–95. | | | | | | | | | | | | | | | | | | |
| Reynolds et al. 2019 | Daily bathing using chlorhexidine gluconate (CHG) cloths to decrease preventable hospital-acquired central line–associated bloodstream infections (CLABSIs) | | Provide evidenced based care to decrease CLABSIs | | Chlorhexidine gluconate  cloths | | Daily bathing using chlorhexidine gluconate (CHG) cloths | | Intensive care unit (ICU) nurses | | Face to face at point of care during inpatient hospital care | | Neuro ICU in a  large, tertiary hospital in the USA | | Daily during ICU admission for period that patient had a central line catheter | | No | | A national (United States) protocol for central line infection prevention developed by the Agency for Healthcare Research and Quality (AHRQ)^1^ |
| References | 1. Agency for Healthcare Research and Quality. Universal ICU Decolonization: An Enhanced Protocol. Rockville, MD: Agency for Healthcare Research and Quality; 2013. | | | | | | | | | | | | | | | | | | |
| Roberts et al. 2019 | Improve nutrition care, delivery, and intake among acute medical inpatients | | Provide best practice care in relation to preventing malnutrition in medical inpatients | | White board with magnets  Hospital foodservice system | | Moved breakfast to earlier time  Added hot breakfast option  Reminder board displaying patient’s nutrition status (traffic light) | | Nurses  Medical staff  Foodservice providers | | Nutrition provided at point of care during inpatient hospital care | | Acute medical ward in Queensland, Australia | | Three meals a day during the entire admission on acute medical unit | | Yes  Patients could choose hot breakfast from 4 options | | n/a |
| References | No specific evidence sources.  General evidence that nutrition is important and referenced publications concerning some specific nutrition interventions. | | | | | | | | | | | | | | | | | | |
| Robertson et al. 2018 | Early oral feeding after colorectal surgery | | Evidence-based guidelines recommend early oral feeding (EOF) prescribed as an unrestricted diet within 24 hours after colorectal surgery | | Integrated electronic medical records  Electronic foodservice management system  Post-op orders template | | Electronic prescription of post-operative fluids and diet orders via integrated electronic medical records and an electronic foodservice management system  Delivery of fluids and food | | Medical staff  Nurses  Foodservice staff | | Delivery of fluids and food at the point of care delivery for hospital inpatients  Electronic prescription of post-operative fluids and diet orders | | Colorectal ward in a large tertiary metropolitan teaching hospital in Brisbane, Australia | | Patients followed until a full diet was commenced.  This included: (i) the initial diet prescribed by the surgeon in the operation notes; (ii) the diet received on the ward on postoperative Day 0; (iii) the postoperative day the patient was commenced on a free fluid diet; and (iv) the postoperative day the patient was commenced on a full (i.e. unrestrictive) diet. | | Yes  Tailoring based on clinical assessment | | International  evidence-based guidelines^1,2,3^ |
| References | 1. Weimann A, Braga M, Carli F et al. ESPEN guideline: clinical nutrition in surgery. Clin Nutr 2017; 36: 623–50. 2. Gustafsson UO, Scott MJ, Schwenk W et al. Guidelines for perioperative care in elective colonic surgery enhanced recovery after surgery (ERAS) society recommendations. World J Surg 2013; 37: 259–84. 3. Carmichael JC, Keller DS, Baldini G. Clinical practice guidelines for enhanced recovery after colon and rectal surgery from the American Society of Colon and Rectal Surgeons Society of American Gastrointestinal and Endoscopic Surgeons. Dis Colon Rectum 2017; 60: 761–84. | | | | | | | | | | | | | | | | | | |
| Romney et al. 2020 | Physical therapists’ use of a selected outcome measure (The 4-m walk test) in an inpatient sub-acute rehabilitation hospital | | Recommended practice to include  standardized outcome measures to determine plan of care, document patient progress, and communicate with providers, patients, and insurers | | Documentation changes to capture 4-meter walk assessment  4-Meter walk track marked out  Stopwatches | | 4-Meter walk test included as part of routine physical therapy initial examination and discharge for all patients where appropriate | | Physical Therapists | | Face to face as part of routine clinical assessments | | Private rehabilitation hospital with a Physical Therapy department in Connecticut, USA | | Once at initial assessment and once at discharge assessment | | Yes  Only included where it was clinically appropriate | | National (US) professional association recommendation^1^ |
| References | 1. Field-Fote, E. 2015. Towards optimal practice: What can we gain from assessment of patient progress with standardized outcome measures. Section on Research, American Physical Therapy Association <http://www.ptresearch.org/article/104/resources/researchers/edge-task-force-evaluation-database-to-guide-effectiveness>. | | | | | | | | | | | | | | | | | | |
| Salbach et al. 2017 | Recommended treatments for evidenced based stroke care | | Evidenced based stroke care to promote functional recovery and patient satisfaction in inpatient rehabilitation | | Guideline and treatment recommendations  Functional Electrical Stimulation (FES)  Splints  Various rehabilitation equipment including steps, treadmills, heat/ice, and constraint induced therapy equipment | | 18 specific treatment recommendations (see publication for full list) | | Nurses  Therapists including Physiotherapists and Occupational Therapists | | Face to face treatment in inpatient rehabilitation setting | | Inpatient rehabilitation settings across 20 rehabilitation units in Canada | | Across duration of rehabilitation care | | Yes  Treatments could adapt following clinical assessment and adapted to needs of patient. (treatment categories in the guideline rather than fixed interventions) | | National (Canadian) stroke Best Practice Guidelines^1^ |
| References | 1. Hebert D, Lindsay MP, McIntyre A, Kirton A, Rumney PG, Bagg S, et al. Canadian stroke best practice recommendations: stroke rehabilitation practice guidelines, update 2015. Int J Stroke. 2016;11:459–84. | | | | | | | | | | | | | | | | | | |
| Savoie et al. 2019 | Pain best practice in the inpatient rehabilitation of persons with Spinal Cord Injury (SCI) | | Provide best practice interdisciplinary pain management for patients post SCI | | Assessment forms in patient’s medical chart  ISCIPBDS 2.0 assessment tool  Interdisciplinary Pain Treatment Plan (IPTP)  Pain discharge plan | | (i) Assessment of pain on admission, (ii) development of an Interdisciplinary Pain Treatment Plan (IPTP),  (iii) daily pain intensity monitoring, and  (iv) development of a pain discharge plan | | All health professionals who assess and/or treat pain  Including:  Nursing  Occupational Therapy  Physiatry  Physiotherapy  Psychology | | Face to face care delivery and clinical documentation processes | | Inpatient neurological rehabilitation unit in Canada | | Daily for pain intensity monitoring  Less frequent for other components | | Yes  Pain management care plan was designed to be personalised | | A modified Delphi process identified and recommended best pain practices for inpatient with SCI |
| References | n/a - No reference included. | | | | | | | | | | | | | | | | | | |
| Scovil et al. 2019 | Pressure injury (PI) prevention best practices in spinal cord injury (SCI) rehabilitation | | Provide evidenced based care to decrease pressure injury post SCI | | Risk assessment tools  Site specific documentation templates  Patient education materials | | (i) Comprehensive PI risk assessment and prevention plan, (ii) PI prevention education to patients | | Members of interdisciplinary team including:  Medical staff  Nurses  Physical therapists  Occupational therapists  Dietitians/nutritionists  Social workers  Psychologists | | Face to face at point of care delivery and clinical documentation processes  Patient education included:  (i) Structured group sessions,  (ii) educational materials, and (iii) unstructured, individualized education | | Six Canadian SCI rehabilitation centres | | Completion of plan - once  Patient Education – unknown frequency (could include both structured and unstructured components) | | Yes  Individualised prevention plan  Unstructured education component could be tailored to individual | | 48 best practice were identified from three clinical practice guidelines^1,2,3^  A modified Delphi consensus process was used to select best practices to include in this intervention |
| References | 1. Consortium for Spinal Cord Medicine. Pressure ulcer prevention and treatment following spinal cord injury: a clinical practice guideline for health-care professionals. J Spinal Cord Med 2001;24(Suppl 1):S40-101. 2. Houghton PE, Campbell KE; CPG Panel. Canadian best practice guidelines for the prevention and management of pressure ulcers in people with spinal cord injury. Available at: http://onf.org/system/attachments/168/original/Pressure_Ulcers_Best_Practice_Guideline_Final_web4.pdf. Acccessed January 12, 2018. 3. Regan M, Teasell RW, Keast D, Aubut JL, Foulon BL, Mehta S. Pressure ulcers following spinal cord injury. In: Eng JJ, Teasell RW, Miller WC, Wolfe DL, Townson AF, Hsieh JTC, Connolly SJ, Mehta S, Sakakibara BM, editors. Spinal Cord Injury Rehabilitation Evidence. Version 3.0. 2010. Available at: http://scireproject.com/wp-content/uploads/pressure_ulcers.pdf. Accessed December 18,2018. | | | | | | | | | | | | | | | | | | |
| Semin-Goossens et al. 2003 | Compliance with a guideline related to falls prevention in inpatient settings | | Reduce practice variation and improving effectiveness in relation to falls prevention | | Guideline (paper based) included in publication as a figure | | The guideline primarily existed of filling in risk-scales daily, noting the increased risk in the nursing file, and taking protective actions including: moving the bed to the lowest position; raising bed side rails; informing patient and relatives about the increased risk and measures taken; and asking relatives to help with structuring the environment (e.g., putting the bell within reach and making sure the patient’s shoes were nearby) | | Nurses | | Face to face care delivery and clinical documentation (including incident reporting) | | Two wards in a large, academic teaching hospital in Amsterdam, Netherlands | | Throughout an entire inpatient admission | | Yes  Flexible guideline with suggested interventions that could be tailored to meed the needs of the individual patient | | Stated that “t*he actions that were included*  *in the guideline were all extracted*  *from the literature on fall prevention*”.  However, no specific evidence source sited. |
| References | No reference provided | | | | | | | | | | | | | | | | | | |
| Stevens et al. 2014 | Evidence-based Practice for Improving Quality (EPIQ), on paediatric procedural pain practices (pain assessment and pain management practices) | | Provide evidenced based pain assessment and management in hospitalized children to support effective paediatric pain practices | | Various pain assessment tools including:  Faces, Legs, Activity, Cry, and Consolability (FLACC); Faces Pain Scale-Revised (FPS-R); Numeric Rating Scale (NRS); Behavioral Indicators of Infant Pain (BIIP); Multidimensional Assessment Pain Scale (MAPS); and Visual Analogue Scale (VAS)  Pharmacological strategies typically included local anaesthetic agents and sucrose | | Different units could focus of different aspects of pain assessment or a type of intervention to manage pain  Of the 16 EPIQ units, 11 focused on pain assessment, 5 on pharmacological interventions, and 2 on physical interventions  None of the EPIQ units focused on psychological interventions | | Multidisciplinary teams | | Face to face care delivery at point of care and/or clinical documentation | | 32 inpatient units (10 critical care, 8 surgical and 14 medical units) from 8 paediatric hospitals across Canada | | During inpatient admission | | Yes  Each unit could focus on different aspect of pain assessment or type of intervention to manage pain based on baseline data | | Evidence used incorporated evidence-based pain management strategies, validated pain assessment tools, and evidence-based pain standards and guidelines^1,2,3,4,5^ |
| References | 1. American Academy of Pediatrics Committee on Fetus and Newborn, Section on Surgery, and Section on Anesthesiology and Pain Medicine, Canadian Pediatric Society Fetus and Newborn Committee. Prevention and management of pain in the neonate: an update. Pediatrics, 2006;118:2231–41. 2. American Academy of Pediatrics Committee on Psychosocial Aspects of Child and Family Health Task Force on Pain in Infants, Children, and Adolescents. The assessment and management of acute pain in infants, children, and adolescents. Pediatrics, 2001;108:793–7. 3. Stevens BJ, Pillai Riddell RR, Oberlander TE, Gibbins S. Assessment of pain in neonates and infants. In: Anand KJS, Stevens BJ, McGrath PJ, editors. Pain in neonates and infants. 3rd ed. Philadelphia, PA, USA: Elsevier; 2007. p. 67–90. 4. Taylor EM, Boyer K, Campbell FA. Pain in hospitalized children: a prospective cross-sectional survey of pain prevalence, intensity, assessment and management in a Canadian pediatric teaching hospital. Pain Res Manag 2008;13:25–32. 5. Yamada J, Stinson J, Lamba J, Dickson A, McGrath PJ, Stevens B. A review of systematic reviews on pain interventions in hospitalized infants. Pain Res Manag 2008;13:413–20. | | | | | | | | | | | | | | | | | | |
| Sving et al. 2016 | Evidence-based pressure ulcer prevention | | Provide evidenced based care to decrease pressure injury in an inpatient setting | | Modified Norton Scale (a pressure injury risk assessment tool)  Provided list of examples of equipment for pressure relief including: sliding sheets; pressure distributing material; and material to off load heels | | Patients at risk of developing pressure ulcers were assessed using the Modified Norton Scale  Pressure ulcer prevention as follows: (i) risk and skin assessment, (ii) use of pressure redistributing material in bed and in chair, (iii) pressure relief/offload of heels, (iv)  a turning schedule available at the bedside, (v) use of sliding sheets in bed, and (vi) use of other equipment to reduce pressure on the skin | | Nurses  Nursing assistants | | Face to face care delivery at point of care or clinical documentation | | Five units (three surgical and two medical units) in a 344-bed general hospital in Sweden | | Throughout admission  Risk and skin assessment documented within 24 hours of admission to the unit  Schedule for turning patient when in bed and sitting out in chair | | Yes  Pressure prevention interventions could be tailored | | Hospital pressure ulcer prevention guideline developed using international^1^ and national (Swedish)^2^ guidelines |
| References | 1. National Pressure Ulcer Advisory Panel & European Pressure Ulcer Advisory Panel. Pressure ulcer prevention & treatment. Clinical Practice Guideline. Washington, DC: National Pressure Ulcer Advisory Panel & European Pressure Ulcer Advisory Panel, 2009. 2. Swedish Association of Local Authorities and Regions. Nationell satsning för ökad patientsäkerhet. Trycksår.Åtgärder för att förebygga. Ljungbergs Tryckeri, 2011. | | | | | | | | | | | | | | | | | | |
| Taylor et al. 2013 | Reducing the harm caused by misplaced nasogastric (NG) feeding tubes | | Evidence-based guidance to reduce the risk of feeding into misplaced nasogastric feeding tubes | | Guideline  pH testing paper  X-Ray | | Guideline recommends that the first line method for confirming tube position should be to check the pH of stomach aspirate  If the pH is >5.5, or obtaining an aspirate is not possible, it is only then appropriate to request an X-ray to check the tube position | | Multidisciplinary clinicians  Publication was not explicit about the individual professional groups responsible for intervention | | Face to face at point of care | | In-patient hospital setting across three UK hospitals | | Once at time of nasogastric line insertion | | Yes  If obtaining an aspirate is not possible then recommended first line check for confirming tube position could be missed and an X-ray ordered to check the tube position | | Clinical guideline released by the National (UK) Patient Safety Agency (NPSA)^1^ |
| References | 1. NHS - National Patient Safety Agency. Patient Safety Alert NPSA/2011/PSA002: Reducing the harm caused by misplaced nasogastric feeding tubes in adults, children and infants. March 2011. | | | | | | | | | | | | | | | | | | |
| Thomas et al. 2016 | Compliance with intervention to improve physical therapists’ adherence to key guideline recommendations for managing risk of falls on discharge from hospital | | Provide evidenced based physiotherapy interventions to older adults at risk of falls during the discharge from hospital transition period | | Modification of an existing standardized assessment proforma (including prompts and possible actions)  Development of standardized processes for transfer of information to community service providers (paper and electronic options)  Intervention processes incorporated into standardized operating procedures (including the department’s orientation manual) | | Assessment of falls risk prior to discharge from acute care  For those patients assessed at risk development and handover of a management plan to prevent falls post discharge | | Physical Therapists | | Face to face at point of care and clinical documentation processes | | Acute care setting for all admissions at risk of falls at an acute teaching hospital in South Australia, Australia. | | Once prior to discharge from acute care | | Yes  At patient level management plan designed to be individualised to needs of patient | | National (Australian) commission for quality and safety in healthcare best practice guidelines and standards^1,2^ |
| References | 1. Australian Commission on Safety and Quality in Healthcare. Preventing Falls and Harm From Falls: Best Practice Guidelines for Australian Hospitals 2009. Available at:http://www.safetyandquality.gov.au/wpcontent/uploads/2012/01/Guidelines-HOSP1.pdf. Accessed August 2015. 2. Australian Commission on Safety and Quality in Healthcare. National Safety and Quality Health Service Standards. September 2011. Available at: http://www.safetyandquality.gov.au/wp-content/uploads/2011/01/NSQHS-Standards-Sept2011.pdf. Accessed August 2015. | | | | | | | | | | | | | | | | | | |
| Tian et al. 2017 | Model of Cancer Related Fatigue management in hospitalized adult patients | | Provide evidenced based screening, assessment and interventions in relation to cancer related fatigue | | Cancer related fatigue screening and assessment tools  Cancer related fatigue intervention procedure and associated material required for individual interventions (details not provided in publication)  Nursing record chart  Cancer related fatigue health education consumer booklet | | Screening and assessment for  cancer related fatigue  Implementing cancer related fatigue interventions e.g. health education, exercise, and music therapy | | Nurses | | Face to face at point of care in inpatient setting | | One Oncology and one radiotherapy ward in an adult university-affiliated hospital in Suzhou, China | | Multiple times however unable to quantify as this level of detail not provided in publication | | Yes  Interventions could be personalised to needs of individual patient | | Evidence derived from an international clinical practice guideline^1^ and systematic reviews^2,3,4,5^ |
| References | 1. Berger AM, Abernethy AP, Atkinson A, Barsevick AM, Breitbart WS, Cella D, et al. Cancer-related fatigue. J Nalt Compr Canc Netw. 2010; 8: 904-931. https://doi.org/10.6004/jnccn.2010.00674. 2. Seyidova-Khoshknabi D, Davis MP, Walsh D. Review article: A systematic review of cancer-related fatigue measurement questionnaires. Am J Hosp Palliat Care. 2011; 28: 119-129. https://doi.org/10.1177/1049909110381590 PMID: 21051784 3. de Nijs EJ, Ros W, Grijpdonck MH. Nursing intervention for fatigue during the treatment for cancer. Cancer Nurs. 2008; 31: 191-206. https://doi.org/10.1097/01.NCC.0000305721.98518.7c PMID: 18453875 4. Larkin D, Lopez V, Aromataris E. Managing cancer-related fatigue in men with prostate cancer: A systematic review of non-pharmacological interventions. Int J Nurs Practice. 2014; 20: 549-560. https://doi.org/10.1111/ijn.12211 PMID: 24237792 5. Su CX, Wang LQ, Grant SJ, Liu JP. Chinese herbal medicine for cancer-related fatigue: A systematic review of randomized clinical trials. Complement Ther Med. 2014; 22: 567-579. https://doi.org/10.1016/j.ctim.2014.04.007 PMID: 24906595 | | | | | | | | | | | | | | | | | | |
| White et al. 2019 | World Health Organisation (WHO) Surgical Safety Checklist | | Provide evidence based perioperative quality and safety interventions for surgical patients | | Surgical checklist  Pulse oximeters | | Complete a surgical safety checklist prior to every surgical procedure | | All staff working in a surgical environment | | Checklist used in surgical context face to face at point of care | | 36 hospitals that represented the majority of government surgical hospitals in Benin | | Once for each patient undergoing surgery | | Yes  Surgical Checklist was adapted to the local context at each hospital | | World Health Organisation guidelines for safe surgery^1,2^, systematic review^3^, and stepped wedge cluster randomised controlled trial^4^ measuring the effect of the WHO surgical safety checklist |
| References | 1. WHO. WHO Guidelines for Safe Surgery.WHO Press: Geneva, 2009. 2. Haynes AB,Weiser TG, Berry WR, Lipsitz SR, Breizat AS, Dellinger P et al. A surgical safety checklist to reduce morbidity and mortality in a global population. N Engl J Med 2009; 360: 491–499. 3. Bergs J, Hellings J, Cleemput I, Zurel Ö, De Troyer V, Van Hiel M et al. Systematic review and meta-analysis of the effect of the World Health Organization surgical safety checklist on postoperative complications. Br J Surg 2014; 101: 150–158. 4. Haugen AS, Søfteland E, Almeland SK, Sevdalis N, Vonen B, Eide GE et al. Effect of the World Health Organization checklist on patient outcomes: a stepped wedge cluster randomized controlled trial. Ann Surg 2015; 261: 821–828. | | | | | | | | | | | | | | | | | | |
| White et al. 2020 | World Health Organisation (WHO) Surgical Safety Checklist | | Provide evidence based perioperative quality and safety interventions | | Surgical checklist and counting sheet  Pulse oximeters | | Complete a surgical safety checklist prior to every surgical procedure | | Surgeons  Anaesthesia providers  Nurses  Other surgical staff including: biomedical technicians; sterile processing technicians; medical or nursing students | | Checklist used in surgical context face to face at point of care | | 25 surgical hospitals across Cameroon | | Once for each patient undergoing surgery | | Yes  Surgical checklist and counting sheet adapted to the local context at each hospital | | Systematic reviews^1,2^ |
| References | 1. Bergs J, Hellings J, Cleemput I, et al. Systematic review and meta-analysis of the effect of the World Health Organization Surgical Safety Checklist on postoperative complications. Br J Surg. 2014;101:150–158. 2. Borchard A, Schwappach DL, Barbir A, Bezzola P. A systematic review of the effectiveness, compliance, and critical factors for implementation of safety checklists in surgery. Ann Surg. 2012;256:925–933. | | | | | | | | | | | | | | | | | | |

***Supplemental Table 8 – Risk of Bias across included studies***

| **Study / Year** | **Inclusion/ exclusion criteria clearly stated** | **Recruitment/**  **allocation**  **consistency** | **Variations**  **in protocol** | **Outcome**  **assessor**  **blinding** | **Valid and**  **reliable**  **measures** | **Length of**  **follow up**  **across**  **groups** | **Incomplete outcome data** | **Selective**  **reporting** | **Confounding**  **variables** | **Other**  **sources of**  **bias** |
| --- | --- | --- | --- | --- | --- | --- | --- | --- | --- | --- |
| ***Process Models*** | | | | | | | | | | |
| Azar et al. 2019 | No | High | High | High | High | High | High | High | High | High |
| Cody et al. 2021 | No | High | High | High | Low | Low | Low | Low | High | Low |
| Gerrish et al. 2016 | No | High | High | High | Low | Low | Low | High | High | Low |
| Peterson et al. 2017 | Yes | Low | High | High | Low | High | Low | Low | High | Low |
| Rattray et al. 2021 | Yes | Low | High | High | Low | High | Low | Low | High | Low |
| Reynolds et al. 2018 | Yes | Low | High | High | Low | Low | Low | Low | High | Low |
| Salbach et al. 2017 | Yes | Low | High | High | Low | Low | Low | High | Low* | Low |
| Semin-Goossens et al. 2003 | Yes | Low | High | High | High | High | Low | Low | High | High |
| White et al. 2020 | Yes | Low | High | High | Low | High | High | Low | High | Low |
| ***Determinant Frameworks*** | | | | | | | | | | |
| Byrnes et al. 2018 | No | High | High | High | High | Low | Low | Low | High | Low |
| Garrido et al. 2021 | Yes | High | High | High | High | Low | Low | High | High | Low |
| Gu et al. 2020 | Yes | Low | High | High | Low | Low | Low | Low | Low | Low |
| Peel et al. 2021 | Yes | Low | High | High | Low | High | Low | Low | High | Low |
| Savoie et al. 2019 | Yes | Low | High | High | High | High | Low | Low | High | Low |
| Scovil et al. 2019 | Yes | Low | High | High | High | High | Low | Low | High | Low |
| Stevens et al. 2014 | Yes | Low | High | High | Low | High | Low | Low | Low | Low |
| Sving et al. 2016 | Yes | Low | High | High | Low | Low | Low | Low | Low | Low |
| Taylor et al. 2013 | Yes | Low | High | High | Low | High | Low | High | High | Low |
| Thomas et al. 2016 | Yes | High | High | High | Low | High | Low | High | High | Low |
| Tian et al. 2017 | Yes | High | High | High | Low | High | Low | High | High | High |
| White et al. 2019 | Yes | Low | High | High | Low | High | High | Low | High | Low |
| ***> 1 Frameworks*** | | | | | | | | | | |
| Bosch et al. 2019 | Yes | low | low | low | low | low | low | low | low | low |
| Roberts et al. 2019 | Yes | Low | Low | High | Low | Low | High | Low | High | Low |
| Robertson, 2018 | Yes | Low | High | High | High | Low | Low | High | High | Low |
| Romney et al. 2019 | Yes | Low | High | High | High | Low | Low | High | High | High |

*Note: *Salbach 2017 Cluster randomised trial with random allocation.*Bosch 2019 - Cluster randomised trial with random and concealed allocation. Random allocation n/a for all other studies.*

***Supplemental Table 9: Adapted Wider(1) checklist with studies organised by framework category***

| Study / Year | Framework | Framework Components | Fidelity to implementation strategy |
| --- | --- | --- | --- |
| **Process Models** | | | |
| Azar 2019 | Agile Implementation Model | All | ? |
| Cody 2021 | JBI Implementation Framework | All | √  training only |
| Gerrish 2016 | KTA | 5/8 of action cycle. Merged 3 stages | ? |
| Peterson 2017 | KTA | ? | ? |
| Rattray 2021 | KTA | 6/8 stages of action cycle. Missing: identify, review and select knowledge; and sustain knowledge use | √  Process evaluation paper |
| Reynolds 2019 | Grol and Wensing | ? Model provided overall guidance for this study | √  training only |
| Salbach 2017 | KTA | ? | ? |
| Semin-Goossens 2003 | Grol’s 5-step implementation model | All | ? |
| White 2020 | KTA | All | ? |
| **Determinant Frameworks** | | | |
| Byrnes 2018 | i-PARiHS  No | All | ? |
| Garrido 2021 | CFIR  No | All | √  training only |
| Gu 2020 | iPARiHS | Innovation, recipient and context. Limited discussion regarding facilitation. | ? |
| Peel 2021 | iPARiHS | All | ? |
| Savoie 2019 | Active implementation frameworks | All | ? |
| Scovil 2019 | Active implementation frameworks | All | ? |
| Stevens 2014 | PARiHS | All. However, study focused on evidence and facilitation | ? |
| Sving 2016 | PARiHS | All | √  training |
| Taylor 2013 | TDF | ? Informed by the TDF and also drew upon principles derived from number of implementation theories | ? |
| Thomas 2016 | TDF  (embedded within 4 stage implementation planning process) | 12/14 TDF Domains | ? |
| Tian 2017 | PARiHS | All | ? |
| White 2019 | CFIR | All | ? |
| **>1 Framework across framework categories** | | | |
| Bosch 2019 | TDF | All | √  Supplementary files detail deviations from published protocol |
|  | Model of Diffusion of Innovations in Service Organisations | All |  |
| Roberts 2019 | TDF embedded within a systematic 4-step approach | Four phases consistent with French’s four-step approach.(29)  All components of the TDF | √  Process evaluation paper |
|  | KTA | ? |  |
| Robertson 2018 | KTA | Missing sustaining use over time | √  Education |
|  | TDF | All to guiding identification of barriers |  |
| Romney 2020 | TDF | All in interviews & 8 domains guided questionnaire | ? |
|  | KTA | All |  |

√: reported, X: Not reported, ?: Unable to determine; i-PARiHS: Integrated promoting action on research implementation in health services, PARiHS: promoting action on research implementation in health services, CFIR: Consolidated framework for implementation research, KTA: Knowledge to action.

***Supplemental Table 10: Implementation strategies used within individual studies mapped to EPOC(2) taxonomy and scored based on elements from the Wider(1) checklist***

|  |  | Audit and feedback | Clinical incident reporting | Monitoring the performance of the delivery of healthcare | Communities of practice | Continuous quality | Educational games | Educational materials | Educational meetings | Educational outreach visits, or academic detailing. | Clinical Practice Guidelines | Inter-professional education | Local consensus processes | Local opinion leaders | Managerial supervision | Patient-mediated interventions | Public release of performance data | Reminders | Routine patient-reported outcome measures | Tailored interventions |
| --- | --- | --- | --- | --- | --- | --- | --- | --- | --- | --- | --- | --- | --- | --- | --- | --- | --- | --- | --- | --- |
| **Process Models** | | | | | | | | | | | | | | | | | | | | |
| Azar 2019 | 1. Characteristics those delivering implementation strategy | - | - | - |  | + |  |  |  |  | + | - | + | + |  |  |  |  |  |  |
|  | 2. Characteristics of the recipients | + | + | + |  | - |  |  |  |  | - | + | - | - |  |  |  |  |  |  |
|  | 3. The mode of delivery | - | - | - |  | + |  |  |  |  | - | - | + | - |  |  |  |  |  |  |
|  | 4. The intensity | + | + | - |  | - |  |  |  |  | - | - | - | - |  |  |  |  |  |  |
|  | | | | | | | | | | | | | | | | | | | | |
| Cody 2021 | 1. Characteristics those delivering implementation strategy | + |  | - |  |  |  | + |  |  | - | + | + | + |  | + |  | + |  | + |
|  | 2. Characteristics of the recipients | + |  | - |  |  |  | - |  |  | + | + | + | + |  | + |  | + |  | + |
|  | 3. The mode of delivery | + |  | - |  |  |  | + |  |  | - | + | + | - |  | + |  | + |  | + |
|  | 4. The intensity | + |  | + |  |  |  | - |  |  | - | + | - | - |  | - |  | + |  | - |
|  | | | | | | | | | | | | | | | | | | | | |
| Gerrish 2016 | 1. Characteristics those delivering implementation strategy | + |  |  |  |  |  | + |  |  |  |  |  | + | - |  |  |  |  |  |
|  | 2. Characteristics of the recipients | + |  |  |  |  |  | + |  |  |  |  |  | + | + |  |  |  |  |  |
|  | 3. The mode of delivery | - |  |  |  |  |  | - |  |  |  |  |  | - | - |  |  |  |  |  |
|  | 4. The intensity | - |  |  |  |  |  | - |  |  |  |  |  | - | - |  |  |  |  |  |
|  | | | | | | | | | | | | | | | | | | | | |

| Peterson 2017 | 1. Characteristics those delivering implementation strategy | + |  |  |  |  |  | + | + |  |  |  |  | + |  |  |  | - |  | + |
| --- | --- | --- | --- | --- | --- | --- | --- | --- | --- | --- | --- | --- | --- | --- | --- | --- | --- | --- | --- | --- |
|  | 2. Characteristics of the recipients | + |  |  |  |  |  | + | + |  |  |  |  | + |  |  |  | + |  | + |
|  | 3. The mode of delivery | + |  |  |  |  |  | + | + |  |  |  |  | + |  |  |  | + |  | - |
|  | 4. The intensity | - |  |  |  |  |  | - | + |  |  |  |  | - |  |  |  | - |  | - |
|  | | | | | | | | | | | | | | | | | | | | |
| Rattray 2021 | 1. Characteristics those delivering implementation strategy |  |  |  |  |  |  |  |  |  |  | + |  |  |  | + |  |  |  |  |
|  | 2. Characteristics of the recipients |  |  |  |  |  |  |  |  |  |  | + |  |  |  | + |  |  |  |  |
|  | 3. The mode of delivery |  |  |  |  |  |  |  |  |  |  | + |  |  |  | + |  |  |  |  |
|  | 4. The intensity |  |  |  |  |  |  |  |  |  |  | + |  |  |  | - |  |  |  |  |
|  | | | | | | | | | | | | | | | | | | | | |
| Reynolds 2019 | 1. Characteristics those delivering implementation strategy | + |  |  |  |  |  | + | + |  |  |  |  | + |  |  |  |  |  |  |
|  | 2. Characteristics of the recipients | + |  |  |  |  |  | + | + |  |  |  |  | + |  |  |  |  |  |  |
|  | 3. The mode of delivery | + |  |  |  |  |  | + | + |  |  |  |  | + |  |  |  |  |  |  |
|  | 4. The intensity | + |  |  |  |  |  | + | + |  |  |  |  | - |  |  |  |  |  |  |
|  | | | | | | | | | | | | | | | | | | | | |
| Salbach 2017 | 1. Characteristics those delivering implementation strategy |  |  |  | + |  |  | - | + |  | + |  |  | + |  |  |  | + |  | + |
|  | 2. Characteristics of the recipients |  |  |  | + |  |  | + | + |  | + |  |  | + |  |  |  | + |  | + |
|  | 3. The mode of delivery |  |  |  | + |  |  | + | + |  | + |  |  | + |  |  |  | + |  | + |
|  | 4. The intensity |  |  |  | - |  |  | - | - |  | - |  |  | - |  |  |  | - |  | - |
|  | | | | | | | | | | | | | | | | | | | | |
| Semin-Goossens 2003 | 1. Characteristics those delivering implementation strategy | - |  |  |  | - |  |  |  |  | + |  |  | + |  |  |  | - |  | + |
|  | 2. Characteristics of the recipients | + |  |  |  | + |  |  |  |  | + |  |  | + |  |  |  | + |  | + |
|  | 3. The mode of delivery | - |  |  |  | + |  |  |  |  | + |  |  | + |  |  |  | + |  | + |
|  | 4. The intensity | + |  |  |  | - |  |  |  |  | - |  |  | + |  |  |  | + |  | - |
|  | | | | | | | | | | | | | | | | | | | | |
| White 2020 | 1. Characteristics those delivering implementation strategy |  |  |  | + |  |  |  |  | + |  | + |  | + |  |  |  | + |  | + |
|  | 2. Characteristics of the recipients |  |  |  | + |  |  |  |  | + |  | + |  | + |  |  |  | + |  | + |
|  | 3. The mode of delivery |  |  |  | + |  |  |  |  | + |  | + |  | + |  |  |  | + |  | + |
|  | 4. The intensity |  |  |  | + |  |  |  |  | + |  | + |  | + |  |  |  | - |  | + |
| Proportion of studies that used a Process model to use each EPOC strategy | | 67% | 11% | 22% | 22% | 22% | 0% | 56% | 33% | 11% | 44% | 44% | 22% | 89% | 11% | 22% | 0% | 56% | 0% | 56% |

| **Determinant Frameworks** | | | | | | | | | | | | | | | | | | | | |
| --- | --- | --- | --- | --- | --- | --- | --- | --- | --- | --- | --- | --- | --- | --- | --- | --- | --- | --- | --- | --- |
| Byrnes 2018 | 1. Characteristics those delivering implementation strategy | + |  |  |  |  |  | + |  |  | + | - |  | + |  |  |  | - |  | - |
|  | 2. Characteristics of the recipients | + |  |  |  |  |  | + |  |  | + | + |  | + |  |  |  | + |  | + |
|  | 3. The mode of delivery | + |  |  |  |  |  | + |  |  | + | + |  | - |  |  |  | + |  | + |
|  | 4. The intensity | - |  |  |  |  |  | - |  |  | - | - |  | - |  |  |  | - |  | - |
|  | | | | | | | | | | | | | | | | | | | | |
| Garrido 2021 | 1. Characteristics those delivering implementation strategy |  |  |  |  |  |  | - | - |  | - | - |  | - |  |  |  |  |  |  |
|  | 2. Characteristics of the recipients |  |  |  |  |  |  | + | + |  | + | + |  | + |  |  |  |  |  |  |
|  | 3. The mode of delivery |  |  |  |  |  |  | - | + |  | - | - |  | + |  |  |  |  |  |  |
|  | 4. The intensity |  |  |  |  |  |  | - | - |  | - | - |  | - |  |  |  |  |  |  |
|  | | | | | | | | | | | | | | | | | | | | |
| Gu 2020 | 1. Characteristics those delivering implementation strategy |  |  |  |  |  |  | - | - |  | - |  |  |  |  |  |  | + |  |  |
|  | 2. Characteristics of the recipients |  |  |  |  |  |  | + | + |  | + |  |  |  |  |  |  | + |  |  |
|  | 3. The mode of delivery |  |  |  |  |  |  | - | + |  | - |  |  |  |  |  |  | - |  |  |
|  | 4. The intensity |  |  |  |  |  |  | - | + |  | - |  |  |  |  |  |  | - |  |  |
|  | | | | | | | | | | | | | | | | | | | | |
| Peel 2021 | 1. Characteristics those delivering implementation strategy |  |  |  |  |  |  | - | + |  |  |  |  | + |  |  |  | + |  |  |
|  | 2. Characteristics of the recipients |  |  |  |  |  |  | + | + |  |  |  |  | + |  |  |  | - |  |  |
|  | 3. The mode of delivery |  |  |  |  |  |  | + | + |  |  |  |  | - |  |  |  | - |  |  |
|  | 4. The intensity |  |  |  |  |  |  | - | - |  |  |  |  | - |  |  |  | - |  |  |
|  | | | | | | | | | | | | | | | | | | | | |
| Savoie 2019 | 1. Characteristics those delivering implementation strategy | + |  |  | + |  |  |  | + |  |  |  | + | + |  |  |  | + |  | + |
|  | 2. Characteristics of the recipients | + |  |  | + |  |  |  | + |  |  |  | + | + |  |  |  | + |  | + |
|  | 3. The mode of delivery | - |  |  | - |  |  |  | + |  |  |  | + | + |  |  |  | + |  | + |
|  | 4. The intensity | + |  |  | - |  |  |  | - |  |  |  | + | + |  |  |  | + |  | + |
|  | | | | | | | | | | | | | | | | | | | | |
| Scovil 2019 | 1. Characteristics those delivering implementation strategy | - |  |  | + | - |  |  | + | + |  |  | - | + |  | - |  |  |  | + |
|  | 2. Characteristics of the recipients | + |  |  | + | + |  |  | + | + |  |  | - | + |  | + |  |  |  | + |
|  | 3. The mode of delivery | - |  |  | - | + |  |  | - | - |  |  | + | - |  | + |  |  |  | + |
|  | 4. The intensity | - |  |  | - | - |  |  | - | - |  |  | - | - |  | + |  |  |  | + |
|  | | | | | | | | | | | | | | | | | | | | |
| Stevens 2014 | 1. Characteristics those delivering implementation strategy | + |  |  |  | + |  | - |  | + |  |  |  | + |  |  |  | - |  | + |
|  | 2. Characteristics of the recipients | + |  |  |  | + |  | - |  | - |  |  |  | + |  |  |  | - |  | + |
|  | 3. The mode of delivery | + |  |  |  | + |  | + |  | + |  |  |  | - |  |  |  | + |  | - |
|  | 4. The intensity | + |  |  |  | + |  | - |  | - |  |  |  | - |  |  |  | - |  | + |
|  | | | | | | | | | | | | | | | | | | | | |
| Sving 2016 | 1. Characteristics those delivering implementation strategy |  |  |  |  | + |  |  | + | + |  |  |  | + |  |  |  |  |  | + |
|  | 2. Characteristics of the recipients |  |  |  |  | + |  |  | + | + |  |  |  | + |  |  |  |  |  | + |
|  | 3. The mode of delivery |  |  |  |  | + |  |  | - | - |  |  |  | + |  |  |  |  |  | + |
|  | 4. The intensity |  |  |  |  | + |  |  | + | + |  |  |  | + |  |  |  |  |  | + |
|  | | | | | | | | | | | | | | | | | | | | |
| Taylor 2013 | 1. Characteristics those delivering implementation strategy | + |  |  |  |  |  | + | + |  | + |  | + | + |  |  |  | + |  | + |
|  | 2. Characteristics of the recipients | - |  |  |  |  |  | + | + |  | - |  | + | + |  |  |  | + |  | + |
|  | 3. The mode of delivery | + |  |  |  |  |  | + | + |  | - |  | + | - |  |  |  | + |  | + |
|  | 4. The intensity | + |  |  |  |  |  | + | - |  | - |  | - | - |  |  |  | - |  | + |
|  | | | | | | | | | | | | | | | | | | | | |

| Thomas 2014 | 1. Characteristics those delivering implementation strategy | + |  |  |  |  |  | + | - |  | - |  |  | + |  | + |  | - |  | + |
| --- | --- | --- | --- | --- | --- | --- | --- | --- | --- | --- | --- | --- | --- | --- | --- | --- | --- | --- | --- | --- |
|  | 2. Characteristics of the recipients | + |  |  |  |  |  | + | + |  | + |  |  | + |  | + |  | + |  | + |
|  | 3. The mode of delivery | + |  |  |  |  |  | + | + |  | - |  |  | + |  | + |  | + |  | + |
|  | 4. The intensity | + |  |  |  |  |  | + | + |  | + |  |  | + |  | + |  | + |  | + |
|  | | | | | | | | | | | | | | | | | | | | |
| Tian 2017 | 1. Characteristics those delivering implementation strategy |  |  |  |  |  |  | - | + | + | + |  |  | + |  | + |  |  |  | + |
|  | 2. Characteristics of the recipients |  |  |  |  |  |  | + | + | + | + |  |  | + |  | + |  |  |  | + |
|  | 3. The mode of delivery |  |  |  |  |  |  | + | + | + | - |  |  | + |  | + |  |  |  | + |
|  | 4. The intensity |  |  |  |  |  |  | + | + | - | + |  |  | - |  | + |  |  |  | + |
|  | | | | | | | | | | | | | | | | | | | | |
| White 2019 | 1. Characteristics those delivering implementation strategy |  |  |  | + |  |  | + |  | + |  | + |  | + |  |  |  | + |  |  |
|  | 2. Characteristics of the recipients |  |  |  | + |  |  | + |  | + |  | + |  | + |  |  |  | + |  |  |
|  | 3. The mode of delivery |  |  |  | + |  |  | + |  | + |  | + |  | - |  |  |  | + |  |  |
|  | 4. The intensity |  |  |  | + |  |  | - |  | - |  | + |  | + |  |  |  | - |  |  |
| Proportion of studies that used a Determinant framework to use each EPOC strategy | | 50% | 0% | 0% | 25% | 25% | 0% | 75% | 75% | 42% | 50% | 25% | 25% | 92% | 0% | 25% | 0% | 67% | 0% | 67% |
| **>1 Framework across framework categories** | | | | | | | | | | | | | | | | | | | | |
| Bosch 2019 | 1. Characteristics those delivering implementation strategy |  |  |  |  |  |  | + | + | + | + |  |  | + |  |  |  | + |  | + |
|  | 2. Characteristics of the recipients |  |  |  |  |  |  | + | + | + | + |  |  | + |  |  |  | + |  | + |
|  | 3. The mode of delivery |  |  |  |  |  |  | + | + | + | + |  |  | + |  |  |  | + |  | + |
|  | 4. The intensity |  |  |  |  |  |  | + | + | + | + |  |  | + |  |  |  | + |  | + |
|  | | | | | | | | | | | | | | | | | | | | |
| Roberts 2019 | 1. Characteristics those delivering implementation strategy |  |  |  |  |  |  |  |  |  |  | + |  |  |  |  |  | + |  | + |
|  | 2. Characteristics of the recipients |  |  |  |  |  |  |  |  |  |  | + |  |  |  |  |  | + |  | + |
|  | 3. The mode of delivery |  |  |  |  |  |  |  |  |  |  | + |  |  |  |  |  | + |  | + |
|  | 4. The intensity |  |  |  |  |  |  |  |  |  |  | + |  |  |  |  |  | - |  | - |
|  | | | | | | | | | | | | | | | | | | | | |
| Robertson 2018 | 1. Characteristics those delivering implementation strategy | - |  |  |  |  |  |  |  |  |  | + |  |  |  |  |  |  |  | + |
|  | 2. Characteristics of the recipients | + |  |  |  |  |  |  |  |  |  | + |  |  |  |  |  |  |  | + |
|  | 3. The mode of delivery | + |  |  |  |  |  |  |  |  |  | - |  |  |  |  |  |  |  | + |
|  | 4. The intensity | + |  |  |  |  |  |  |  |  |  | + |  |  |  |  |  |  |  | - |
|  | | | | | | | | | | | | | | | | | | | | |
| Romney 2019 | 1. Characteristics those delivering implementation strategy | + |  | + |  |  |  | + | + |  |  |  | + | + |  |  |  | + |  | + |
|  | 2. Characteristics of the recipients | + |  | + |  |  |  | + | + |  |  |  | + | + |  |  |  | + |  | + |
|  | 3. The mode of delivery | + |  | + |  |  |  | + | + |  |  |  | + | + |  |  |  | + |  | + |
|  | 4. The intensity | + |  | + |  |  |  | - | + |  |  |  | + | + |  |  |  | + |  | + |
| Proportion of studies that used >1 framework to use each EPOC strategy | | 50% | 0% | 25% | 0% | 0% | 0% | 50% | 50% | 25% | 25% | 50% | 25% | 50% | 0% | 0% | 0% | 75% | 0% | 100% |

+Details regarding implementation strategy provided, - Details regarding implementation strategy not provided

***Supplemental Table 11: All process of care outcomes***

| **Study / Year** | **Patients**  **n** | **Theoretical Implementation Framework** | **Target process of care outcome** | **How process of care was measured** | **Pre-impl. compliance**  **n (%)** | **Post-impl.**  **compliance**  **n (%)** | **Comparison unit compliance**  **n (%)** | **Results:** |
| --- | --- | --- | --- | --- | --- | --- | --- | --- |
| **Process Models** | | | | | | | | |
| Azar  et al. 2019 | N/R | Agile Implementation Model | Central line bundle compliance (Daily chlorhexidine gluconate bathing, appropriate dressing, tubing changes, skin care, and hub disinfection) ** | Audit nursing maintenance bundle checklist | N/R  (67%) | N/R  (84%) | N/A | **Pre vs. post**  *p<0.001 ^α^* |
| Cody et al. 2021 | 294 | JBI evidence implementation framework | Compliance with screening for delirium using a validated tool * | Retrospective Chart Audit | 30/143  21% | 104/151  69% | N/A | **Pre vs. post**  OR 8.33 (4.91, 14.16) |
|  |  |  | Patient/carer asked about any recent changes in behaviour or thinking |  | 49/137  36% | 100/149  67% | N/A | **Pre vs. post**  OR 3.67 (2.25, 5.98) |
|  |  |  | Compliance with assessment using a validated tool |  | 10/114  9% | 90/128  70% | N/A | **Pre vs. post**  OR 24.63 (11.62, 52.23) |
|  |  |  | HP discuss delirium risk with patients/ carers |  | 21/126  17% | 54/139  39% | N/A | **Pre vs. post**  OR 3.18 (1.78, 5.67) |
|  |  |  | Carers provided with information about delirium |  | 7/124  6% | 11/127  9% | N/A | **Pre vs. post**  OR 1.58 (0.59, 4.23) |
|  |  |  | At risk patients offered interventions ** |  | 65/120  54% | 84/130  65% | N/A | **Pre vs. post**  OR 1.55 (0.93, 2.57) |
|  |  |  | At risk patients are monitored regularly |  | 102/122  84% | 125/132  95% | N/A | **Pre vs. post**  OR 3.50 (1.42, 8.61) |
|  |  |  | Patient’s with delirium undergo a comprehensive assessment |  | 41/66  62% | 34/36  94% | N/A | **Pre vs. post**  OR 10.37 (2.29, 46.94) |
|  |  |  | Patient’s with delirium receive a set of interventions |  | 41/66  62% | 34/37  92% | N/A | **Pre vs. post**  OR 6.91 (1.92, 24.88) |
|  |  |  | Patients with delirium are screened for falls |  | 54/66  82% | 56/56  100% | N/A | **Pre vs. post**  OR 25.92 (1.50, 448.54) |
|  |  |  | Patients with delirium are screened for pressure injuries |  | 54/66  82% | 56/57  98% | N/A | **Pre vs. post**  OR 12.44 (1.56, 99.01) |
|  |  |  | Patients with delirium receive non-pharmacological management as first line therapy |  | 36/53  68% | 32/34  94% | N/A | **Pre vs. post**  OR 7.56 (1.62, 35.26) |
|  |  |  | Patients with current or resolving delirium receive an individual care plan |  | 7/73  (10%) | 6/39  (15%) | N/A | **Pre vs. post**  OR 1.71 (0.53, 5.51) |
| Gerrish et al. 2016 | 84 | KTA | Assessment of patients at risk of malnutrition within 24 hours of admission * | Documentation audit | 20/36  (56%) | 39/48  (81%) | N/A | **Pre vs. post**  OR 3.47 (1.30, 9.22) |
| Peterson et al. 2017 | 2002 | KTA | Pain assessments using a numerical rating scale * | Documentation audit | 48/687  7% | 213/644  33% | N/A | **Pre vs. post (6 months)**  OR 6.58 (4.70, 9.21) |
|  |  |  |  |  | 48/687  7% | 242/671  36% | N/A | **Pre vs. post (12 months)**  OR 7.51 (5.38, 10.48) |
|  |  |  | Pain assessments using a numerical rating scale after receiving rescue medication |  | 27/687  4% | 97/644  15% | N/A | **Pre vs. post (6 months)**  OR 4.33 (2.79, 6.74) |
|  |  |  |  |  | 27/687  4% | 114/671  17% | N/A | **Pre vs. post (12 months)**  OR 5.00 (3.24, 7.72) |
|  |  |  | Pain assessment with any pain assessment scale / instrument (inc numeric rating scale) |  | 76/687  11% | 110/644  17% | N/A | **Pre vs. post (6 months)**  OR 1.66 (1.21, 2.27) |
|  |  |  |  |  | 76/687  11% | 141/671  21% | N/A | **Pre vs. post (12 months)**  OR 2.14 (1.58, 2.98) |
|  |  |  | Pain assessment using numeric pain scale | Patient questionnaire | 168/508  33% | 224/467  48% | N/A | **Pre vs. post (6 months)**  OR 1.87 [1.44, 2.42] |
|  |  |  |  |  | 168/508  33% | 229/457  50% | N/A | **Pre vs. post (12 months)**  OR 2.03 (1.57, 2.64) |
|  |  |  | Patients asked about current pain but not asked to use a pain assessment scale |  | 198/508  39% | 117/467  25% | N/A | **Pre vs. post (6 months)**  OR 0.52 (0.40, 0.69) |
|  |  |  |  |  | 198/508  39% | 133/457  29% | N/A | **Pre vs. post (12 months)**  OR 0.64 (0.49, 0.84) |
|  |  |  | Patients asked about pain ≥3 times during last 24 hours |  | 234/508  46% | 206/467  44% | N/A | **Pre vs. post (6 months)**  OR 0.92 (0.72, 1.19) |
|  |  |  |  |  | 234/508  46% | 192/457  42% | N/A | **Pre vs. post (12 months)**  OR 0.85 (0.66, 1.09) |
|  |  |  | Patients with pain in last 24 hours who received rescue medication and who were asked if the medication alleviated the pain appropriately ** |  | 371/508  73% | 369/467  79% | N/A | **Pre vs. post (6 months)**  OR 1.39 (1.03, 1.87) |
|  |  |  |  |  | 371/508  73% | 393/457  86% | N/A | **Pre vs. post (12 months)**  OR 2.27 (1.63, 3.15) |
|  |  |  | Patients with pain in last 24 hours who received rescue medication and who had been asked to inform the nurse if the medication did not alleviate the pain in an appropriate way |  | 401/508  79% | 383/467  82% | N/A | **Pre vs. post (6 months)**  OR 1.22 (0.89, 1.67) |
|  |  |  |  |  | 401/508  79% | 384/457  84% | N/A | **Pre vs. post (12 months)**  OR 1.40 (1.01, 1.95) |
| Rattray et al. 2021 | 64 | KTA | Time (in hours) to first prescribed diet *** | Medical record audit  Median / IQR | 6 (3-19.6)  (n=30)  Estimated Mean (SD)(40)  9.5 (12.9) | 3.1 (2.1-3.9)  (n=34)  Estimated mean / SD(40)  3.0 (1.4) | N/A | **Pre vs. post**  SMD 0.72 (0.22, 1.23) |
|  |  |  | Time (in hours) to first diet delivery ** | Direct observation supplemented where necessary by verbal clarification with nurses / patients  Median / IQR | 13.8 (6.4-21.5)  Estimated mean / SD(40)  13.9 (11.8) | 4.7 (3.3-12.8)  Estimated mean / SD(40)  6.9 (7.4) | N/A | **Pre vs. post**  SMD 0.71 (0.20, 1.22) |
|  |  |  | Time (in hours) to first prescribed solid diet | Medical record audit  Median / IQR | 86.8 (62.5-112.3)  Estimated mean / SD(40)  87.2 (38.7) | 67.1 (42.2-109.5)  Estimated mean / SD(40)  72.9 (52.1) | N/A | **Pre vs. post**  SMD 0.30 (-0.19, 0.80) |
|  |  |  | Time (in hours) to first solid diet delivery | Direct observation supplemented where necessary by verbal clarification with nurses / patients  Median / IQR | 86.1 (58.6-105.3)  Estimated mean / SD(40)  83.3 (36.4) | 69.1 (47.2-115.5)  Estimated mean / SD(40)  77.3 (52.9) | N/A | **Pre vs. post**  SMD 0.13 (-0.36, 0.62) |
|  |  |  | Free fluids prescribed post-operative day 0 | Medical record audit. | 4/30  13% | 27/34  79% | N/A | **Pre vs. post**  OR 25.07 (6.56, 95.88) |
| Reynolds et al. 2018 | N/R | Grol and Wensing | Compliance with daily bathing of central line catheter with chlorhexidine gluconate ** | Medical record audit | N/R  (57%) | N/R  (80%) | N/A | **Pre vs. post**  *p=0.013 ^α^* |
| Salbach et al. 2017 | 312 | KTA | Adherence with guidelines regarding treating sit to stand ** | Health professional self-report checklist | 132/647  (20.4%) | 108/276  (39.1%) | 89/265  (33.6%) | **Pre vs. post**  OR 2.51 (1.84, 3.41)  **Post vs. comparison**  OR 1.27 (0.89, 1.81) |
|  |  |  | Adherence with guidelines regarding treating lower extremity range of motion |  | 24/151  (15.9%) | 15/143  (10.5%) | 24/135  (17.8%) | **Pre vs. post**  OR 0.62 (0.31, 1.24)  **Post vs. comparison**  OR 0.54 (0.27, 1.08) |
|  |  |  | Adherence with guidelines regarding use of lower extremity support |  | 47/647  (7.3%) | 24/276  (8.7%) | 46/265  (17.4%) | **Pre vs. post**  OR 1.22 (0.73, 2.03)  **4Post vs. comparison**  OR 0.45 (0.27, 0.77) |
|  |  |  | Adherence with guidelines regarding task specific training i.e. stairs |  | 48/151  (31.8%) | 55/143  (38.5%) | 51/135  (37.8%) | **Pre vs. post**  OR 1.34 (0.83, 2.17)  **Post vs. comparison**  OR 1.03 (0.63, 1.67) |
|  |  |  | Adherence with guidelines regarding treating sitting balance |  | 36/151  (23.8%) | 25/143  (17.5%) | 34/135  (25.2%) | **Pre vs. post**  OR 0.68 (0.38, 1.20)  **Post vs. comparison**  OR 0.63 (0.35, 1.12) |
|  |  |  | Adherence with guidelines regarding treating standing balance |  | 78/151  (51.7%) | 75/143  (52.5%) | 81/135  (60.0%) | **Pre vs. post**  OR 1.03 (0.65, 1.63)  **Post vs. comparison**  OR 0.74 (0.46, 1.18) |
|  |  |  | Adherence with guidelines regarding functional electrical stimulation for the lower extremity |  | 1/151  (0.7%) | 1/143  (0.7%) | 1/135  (0.7%) | **Pre vs. post**  OR 1.06 (0.07, 17.05)  **Post vs. comparison**  OR 0.94 (0.06, 15.24) |
|  |  |  | Adherence with guidelines regarding walking practice |  | 103/647  (15.9%) | 108/276  (39.1%) | 87/265  (32.8%) | **Pre vs. post**  OR 3.40 (2.46, 4.68)  **Post vs. comparison**  OR 1.32 (0.92, 1.87) |
|  |  |  | Adherence with guidelines regarding treadmill walking practice |  | 4/151  (2.7%) | 2/143  (1.4%) | 7/135  (5.2%) | **Pre vs. post**  OR 0.52 (0.09, 2.89)  **Post vs. comparison**  OR 0.26 (0.05, 1.27) |
|  |  |  | Adherence with guidelines regarding treating upper extremity range of motion |  | 82/647  (12.7%) | 59/276  (21.4%) | 67/265  (25.3%) | **Pre vs. post**  OR 1.87 (1.29, 2.71)  **Post vs. comparison**  OR 0.80 (0.54, 1.20) |
|  |  |  | Adherence with guidelines regarding interventions to prevent shoulder pain |  | 162/647  (25.0%) | 71/276  (25.7%) | 56/265  (21.1%) | **Pre vs. post**  OR 1.04 (0.75, 1.43)  **Post vs. comparison**  OR 1.29 (0.87, 1.93) |
|  |  |  | Adherence with guidelines regarding task specific training i.e. self-care |  | 187/647  (28.9%) | 113/276  (40.9%) | 115/265  (43.4%) | **Pre vs. post**  OR 1.71 (1.27, 2.29)  **Post vs. comparison**  OR 0.90 (0.64, 1.27) |
|  |  |  | Adherence with guidelines regarding techniques to reduce hand oedema |  | 11/151  (7.3%) | 8/143  (5.6%) | 12/135  (8.9%) | **Pre vs. post**  OR 0.75 (0.29, 1.93)  **Post vs. comparison**  OR 0.61 (0.24, 1.54) |
|  |  |  | Adherence with guidelines regarding ice/heat or soft tissue massage for the shoulder |  | 2/151  (1.3%) | 4/143  (2.8%) | 7/135  (5.2%) | **Pre vs. post**  OR 2.14 (0.39, 11.89)  **Post vs. comparison**  OR 0.53 (0.15, 1.84) |
|  |  |  | Adherence with guidelines regarding functional electrical stimulation for the upper extremity |  | 3/151  (2.0%) | 2/143  (1.4%) | 2/135  (1.5%) | **Pre vs. post**  OR 0.70 (0.12, 4.25)  **Post vs. comparison**  OR 0.94 (0.13, 6.79) |
|  |  |  | Adherence with guidelines regarding the provision of education regarding handling arm or shoulder |  | 57/647  (8.8%) | 26/276  (9.4%) | 27/265  (10.2%) | **Pre vs. post**  OR 1.08 (0.66, 1.75)  **Post vs. comparison**  OR 0.92 (0.52, 1.62) |
|  |  |  | Adherence with guidelines regarding upper extremity constraint induced therapy |  | 7/151  (4.6%) | 1/143  (0.7%) | 6/135  (4.4%) | **Pre vs. post**  OR 0.14 (0.02, 1.19)  **Post vs. comparison**  OR 0.15 (0.02, 1.27) |
|  |  |  | Adherence with guidelines regarding visual imagery to enhance arm recovery |  | 4/151  (2.7%) | 9/143  (6.3%) | 7/135  (5.2%) | **Pre vs. post**  OR 2.47 (0.74, 8.20)  **Post vs. comparison**  OR 1.23 (0.44, 3.40) |
| Semin-Goossens et al. 2003 | 2670 | Grol’s 5-step Implementation | Compliance with completing falls incident report form**  (Internal medicine ward) | Falls incident report | N/R  (40-50%) | N/R  (52%) | N/A | **Pre vs. post**  p=NR *^α^* |
|  |  |  | Compliance with completing falls incident report form  (Neurology ward) |  | N/R  (40-50%) | N/R  (60%) |  | **Pre vs. post**  p=NR *^α^* |
| White et al. 2020 | N/R | KTA | Compliance with using a surgical safety checklist** | Questionnaire | 66/324  (20%) | 103/183  (56%) | N/A | **Pre vs. post**  OR 5.03 (3.38, 7.49) |
|  |  |  | Adherence to verification of patient identification |  | 268/324  (83%) | 166/183  (91%) | N/A | **Pre vs. post**  OR 2.04 (1.15, 3.63) |
|  |  |  | Adherence to risk assessment for difficult intubation* |  | 160/324  (49%) | 144/183  (79%) | N/A | **Pre vs. post**  OR 3.78 (2.50, 5.73) |
|  |  |  | Adherence to risk assessment for blood loss |  | 220/324  (68%) | 161/183  (88%) | N/A | **Pre vs. post**  OR 3.46 (2.09, 5.72) |
|  |  |  | Adherence to use of pulse oximetry |  | 239/324  (74%) | 171/183  (93%) | N/A | **Pre vs. post**  OR 5.07 (2.68, 9.57) |
|  |  |  | Adherence to antibiotic administration |  | 189/324  (58%) | 176/183  (96%) | N/A | **Pre vs. post**  OR 17.96 (8.18, 39.45) |
|  |  |  | Adherence to surgical counting (needles, swabs and instruments) |  | 183/324  (56%) | 162/183  (89%) | N/A | **Pre vs. post**  OR 5.94 (3.59, 9.85) |
| **Determinant Frameworks** | | | | | | | | |
| Byrnes  et al. 2018 | 155 | i-PARiHS | Upgrade to full ward diet  (or appropriate texture modified diet) day of surgery of post-op day 1.  Diet upgrades delivered as intended (fidelity – *intervention delivered as intended)* ** | Medical record audit | N/R | 16/27  (59%) | N/A | **Pre vs. post**  P = NR *^α^* |
| Garrido et al. 2021 | 72 | CFIR | Global adherence with 12 indicators of non-pharmacological delirium prevention ** | Monitored by a trained external evaluator | 428/735  (58.2%) | 561/720  (77.9%) | N/A | **Pre vs. post (training)**  OR 2.53 (2.01, 3.18) |
|  |  |  |  |  | 428/735  (58.2%) | 492/651  (75.6%) | N/A | **Pre vs. post (6 months)**  OR 2.22 (1.76, 2.80) |
| Gu et al. 2020 | 142 | i-PARiHS | Assessment of nutritional status during nutritional risk screening | On-site observations  “Yes / no” | 95/96  (99.0%) | 188/189  (99.5%) | N/A | **Pre vs. post**  OR 1.98 (0.12, 31.99) |
|  |  |  | Assessment of nutritional status stability during nutritional risk screening |  | 78/96  (81.3%) | 183/189  (96.8%) | N/A | **Pre vs. post**  OR 7.04 (2.69, 18.40) |
|  |  |  | Assessment of nutritional status deterioration during nutritional risk screening |  | 82/96  (85.4%) | 182/189  (96.3%) | N/A | **Pre vs. post**  OR 4.44 (1.73, 11.41) |
|  |  |  | Assess impact of diseases on nutritional status during nutritional risk screening |  | 96/96  (100%) | 186/189  (98.4%) | N/A | **Pre vs. post**  OR 0.28 (0.01, 5.40) |
|  |  |  | Nutritional risk screening was completed within 24 hours of admission | Medical record review | 82/82  (100%) | 189/189  (100%) | N/A | **Pre vs. post**  OR - Not estimable |
|  |  |  | Nutritional risk screening completed once a week |  | 94/94  (100%) | 174/189  (92.1%) | N/A | **Pre vs. post**  OR 0.06 (0.00, 1.01) |
|  |  |  | Nutritional assessment among infants with moderate risk and above * |  | 0*/96*  (0%) | 174/189  (92.1%) | N/A | **Pre vs. post**  OR 2172.81  (128.59, 36713.11) |
|  |  |  | Assess medical history during nutritional assessment |  | 92/92  (100%) | 185/189  (97.9%) | N/A | **Pre vs. post**  OR 0.22 (0.01, 4.18) |
|  |  |  | Assess nutritional history during nutritional assessment |  | 76/102  (74.5%) | 187/189  (98.9%) | N/A | **Pre vs. post**  OR 31.99 (7.41, 138.11) |
|  |  |  | Assess diet history during nutritional assessment |  | 64/96  (66.7%) | 189/189  (100%) | N/A | **Pre vs. post**  OR 190.97  (11.53, 3163.43) |
|  |  |  | Assess medication history during nutritional assessment |  | 95/96  (99.0%) | 189/189  (100%) | N/A | **Pre vs. post**  OR 5.95 (0.24, 147.51) |
|  |  |  | Do a physical examination during nutritional assessment |  | 81/96  (84.4%) | 187/189  (98.9%) | N/A | **Pre vs. post**  OR 17.31 (3.87, 77.47) |
|  |  |  | Check clinical laboratory result during nutritional assessment |  | 1/96  (1.0%) | 161/189  (85.2%) | N/A | **Pre vs. post**  OR 546.25  (73.14, 4079.73) |
|  |  |  | Assess feeding difficulties during nutritional assessment |  | 28/96  (29.2%) | 188/189  (99.5%) | N/A | **Pre vs. post**  OR 456.57  (60.94, 3420.67) |
|  |  |  | Assess feeding difficulties risk factors during nutritional assessment |  | 7/96  (7.3%) | 184/189  (97.4%) | N/A | **Pre vs. post**  OR 467.89  (144.46, 1515.39) |
|  |  |  | Use weight for age / height for age / weight for height and growth curve during nutritional assessment |  | 0/*96*  (0%) | 132/189  (69.8%) | N/A | **Pre vs. post**  OR 444.74  (27.15, 7285.57) |
|  |  |  | Summary and intervention plan developed after nutritional assessment ** |  | 0/*96*  (0%) | 159/189  (84.1%) | N/A | **Pre vs. post**  OR 1009.30  (61.02, 16694.78) |
| Peel et al. 2021 | 690 | i-PARiHS | Proportion of screening items complete in routine screening documentation (data items) * | Medial record Audit | 384/630  (61%) | 8922/9030  (99%) | N/A | **Pre vs. post**  OR 52.92 (41.29, 67.83) |
|  |  |  | Screening for risks of harm within 24 hours of admission (data items) |  | 392/630  62% | 7854/9030  87% | N/A | **Pre vs. post**  OR 4.05 (3.41, 4.82) |
| Savoie et al. 2019 | 70 | Active Implementation Frameworks | Assessment of pain on admission using ISCIPBDS 2.0 * | Chart audit | New assessment process post implementation | N/R  (85%) | N/A | **Pre vs. post**  *N/A* |
|  |  |  | Patients concerns and expectations documented on admission |  | N/R | N/R  (74%) | N/A | **Pre vs. post**  *P=N/R ^α^* |
|  |  |  | Initial discipline specific assessments documented |  | N/R | N/R  (84%) | N/A | **Pre vs. post**  *P=N/R ^α^* |
|  |  |  | Documentation of pain intensity at admission |  | N/R  (43%) | N/R  (93%) | N/A | **Pre vs. post**  *P=N/R ^α^* |
|  |  |  | Date of pain onset documented |  | N/R  (4.5%) | N/R  (80%) | N/A | **Pre vs. post**  *P=N/R ^α^* |
|  |  |  | Documentation of type of pain (nociceptive vs neuropathic) |  | N/R  (50%) | N/R  (100%) | N/A | **Pre vs. post**  *P=N/R ^α^* |
|  |  |  | Development of an Inter-professional pain treatment plan ** |  | New process implemented | N/R  (74%) | N/A | **Pre vs. post**  *N/A* |
|  |  |  | For patients with a documented IPTP plan discussed within 7 days |  | New process implemented | N/R  (92%) | N/A | **Pre vs. post**  *N/A* |
|  |  |  | Documented formal pain education |  | N/R  (12%) | N/R  (74%) | N/A | **Pre vs. post**  *P=N/R ^α^* |
|  |  |  | Documentation of daily pain intensity throughout admission |  | N/R | N/R  (67%) | N/A | **Pre vs. post**  *P=N/R ^α^* |
|  |  |  | Pain discharge plan |  | N/R  (40%) | N/R  (74%) | N/A | **Pre vs. post**  *P=N/R ^α^* |
| Scovil  et al. 2019 | 2371 | Active Implementation Frameworks | Completion rates of inter-professional pressure injury risk factor determination* | Medical record audit | New assessment process post implementation | 367/982  (37%) | N/A | **Pre vs. post**  *N/A* |
|  |  |  | Completion rates inter-professional prevention plan |  | New assessment process post implementation | 283/982  (29%) |  | **Pre vs. post**  *N/A* |
|  |  |  | Delivery of educational materials** | Medical record audit, attendance logs & patient self-report* | 52/123  (42%) | 720/969  (74%) |  | **Pre vs. post**  OR 3.95 (2.68, 5.81) |
|  |  |  | Delivery of unstructured individualized education |  | 131/191  (69%) | 831/1007  (83%) |  | **Pre vs. post**  OR 2.16 (1.53, 3.06) |
|  |  |  | Patient attendance to structured class-based education |  | 64/148  (43%) | 500/992  (50%) |  | **Pre vs. post**  OR 1.33 (0.94, 1.89) |
| Stevens et al. 2014 | 2564 | PARiHS | Children per unit whose pain was assessed with any pain assessment* | Medical record audit | 1297/1914  (67.8%) | 450/485  (92.8%) | 418/479  (87.3%) | **Pre vs. post**  OR 6.12 (4.28, 8.74)  **Post vs. comparison**  OR 1.88 (1.21, 2.90) |
|  |  |  | *Sustainability data available for subset of original units* |  | 675/956  (70.6%) | 184/263  (70.0%) |  | **Pre vs. 24 months post**  OR 0.97 (0.72, 1.31) |
|  |  |  |  |  | 675/956  (70.6%) | 183/239  (76.6%) |  | **Pre vs. 36 months post**  OR 1.36 (0.98, 1.89) |
|  |  |  |  |  | 675/956  (70.6%) | 184/240  (76.7%) |  | **Pre vs. 48 months post**  OR 1.37 (0.98, 1.90) |
|  |  |  | Children per unit whose pain was assessed with any validated pain assessment |  | 501/1914  (26.2%) | 290/485  (59.8%) | 199/479  (41.5%) | **Pre vs. post**  OR 4.19 (3.41, 5.17)  **Post vs. comparison**  OR 2.09 (1.62, 2.71) |
|  |  |  | *Sustainability data available for subset of original units* |  | 293/954  (30.7%) | 87/263  (33.1%) |  | **Pre vs. 24 months post**  OR 1.12 (0.83, 1.49) |
|  |  |  |  |  | 293/954  (30.7%) | 95/239  (39.8%) |  | **Pre vs. 36 months post**  OR 1.49 (1.11, 2.00) |
|  |  |  |  |  | 293/954  (30.7%) | 94/240  (39.2%) |  | **Pre vs. 48 months post**  OR 1.45 (1.08, 1.95) |
|  |  |  | Children per unit who underwent any painful procedure |  | 1524/1914  (79.6%) | 402/485  82.9% | 384/479  (80.2%) | **Pre vs. post**  OR 1.24 (0.95, 1.61)  **Post vs. comparison**  OR 1.20 (0.86, 1.66) |
|  |  |  | *Sustainability data available for subset of original units* |  | 778/1523  (51.1%) | 228/263  (86.7%) |  | **Pre vs. 24 months post**  OR 6.24 (4.31, 9.03) |
|  |  |  |  |  | 778/1523  (51.1%) | 196/239  (82.0%) |  | **Pre vs. 36 months post**  OR 4.36 (3.09, 6.16) |
|  |  |  |  |  | 778/1523  (51.1%) | 191/240  (79.6%) |  | **Pre vs. 48 months post**  OR 3.73 (2.68, 5.19) |
|  |  |  | Children per unit who underwent any painful procedure with any pain management strategy** |  | 1163/1524  (76.3%) | 322/402  80.1% | 315/384  (82.0%) | **Pre vs. post**  OR 1.25 (0.95, 1.64)  **Post vs. comparison**  OR 0.88 (0.62, 1.26) |
|  |  |  | *Sustainability data available for subset of original units* |  | 580/777  (74.6%) | 173/228  (75.9%) |  | **Pre vs. 24 months post**  OR 1.07 (0.76, 1.51) |
|  |  |  |  |  | 580/777  (74.6%) | 139/196  (70.9%) |  | **Pre vs. 36 months post**  OR 0.83 (0.58, 1.17) |
|  |  |  |  |  | 580/777  (74.6%) | 130/191  (68.1%) |  | **Pre vs. 48 months post**  OR 0.72 (0.51, 1.02) |
|  |  |  | Children per unit who underwent any painful procedure and received analgesia |  | 1001/1524  (65.7%) | 277/402  (68.9%) | 278/384  (72.4%) | **Pre vs. 12 months post**  OR 1.16 (0.91, 1.47)  **Post vs. comparison**  OR 0.84 (0.62, 1.15) |
|  |  |  | *Sustainability data available for subset of original units* |  | 532/778  (68.4%) | 161/228  70.6% |  | **Pre vs. 24 months post**  OR 1.11 (0.80, 1.53) |
|  |  |  |  |  | 532/778  (68.4%) | 137/196  69.9% |  | **Pre vs. 36 months post**  OR 1.07 (0.76, 1.51) |
|  |  |  |  |  | 532/778  (68.4%) | 119/191  62.3% |  | **Pre vs. 48 months post**  OR 0.76 (0.55, 1.06) |
|  |  |  | Children per unit who underwent any painful procedure and received a physical strategy |  | 235/1526  (15.4%) | 89/403  (22.1%) | 100/385  (26.0%) | **Pre vs. post**  OR 1.56 (1.18, 2.05)  **Post vs. comparison**  OR 0.81 (0.58, 1.12) |
|  |  |  | *Sustainability data available for subset of original units* |  | 90/776  (11.6%) | 46/93  (49.5%) |  | **Pre vs. 24 months post**  OR 7.46 (4.70, 11.84) |
|  |  |  |  |  | 90/776  (11.6%) | 18/196  (9.2%) |  | **Pre vs. 36 months post**  OR 0.77 (0.45, 1.31) |
|  |  |  |  |  | 90/776  (11.6%) | 38/191  (19.9%) |  | **Pre vs 48 months post**  OR 1.89 (1.25, 2.87) |
|  |  |  | Children per unit who underwent any painful procedure and received psychological strategy |  | 243/1528  (15.9%) | 68/402  (16.9%) | 78/384  (20.3%) | **Pre vs. post**  OR 1.08 (0.80, 1.45)  **Post vs. comparison**  OR 0.80 (0.56, 1.15) |
|  |  |  | *Sustainability data available for subset of original units* |  | 95/779  (12.2%) | 44/228  (19.3%) |  | **Pre vs. 24 months post**  OR 1.72 (1.16, 2.55) |
|  |  |  |  |  | 95/779  (12.2%) | 11/196  (5.6%) |  | **Pre vs. 36 months post**  OR 0.43 (0.22, 0.82) |
|  |  |  |  |  | 95/779  (12.2%) | 33/191  (17.3%) |  | **Pre vs 48 months post**  OR 1.50 (0.98, 2.32) |
| Sving  et al. 2016 | 506  (+259) | PARiHS | Patients for whom prevention measures were implemented** | Medical record audit, bedside assessment and observation of preventive activities at the bedside | 18/251  (7%) | 45/253  (18%) | *N/A* | **Pre vs. post**  OR 2.80 (1.57, 4.99) |
|  |  |  |  |  | 18/251  (7%) | N/A | *52/259*  *(20%)* | **Pre vs. long term**  OR 3.25 (1.84, 5.74) |
|  |  |  | Three or more pressure injury prevention activities |  | 4/251  (1.6%) | 13/255  (5.2%) | N/A | **Pre vs. post**  OR 3.32 (1.07, 10.32) |
|  |  |  | Skin assessments documented within 24 hours of admission* | Medical record audit | 200/251  (79%) | 229/255  (90%) | N/A | **Pre vs. post**  OR 2.25 (1.35, 3.74) |
|  |  |  | Risk assessments documented within 24 hours of admission |  | 151/251  (60%) | 211/255  (84%) | N/A | **Pre vs. post**  OR 3.18 (2.10, 4.79) |
| Taylor et al. 2013 | 367 | Theoretical Domains Framework Implementation (TDFI) approach | Use of pH as first line check of nasogastric tube placement*  *(note: re-assessment following an intervention)* | Medical record audit | All 3 hospitals combined  20/136  (14.7%) | All 3 hospitals combined  75/132  (56.8%) | 16/46  33.3% | **Pre vs. post**  OR 7.63 (4.25, 13.72)  **Post vs. comparison**  OR 2.47 (1.23, 4.96) |
|  |  |  | X-ray *NOT USED* as first line check of nasogastric tube placement |  | All 3 hospitals combined  61/136  (45%) | All 3 hospitals combined  101/132  (77%) | 37/46  (80%) | **Pre vs. post**  OR 4.01 (2.37, 6.78)  **Post vs. comparison**  OR 0.79 (0.34, 1.82) |
|  |  |  | Nasogastric tube NOT placed in radiology |  | All 3 hospitals combined  119/136  88% | All 3 hospitals combined  128/132  97% | 46/46  100% | **Pre vs. post**  OR 4.57 (1.50, 13.97)  **Post vs. comparison**  OR 0.31 (0.02, 5.81) |
|  |  |  | Practice of first line check of nasogastric tube placement not documented |  | All 3 hospitals combined  24/136  (18%) | All 3 hospitals combined  22/132  (17%) | 21/46  (45.7%) | **Pre vs. post**  OR 0.93 (0.49, 1.76)  **Post vs. comparison**  OR 0.24 (0.11, 0.50) |
| Thomas et al. 2016 | 313 | TDF | Patients identified as being at risk of falls prior to discharge* | Medical record audit | 10/159  (6.3%) | 146/154  (94.8%) | N/A | **Pre vs. post**  OR 271.93  (104.40, 708.27) |
|  |  |  | Documentation of clinical handover at discharge*** |  | 109/159  (68.6%) | 140/154  (90.9%) | N/A | **Pre vs. post**  OR 4.59 (2.41, 8.73) |
|  |  |  | Quality of the documented clinical handover (meeting 5 criteria) |  | 38/109  (34.9%) | 130/140  (92.9%) | N/A | **Pre vs. post**  OR 24.29 (11.42, 51.64) |
| Tian et al. 2017 |  | PARIHS | Compliance with the evidence-based practice (EBP) using a "nursing quality checklist of inpatient Cancer Related Fatigue management" ** | Checklist – 6 items related to Screening and Assessment and another 5 relating to interventions | n/a new intervention | Proportions for unit A and unit B provided for 11 items of checklist  I.e. Screening and assessment item 1  Unit A  N/R  100%  Unit B  N/R  100% | N/A | **Pre vs. post**  p=N/R *^α^* |
| White et al. 2019 | N/R | CFIR | Compliance with using a surgical safety checklist** | Questionnaire | 169/543  (31.1%) | 158/178  (88.8%) | N/A | **Pre vs. post (4 months)**  OR 17.48 (10.61, 28.81) |
|  |  |  |  |  | 169/543  (31.1%) | 86/100  (86%) | N/A | **Pre vs. post (12–18 months)**  OR 13.59 (7.51, 24.61) |
|  |  |  | Adherence to verification of patient identification, type and site of surgery |  | 495/543  (91.2%) | 170/178  (95.5%) | N/A | **Pre vs. post (4 months)**  OR 2.06 (0.96, 4.44) |
|  |  |  |  |  | 495/543  (91.2%) | 96/100  (96%) | N/A | **Pre vs. post (12-18 months)**  OR 2.33 (0.82, 6.61) |
|  |  |  | Adherence to risk assessment for difficult intubation* |  | 426/543  (78.5%) | 160/178  (89.9%) | N/A | **Pre vs. post (4 months)**  OR 2.44 (1.44, 4.14) |
|  |  |  |  |  | 426/543  (78.5%) | 89/100  (89%) | N/A | **Pre vs. post (12-18 months)**  OR 2.22 (1.15, 4.29) |
|  |  |  | Adherence to risk assessment for blood loss |  | 448/543  (82.5%) | 147/178  (82.6%) | N/A | **Pre vs. post (4 months)**  OR 1.01 (0.64, 1.57) |
|  |  |  |  |  | 448/543  (82.5%) | 85/100  (85%) | N/A | **Pre vs. post (12-18 months)**  OR 1.20 (0.66, 2.17) |
|  |  |  | Adherence to use of pulse oximetry |  | 524/543  (96.5%) | 175/178  98.3% | N/A | **Pre vs. post (4 months)**  OR 2.12 (0.62, 7.23) |
|  |  |  |  |  | 524/543  (96.5%) | 99/100  (99%) | N/A | **Pre vs. post (12-18 months)**  OR 3.59 (0.48, 27.12) |
|  |  |  | Adherence to antibiotic administration before surgical site incision |  | 280/543  (51.6%) | 157/178  (88.2%) | N/A | **Pre vs. post (4 months)**  OR 7.02 (4.32, 11.41) |
|  |  |  |  |  | 280/543  (51.6%) | 87/100  (87%) | N/A | **Pre vs. post (12-18 months)**  OR 6.29 (3.43, 11.53) |
|  |  |  | Adherence to surgical counting (needles, swabs and instruments) before and after surgery |  | 334/543  (61.5%) | 166/178  (93.3%) | N/A | **Pre vs. post (4 months)**  OR 8.66 (4.70, 15.94) |
|  |  |  |  |  | 334/543  (61.5%) | 90/100  (90%) | N/A | **Pre vs. post (12-18 months)**  OR 5.63 (2.87, 11.07) |
| **>1 Framework from across categories** | | | | | | | | |
| Bosch et al 2019 | 1943 | TDF &  Model of Diffusion of Innovations in Service Organisations | Appropriate post-traumatic amnesia screening using a valid tool* | Retrospective Chart Audit | N/A | 117/893  (13%) | 12/1050  (1.1%) | **Post vs. comparison**  OR 13.04 (7.15, 23.80) |
|  |  |  | PTS screening tool |  | N/A | 152/893  (17%) | 15/1050  (1.4%) | **Post vs. comparison**  OR 14.15 (8.26, 24.26) |
|  |  |  | Memory clinical Ax (questions included in clinical assessment) |  | N/A | 303/893  (34%) | 272/1050  (26%) | **Post vs. comparison**  OR 1.47 (1.21, 1.79) |
|  |  |  | CT scan – clinical criteria |  | N/A | 352/491  (72%) | 337/494  (68%) | **Post vs. comparison**  OR 1.18 (0.90, 1.55) |
|  |  |  | CT scan (all) |  | N/A | 446/893  (50%) | 458/1050  (44%) | **Post vs. comparison**  OR 1.29 (1.08, 1.54) |
|  |  |  | Provision of written information at discharge |  | N/A | 160/785  (20%) | 175/944  (19%) | **Post vs. comparison**  OR 1.12 (0.89, 1.43) |
| Roberts et al. 2019 | 207 | KTA & TDF | Mealtimes interrupted by nursing procedure | Observations of mealtime using a data collection schedule | 66/423  16% | 85/400  21% | N/A | **Pre vs. post**  OR 1.46 (1.02, 2.08) |
|  |  |  | Mealtimes interrupted by ward round |  | 23/423  5% | 18/400  5% | N/A | **Pre vs. post**  OR 0.82 (0.44, 1.54) |
|  |  |  | Mealtimes interrupted by medication round |  | 14/423  3% | 58/400  15% | N/A | **Pre vs. post**  OR 4.95 (2.72, 9.04) |
|  |  |  | Mealtimes interrupted by patient being taken off ward |  | 5/423  1% | 3/400  0.01% | N/A | **Pre vs. post**  OR 0.63 (0.15, 2.66) |
|  |  |  | Mealtimes interrupted by pathology |  | 4/423  1% | 7/400  2% | N/A | **Pre vs. post**  OR 1.87 (0.54, 6.42) |
|  |  |  | Patient ready to receive their meal tray when meal tray delivered ** |  | 323/423  76% | 337/400  84% | N/A | **Pre vs. post**  OR 1.66 (1.17, 2.35) |
| Robertson et al. 2018 | 130 | KTA & TDF | Proportion prescribed a full diet on operative notes** | Medical record audit | 0/43  (0%) | 23/28  82% | N/A | **Pre vs. post**  OR 371.73 (19.7, 7019.0) |
| Romney et al. 2019 | 621 | TDF &  KTA | Assessment of gait speed (4MWT) at Initial examination* | Medical record audit | 0/50  (0%) | 41/79  (52%) | N/A | **Pre vs. post (8 month)**  OR 108.87 (6.49, 1826.15) |
|  |  |  | Assessment of gait speed (4MWT) at discharge |  | 0/91  (0%) | 66/93  (71%) | N/A | **Pre vs. post (8 month)**  OR 442.53 (26.52, 7384.73) |

OR>1: Favours post-implementation, SMD>0: Favours post-implementation, N/R: Not Reported, N/A: Not Applicable, ^α^: Insufficient data provided to calculate odds ratio, *Screening and assessment process of care outcome used in meta-analysis, **Providing recommended care process of care outcome used in meta-analysis, ***other process of care outcome used in meta-analysis

***Supplemental Table 12: All patient outcomes***

| **Study / Year** | **Patients**  **n** | **Theoretical Implementation Framework** | **Patient Outcome** | **How was**  **outcome measured** | **Pre-impl.** | **Post-impl.** | | **Comparison Unit** | **Results:** |
| --- | --- | --- | --- | --- | --- | --- | --- | --- | --- |
| **Process Models** | | | | | | | | | |
| Azar et al. 2019 | N/A | Agile Implementation Model | Rate Central line associated blood stream infections (CLABI) per 1000 central line days* | Audit of routinely collected surveillance data and Medical record audit | 1.76 | 1.24 | |  | **Pre vs. post**  *p=0.011* ^α^ |
|  |  |  | Rate of catheter acquired UTI per 1000 Foley catheter days |  | 1.91 | 1.74 | |  | **Pre vs. post**  *p=0.977* ^α^ |
|  |  |  | Rate of Clostridium difficile (c-diff) infections per 10 000 patient days |  | 13.27 | 8.83 | |  | **Pre vs. post**  *p<0.001* ^α^ |
|  |  |  | Overall harm total number per month |  | 51.1 | 36.1 | |  | **Pre vs. post**  *p<0.001* ^α^ |
| Cody et al. 2021 | 294 | JBI evidence implementation framework | Rate of hospital acquired delirium* | Audit routinely collected surveillance data | 8.1 per 1,000 separations | 7.1 per 1,000 separations | | *1.7 per 1,000 separations* | **Pre vs. post**  N/R |
| Rattray et al. 2021 | 64 | KTA | Time (in hours) to first dietary intake* | Direct observation supplemented where necessary by verbal clarification with nurses / patients | n = 30  Median (IQR)  15.7 (7.4-22.5)  Estimated Mean (SD)(40)  15.2 (11.75) | n = 34  Median (IQR)  4.9 (3.7-14.2)  Estimated Mean (SD)(40)  7.6 (8.13) | | N/A | **Pre vs. post**  SMD -0.75 (-1.26, -0.24) |
|  |  |  | Time (in hours) to first solid diet intake |  | 86.1 (60.1-104)  Estimated Mean (SD)(40)  83.4 (34.17) | 69.2 (46.1-115.5)  Estimated Mean (SD)(40)  76.93 (53.71) | | N/A | **Pre vs. post**  SMD -0.14 (-0.63, 0.35) |
|  |  |  | Nutrition (any type) intake ≤ 6 hours*  *DID NOT receive nutrition intake (any type) ≤ 6 hours* |  | 5/30  17%  25/30  83% | 20/34  59%  14/34  41% | | N/A | **Pre vs. post**  OR 7.14 (2.20, 23.21)  OR 0.14 (0.04, 0.45) |
|  |  |  | Nutrition (any type) intake ≤ 24 hours  *DID NOT receive nutrition intake (any type) ≤ 24 hours* |  | 24/30  80%  6/30  20% | 32/34  94%  2/34  6% | | N/A | **Pre vs. post**  OR 4.00 (0.74, 21.58)  OR 0.25 (0.05, 1.35) |
|  |  |  | Solid oral intake ≤ 24 hours  *DID NOT receive solid oral intake ≤ 24 hours* |  | 4/30  13%  26/30  87% | 1/34  3%  33/34  97% | | N/A | **Pre vs. post**  OR 0.20 (0.02, 1.87)  OR 5.08 (0.53, 48.21) |
| Reynolds et al. 2018 | N/A | Grol and Wensing | Rate of hospital acquired CLABSIs per 1000 catheter days | Audit routinely collected surveillance data | 2.81 | 1.12 | | N/A | **Pre vs. post**  *p=0.031* ^α^ |
| Semin-Goossens et al. 2003 | 2670 | Grol’s 5-step Implementation | Rate of falls (internal medicine) per 1000 bed days* | Audit medical record | ‘Average’ 9 | ‘Average’ 7 | | N/A | **Pre vs. post**  p=N/R ^α^ |
|  |  |  | Rate of falls (neurology unit) per 1000 bed days |  | ‘Average’ 16 | ‘Average’ 16.5 | |  | **Pre vs. post**  p=N/R ^α^ |
| **Determinant Frameworks** | | | | | | | | | |
| Byrnes et al. 2018 | 155 | i-PARiHS | Proportion of patients that DID NOT receive early nutrition* | Medical record audit | 21/45  (46.7%) | 10/47  (21.3%) | | 7/36  (19.4%) | **Pre vs. post**  OR 0.31 (0.12 - 0.77)  **Post vs. comparison**  OR 1.12 (0.38, 3.30) |
|  |  |  | Antiemetic therapy requirements to postoperative day 3  n, (%) |  | 29/45  (67%) | 30/47  (65%) | | 18/36  (51%) | **Pre. vs post.**  OR 0.97 (0.42 - 2.28)  **Post vs. comparison**  OR 1.76 (0.73 - 4.27) |
|  |  |  | Vomiting  n, (%) |  | 19/45  (42%) | 10/47  (21%) | | 7/36  (19%) | **Pre. vs post**  OR 0.37 (0.15 - 0.92)  **Post vs. comparison**  OR 1.12 (0.38 - 3.30) |
|  |  |  | Nasogastric tube (NGT) insertion  n, (%) |  | 8/45  (18%) | 6/47  (13%) | | 1/36  (3%) | **Pre. vs post**  OR 0.68 (0.21 - 2.13)  **Post vs. comparison**  OR 5.12 (0.59 - 44.61) |
|  |  |  | Diet downgrade to postoperative day 3  n, (%) |  | 11/45  (24%) | 8/47  (17%) | | 2/36  (6%) | **Pre. vs post**  OR 0.63 (0.23 - 1.76)  **Post vs. comparison**  OR 3.49 (0.69 - 17.56) |
|  |  |  | Postoperative surgical complications surgical site infection n (%)* |  | 6/45  (13%) | 1/47  (2%) | | 1/36  (3%) | **Pre. vs post**  OR 0.14 (0.02 - 1.22)  **Post vs. comparison**  OR 0.76 (0.05 - 12.59) |
|  |  |  | Postoperative surgical complication ileus  n, (%) |  | 8/45  (18%) | 8/47  (17%) | | 2/36  (6%) | **Pre. vs post**  OR 0.95 (0.32 - 2.79)  **Post vs. comparison**  OR 3.49 (0.69 - 17.56) |
|  |  |  | Postoperative surgical complication anastomotic leak  n, (%) |  | 2/45  (4%) | 0/47  (0%) | | 1/36  (3%) | **Pre. vs post**  OR 0.18 (0.01 - 3.92)  **Post vs. comparison**  OR 0.25 (0.01 - 6.30) |
|  |  |  | Postoperative surgical complication reoperation  n, (%) |  | 6/45  (13%) | 2/47  (4%) | | 0/36  (0%) | **Pre. vs post**  OR 0.29 (0.06 - 1.51)  **Post vs. comparison**  OR 4.01 (0.19 - 86.18) |
| Garrido et al. 2020 | 72 | CFIR | Delirium incidence* | Validated tool – Confusion assessment method administered twice a day during the first 5 days of hospitalisation. | 5/25  (20%) | 4/23  (18%) | |  | **Pre vs. post**  OR 0.84 (0.20, 3.62) |
|  |  |  |  |  | 5/25  (20%) | 4/24  (16%) | |  | **Pre vs. post (6 month)**  OR 0.80 (0.19, 3.42) |
| Gu et al. 2020 | 142 | i-PARiHS | Time to initiation of enteral nutrition*  (in hours)  (Longer time = worse outcome) | Medical record review | n = 70  Mean (SD)  34.41 (26.58) | n = 72  25.21 (12.40) | | N/A | **Pre vs. post**  SMD -0.44 (-0.77, -0.11) |
|  |  |  | Length of stay  (in days)  (Longer time = worse outcome) |  | Mean (SD)  15.0 (9.45) | 17.1 (8.06) | | N/A | **Pre vs. post**  SMD 0.24 (-0.09, 0.57) |
|  |  |  | Weight gain during hospitalization  (in grams) |  | Median (IQR)  100(0-200) | 10 (-10 – 328) | | N/A | **Pre vs. post**  p = 0.507 |
|  |  |  | Weight gain adjusted by screening tool risk on nutritional status and growth score   - Low risk |  | Median (IQR)  0 (-160 – 0) | 0 (-100 – 400) | | N/A | **Pre vs. post**  p = 0.310 |
|  |  |  | - Medium Risk |  | Median (IQR)  100 (0 – 160) | 50 (-175 – 317) | | N/A | **Pre vs. post**  p = 0.383 |
|  |  |  | - High Risk |  | Median (IQR)  360 (-170 – 0) | 100 (-100 – 340) | | N/A | **Pre vs. post**  p = 0.458 |
| Scovil et al. 2019 | 2371 | “Active Implementation Frameworks” of the National Implementation Research Network (NIHR) | PI incidence* – any stage n, (%) | Medical record audit | 58/341  (17%) | 270/2030  (13.3%) | | N/A | **Pre vs. post**  OR 0.75 (0.55 - 1.02) |
|  |  |  | PIs stage 2 or greater n, (%) |  | 24/341  (7%) | 146/2030  (7.2%) | |  | **Pre vs. post**  OR 1.02 (0.65 - 1.60) |
| Stevens et al. 2014 | 640 | PARiHS | Pain intensity - mild, moderate or severe*  total n, (%) | Direct observation during routine, scheduled painful procedures | N/R | | 576/640 90% (total post-implementation intervention and control units) | | **Post vs. comparison**  p=0.032 ^α^ |
|  |  |  | Pain intensity – severe total n, (%) |  | N/R | | 122/640 19% (total post-implementation intervention and control units) | | **Post vs. comparison**  p=0.010 ^α^ |
| Sving et al. 2016 | 506 | PARiHS | Prevalence of categories pressure Ulcers 1–4 all patients*  n, (%) | Skin assessments  and medical record audit | 28/251  (11%) | 29/255  (11%) | | N/A | **Pre vs. post**  OR 1.02 (0.59, 1.77) |
|  |  |  | Prevalence of categories 2–4 PI all patients n, (%) |  | 15/251  (6%) | 22/255  (8.7%) | | N/A | **Pre vs. post**  OR 1.49 (0.75 - 2.93) |
|  |  |  | Unit-acquired pressure ulcer categories 1–4 all patients n, (%) |  | 25/297  (8.4%) | 23/255  (9%) | | N/A | **Pre vs. post**  OR 1.08 (0.60 - 1.95) |
| Tian et al. 2017 | 2014 | Ottowa Model of Research Use | CRF self-management ability – knowledge* unit A mean (SD) | Survey of patients with a 16-item Cancer Related Fatigue self-management scale  *(Higher scores = improved*  *Note: mean score relates to percentage of scores of 3 (to great extent) for each item on a 1-3 scale)* | n=50  34.74  (7.07) | n=44  38.70  (6.21) | | N/A | **Pre vs. post**  SMD -0.59 (-1.00, -0.17) |
|  |  |  | CRF self-management ability - knowledge unit B mean (SD) |  | n=55  35.11  (6.01) | n=55  41.05  (6.09) | |  | **Pre vs. post**  SMD -0.97 (1.40, -0.54) |
|  |  |  | CRF self-management ability - scale of attention unit A mean (SD) |  | 29.72  (4.05) | 35.41  (5.51) | |  | **Pre vs. post**  SMD -1.18 (-1.62, -0.74) |
|  |  |  | CRF self-management ability - scale of attention unit B mean (SD) |  | 33.51  (6.17) | 37.51  (5.70) | |  | **Pre vs. post**  SMD -0.67 (-1.08, -0.25) |
|  |  |  | CRF self-management ability - behaviour unit A mean (SD) |  | 31.00  (3.63) | 36.36  (4.91) | |  | **Pre vs. post**  SMD -1.24 (-1.69, -0.80) |
|  |  |  | CRF self-management ability - behaviour unit B  mean (SD) |  | 33.24  (4.57) | 37.42  (5.56) | |  | **Pre vs. post**  SMD -0.82 (-1.21, -0.40) |
| **>1 Framework Across Framework Categories** | | | | | | | | | |
| Bosch et al 2019 | 343 | Theoretical Domains Framework & Model of Diffusion of Innovations in Service Organisations | Anxiety  Hospital Anxiety and depression scale (HADS)*  *(Lower score = better outcome)* | Follow-up telephone interview  (4.3-10.7 months post ED presentation) |  | n = 125  Mean/SD  3.4 (3.58) | | n= 218  Mean/SD  4.3 (4.01) | **Post vs. Comparison**  **SMD** -0.24 (0.46, -0.02) |
|  |  |  | Post-concussion symptoms  Rivermead scale (RPQ-13)  *(Lower score = better outcome)* |  |  | n = 125  Mean/SD  4.7 (5.52) | | n= 218  Mean/SD  6.7 (8.68) | **Post vs. Comparison**  **SMD** -0.29 (-0.51, -0.07) |
|  |  |  | Post-concussion symptoms  Rivermead scale (RPQ-3)  *(Lower score = better outcome)* |  |  | n = 125  Mean/SD  0.90 (1.44) | | n= 218  Mean/SD  1.16 (1.83) | **Post vs. Comparison**  **SMD** -0.16 (-0.38, -0.06) |
|  |  |  | NOT returned to usual activities |  |  | 16/126  13% | | 41/218  19% | **Post vs. Comparison**  OR 1.59 (0.85, 2.97) |
|  |  |  | Health related QoL  SF6D  *(Higher score = better outcome)* |  |  | n = 123  Mean/SD  0.80 (0.13) | | n= 208  Mean/SD  0.78 (0.14) | **Post vs. Comparison**  SMD -0.15 (-0.37, 0.08) |
|  |  |  | Representation to ED for reason related to mTBI |  |  | 39/893  4.4% | | 25/1050  2.4% | **Post vs. Comparison**  OR 0.53 (0.32, 0.89) |
| Roberts et al. 2019 | 207 | KTA | Adequate energy intake*  *DID NOT receive adequate energy intake* | Observation of plates at end of each meal and amount of food consumed recorded. | 13/66  20%  53/66  80% | 27/61  44%  34/61  56% | | N/A | **Pre vs. post**  OR 3.24 (1.47, 7.13)  OR 0.31 (0.14, 0.68) |
|  |  |  | Adequate protein intake  DID NOT receive adequate protein intake |  | 16/66  24%  50/66  76% | 28/61  46%  33/61  54% | | N/A | **Pre vs. post**  OR 2.65 (1.25, 5.64)  OR 0.38 (0.18, 0.80) |
| Robertson et al. 2018 | 130 | KTA | Post op. day commenced full diet Median (range) | Audit medical records and hospital electronic foodservice management systemF | 4 (2-7)  *Estimated Mean (SD)*(40)  *4.25 (1.25)*  *(n=23)* | 0 (0-5)  *Estimated Mean (SD)*(40)  *1.25 (1.25)*  *(n=28)* | | N/A | **Pre vs. post**  SMD -2.36 [-3.09, -1.63) |
|  |  |  | Proportion who ***did not*** received a full diet on post op. day 0* n, (%) |  | 23/23  100% | 8/28  (28.6%) | |  | **Pre vs. post**  OR 0.01 (0.00, 0.16) |

N/R: Not Reported, N/A: Not Applicable, ^α^: Insufficient data provided to calculate odds ratio / SMD, OR<1 Favours post-implementation, SMD<0 favours post- implementation, *Primary patient outcome included where applicable in meta-analysis.

***Supplemental Figure 1: Studies targeting single verses multiply professional groups by framework category****.*

***Supplemental Figure 2: Summary plot risk of bias by framework category***

| 1. ***Process Models***   ***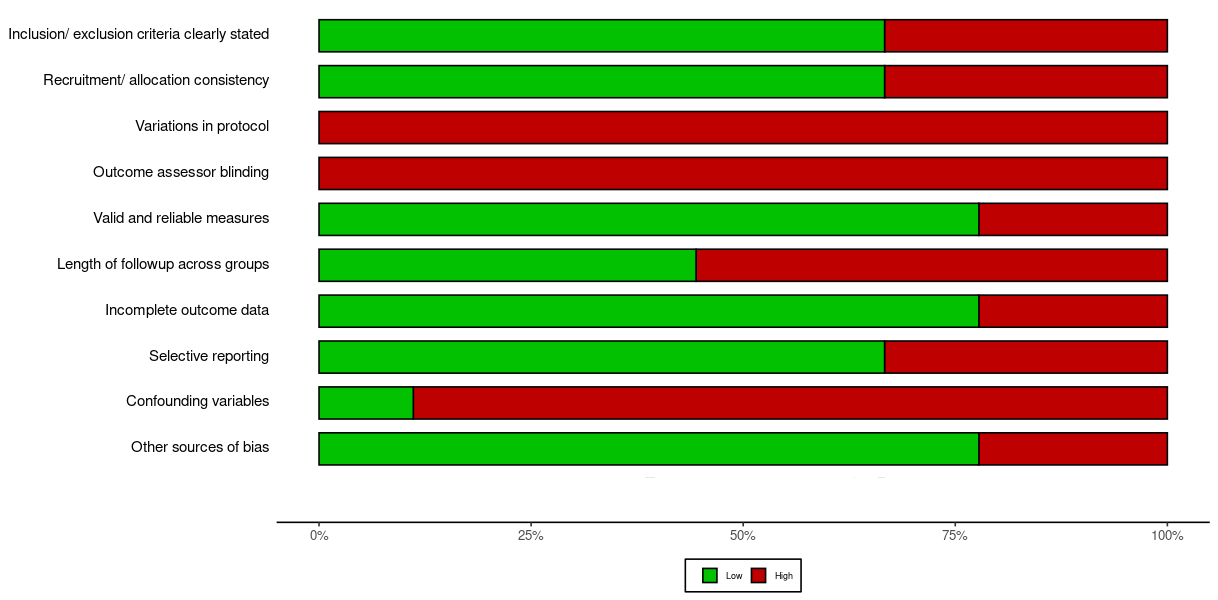*** | 1. ***Determinant Frameworks***   ***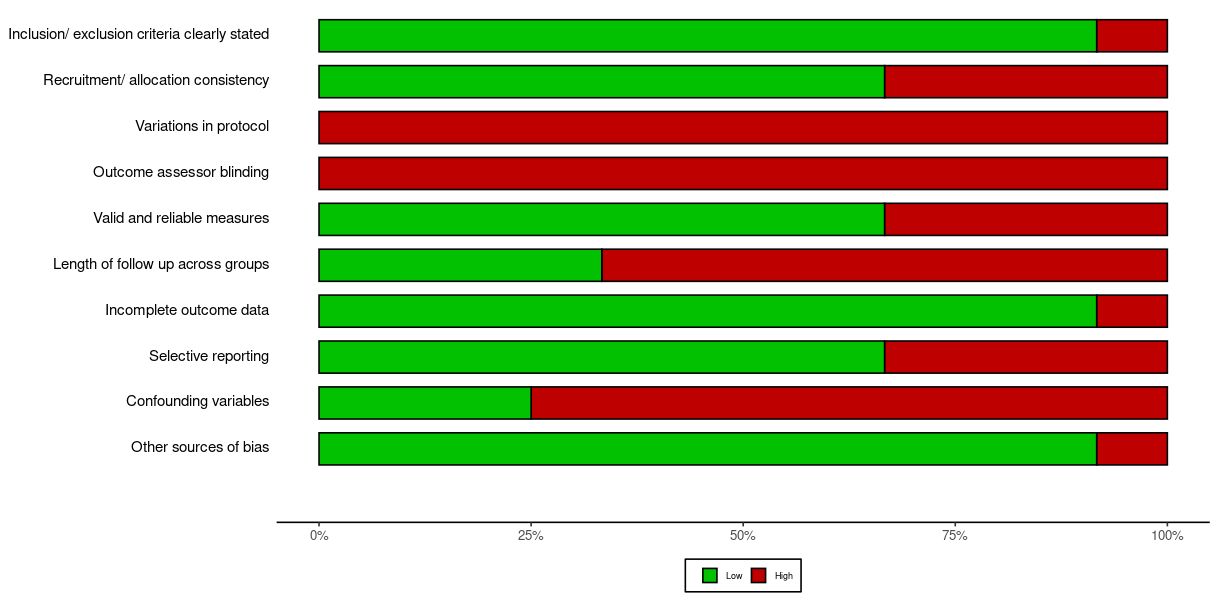*** |
| --- | --- |
| 1. ***More than 1 Frameworks***   **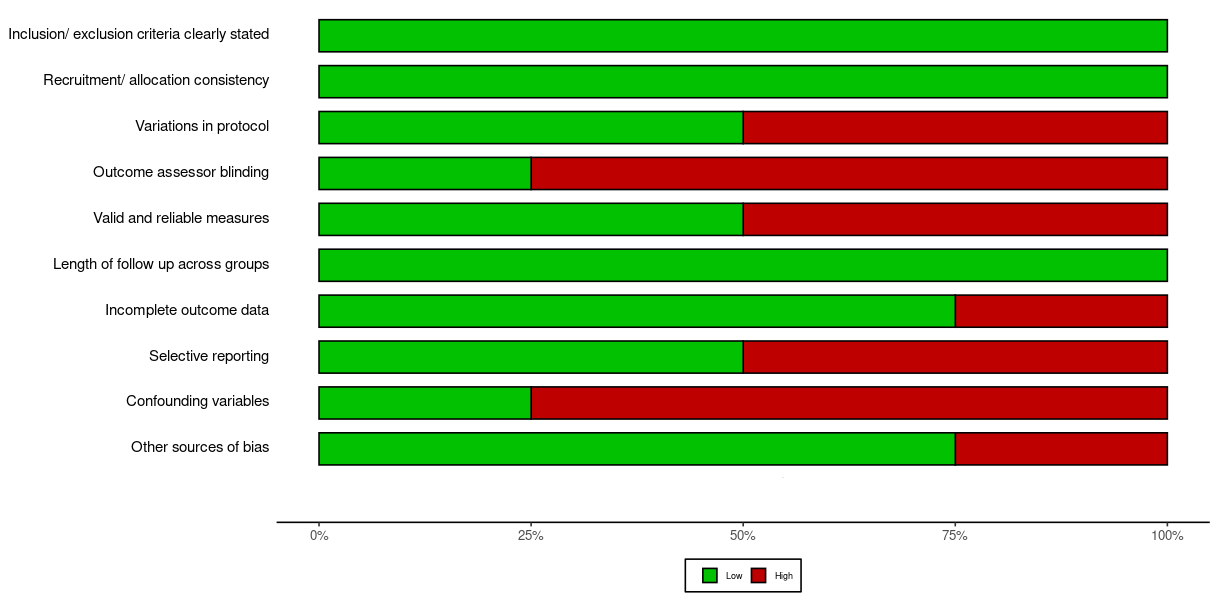** |  |

**Supplemental Figure 3: Screening and Assessment Process of Care Outcomes by Framework Category: Pre-Post study design**

***
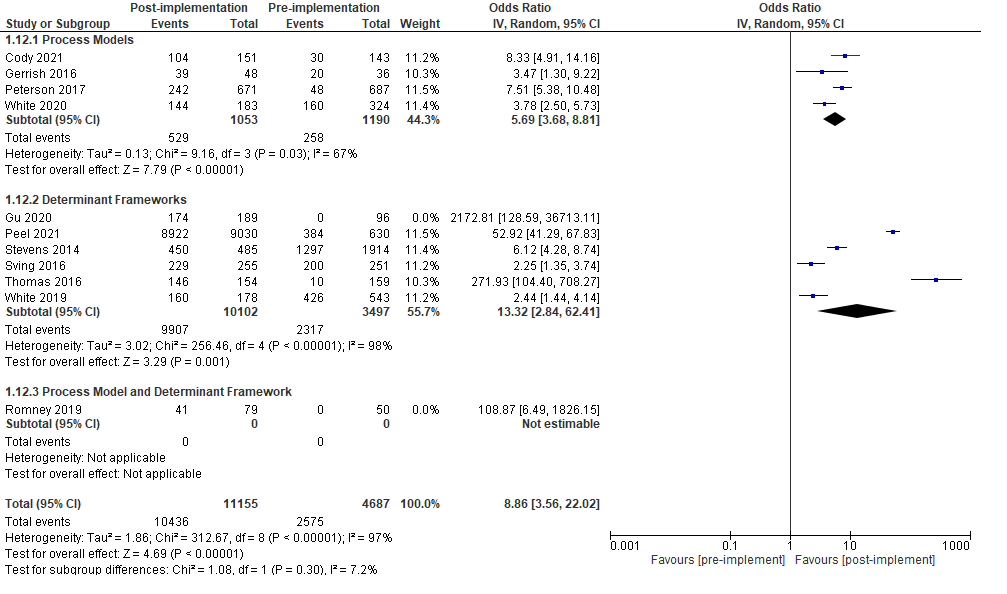
***

***Supplemental Figure 4: Providing Recommended Care Process of Care Outcomes by Framework Category: Pre-Post study design***


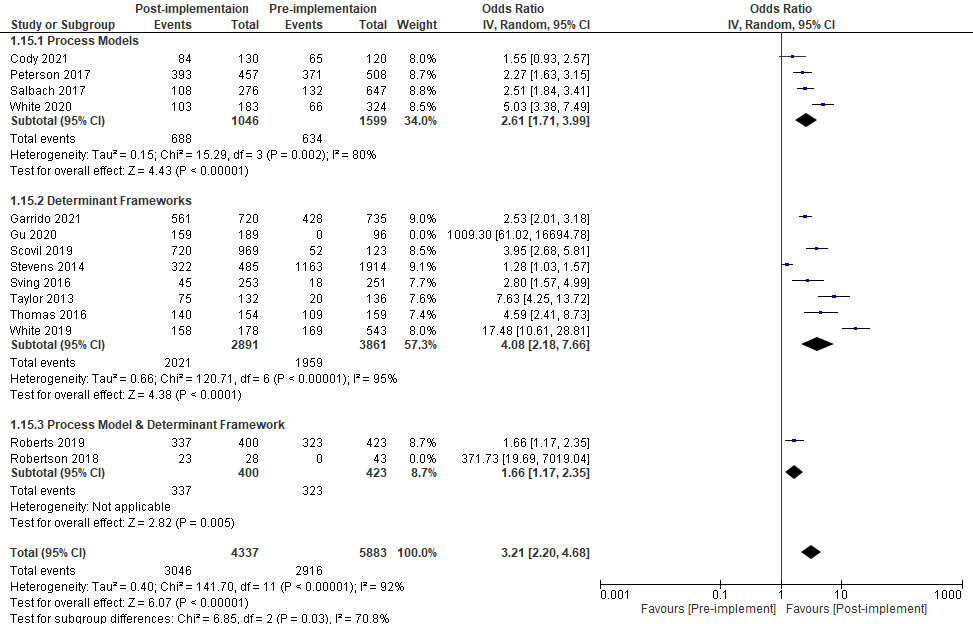


**Supplemental Figure 5: Process of Care Outcomes by Framework Category: Sustainability comparison pre-post study design**


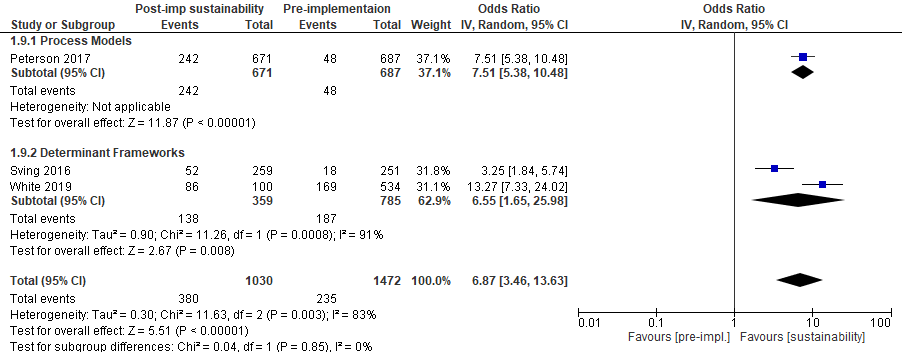

Supplement: Supplementary file 1 — Additional file 1: Supplemental Table 1. Search strategy used for all information sources. Supplemental Table 2. WIDER recommendations checklist. Supplemental Table 3. EPOC taxonomy - Implementation strategies for category: Implementation strategies targeted at healthcare workers. Supplemental Table 4. Template for Intervention Description and Replication (TIDieR) checklist. Supplemental Table 5. List of excluded studies along with reasons for exclusion. Supplemental Table 6. Summary table of included studies. Supplemental Table 7. TIDieR Table. Supplemental Table 8. Risk of bias across included studies. Supplemental Table 9. Adapted WIDER checklist with studies organised by framework category. Supplemental Table 10. Implementation strategies used within individual studies mapped to EPOC taxonomy and scored based on elements from the WIDER checklist. Supplemental Table 11. All process of care outcomes. Supplemental Table 12. All patient outcomes. Supplemental Figure 1. Studies targeting single verses multiply professional groups by framework category. Supplemental Figure 2. Summary plot risk of bias by framework category. Supplemental Figure 3. Screening and assessment process of care outcomes by framework category: pre-post study design. Supplemental Figure 4. Providing recommended care process of care outcomes by framework category: pre-post study design. Supplemental Figure 5. Process of care outcomes by framework category: sustainability comparison pre-post study design. [file 12913_2023_9609_MOESM1_ESM.docx]
